# Design, synthesis and structure-activity relationship (SAR) studies of an unusual class of non-cationic fatty amine-tripeptide conjugates as novel synthetic antimicrobial agents

**DOI:** 10.3389/fphar.2024.1428409

**Published:** 2024-08-02

**Authors:** Noelia Hernández-Ortiz, Pedro A. Sánchez-Murcia, Celia Gil-Campillo, Mirian Domenech, Daniel Lucena-Agell, Rafael Hortigüela, Sonsoles Velázquez, María José Camarasa, Noemí Bustamante, Sonia de Castro, Margarita Menéndez

**Affiliations:** ^1^ Instituto de Química-Física “Blas Cabrera” (IQF), Consejo Superior de Investigaciones Científicas (CSIC), Madrid, Spain; ^2^ Instituto de Química Médica (IQM), Consejo Superior de Investigaciones Científicas (CSIC), Madrid, Spain; ^3^ Laboratory of Computer-Aided Molecular Design, Division of Medicinal Chemistry, Otto-Loewi Research Center, Medical University of Graz, Graz, Austria; ^4^ Centro de Investigación Biomédica en Red de Enfermedades Respiratorias (CIBERES), Instituto de Salud Carlos III, Madrid, Spain; ^5^ Departamento Genética, Fisiología y Microbiología, Facultad Ciencias Biológicas, Universidad Complutense de Madrid, Madrid, Spain; ^6^ Centro de Investigaciones Biológicas Margarita Salas (CIB), Consejo Superior de Investigaciones Científicas (CSIC), Madrid, Spain

**Keywords:** antimicrobial agents, ultra-short non-cationic lipopeptides, fatty amines, anionic lipid membranes, *Streptococcus pneumoniae*, *Streptococcus pyogenes*

## Abstract

Cationic ultrashort lipopeptides (USLPs) are promising antimicrobial candidates to combat multidrug-resistant bacteria. Using DICAMs, a newly synthesized family of tripeptides with net charges from −2 to +1 and a fatty amine conjugated to the *C*-terminus, we demonstrate that anionic and neutral zwitterionic USLPs can possess potent antimicrobial and membrane-disrupting activities against prevalent human pathogens such as *Streptococcus pneumoniae* and *Streptococcus pyogenes.* The strongest antimicrobials completely halt bacterial growth at low micromolar concentrations, reduce bacterial survival by several orders of magnitude, and may kill planktonic cells and biofilms. All of them comprise either an anionic or neutral zwitterionic peptide attached to a long fatty amine (16–18 carbon atoms) and show a preference for anionic lipid membranes enriched in phosphatidylglycerol (PG), which excludes electrostatic interactions as the main driving force for DICAM action. Hence, the hydrophobic contacts provided by the long aliphatic chains of their fatty amines are needed for DICAM’s membrane insertion, while negative-charge shielding by salt counterions would reduce electrostatic repulsions. Additionally, we show that other components of the bacterial envelope, including the capsular polysaccharide, can influence the microbicidal activity of DICAMs. Several promising candidates with good-to-tolerable therapeutic ratios are identified as potential agents against *S. pneumoniae* and *S. pyogenes*. Structural characteristics that determine the preference for a specific pathogen or decrease DICAM toxicity have also been investigated.

## 1 Introduction

Bacterial resistance to the most commonly used antibiotics is a major social and economic challenge worldwide. This is largely a consequence of antibiotic overuse, either by misprescribing or overprescribing, causing pathogens to adapt and evolve to drug resistance (Sikorska et al., 2018). In this scenario, engineered USLPs have appeared as a new class of antibacterials ranked among the most promising alternatives to fight resistant pathogenic bacteria both as planktonic cells and biofilms (Makovitzki et al., 2006; Lohan et al., 2013; Mishra et al., 2015; Benke et al., 2017; Koh et al., 2017; Neubauer et al., 2020; Zhong et al., 2021), including ESKAPE group pathogens (Neubauer et al., 2020), and even fungi (Makovitzki et al., 2006; Makovitzki et al., 2007; Greber et al., 2017; Neubauer et al., 2020). Typically, USLPs comprise one fatty acid chain attached to the *N*-terminus of a short cationic peptide (2–6 amino acids long) whose C-terminus is in the amide form. Both components favor the binding and insertion of USLPs into the negatively charged bacterial membrane and determine their antimicrobial activity and mode of action (Sikorska et al., 2018; Makovitzki et al., 2006; Koh et al., 2017; Neubauer et al., 2020; Sikorska et al., 2014; Fa et al., 2022; Stachurski et al., 2022, and references herein). The net charge and the type and position of basic amino acids (frequently arginine or lysine) and fatty acid chain(s) are all relevant for antimicrobial activity (Sikorska et al., 2018; Neubauer et al., 2020).

The bacterial cytoplasmic membrane is crucial for bacterial survival in all cell metabolic conditions, serving as a selective permeability barrier and site for critical cellular processes (Hurdle et al., 2011). USLPs can permeate and, eventually destroy, the cell membrane of bacteria causing damages that are difficult to recover from (Makovitzki et al., 2006; Lin and Grossfield, 2014), and additional modes of action have been found in certain cases (Fang et al., 2014; Zhong et al., 2021). Compared to classical antibiotics, their fast bacteria-killing action reduces the likelihood of microbial resistance, and little evidence of resistance to USLPs has been found (Makovitzki et al., 2007; Lohan et al., 2013; Lohan et al., 2014; Blazewicz et al., 2017; Rounds and Strauss, 2020; Zhong et al., 2021). In addition, their small size makes them more druggable, simplifies their synthesis, facilitates structural optimization, and may reduce the immunogenic response. As with cationic antimicrobial peptides and larger lipopeptides, limitations of USLPs as antimicrobials include systemic toxicity (disruption of lipid bilayers), degradation, and low bioavailability (Laverty et al., 2010; Lohan et al., 2013; Dawgul et al., 2017; Greber et al., 2017; Neubauer et al., 2020). These shortcomings may be circumvented by chemical modification of peptides using natural and non-natural amino acids or peptidomimetics (Laverty et al., 2010; Benke et al., 2017; Neubauer et al., 2020), rational substitution of amino acids (Neubauer et al., 2020), and varying the mode of lipidation (type and number of acyl chains or the attachment mode) (Bisht et al., 2007; Albada et al., 2012; Koh, et al., 2017; Neubauer et al., 2020).

**FIGURE 1:**
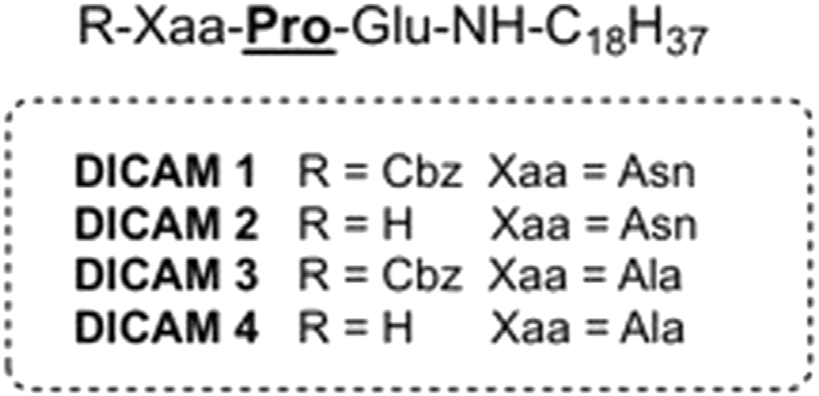
Chemical structure of prototype DICAMs 1–4 conjugated with fatty amines.

Experimental screening of various components of our diverse in-house library of small compounds revealed that lipotripeptides DICAMs 1–4 (Figure 1) displayed a marked antibacterial effect against *S. pneumoniae*, a human pathogen of paramount clinical relevance (GBD, 2017 Causes of Death Collaborators, 2018). Remarkably, DICAMs 1–4 bear either a net negative or a zero charge and a fatty amine chain conjugated to the C-terminal amino acid, instead of being cationic peptides lipidated through the *N*-terminal amino acid or the side-chain of lysine moieties. Specifically, our prototype compounds contain a *C*-terminal Glu residue bound to a stearyl-amine chain, a non-natural D-Pro ( **Pro** ) central amino acid, and a polar (Asn) or neutral (Ala) *N*-terminal residue substituted or not with a benzyloxycarbonyl (Cbz) group. So far, only one study including two negatively charged USLPs that were active on *Streptococcus agalactiae* has been published (Peng et al., 2022). Of note, anionic nonribosomally synthesized lipopeptides like daptomycin or surfactin are active against Gram-positive pathogens in a Ca^2+^ and/or salt concentration-dependent way (Maget-Dana and Ptak, 1992; Maget-Dana and Ptak, 1995; Fiedler and Heerklotz, 2015; Kotsogianni et al., 2021; Maldiney et al., 2021).

The potent anti-pneumococcal activity and structural novelty of our USLP prototype prompted us to investigate the antimicrobial potential of these readily synthetically-available small lipotripeptides. We herein report the solid phase synthesis (SPPS), biological evaluation and SAR studies of a library of USLPs based on the hit compounds 1–4 (Table 1 and Supplementary Table S1). Specifically, our study assessed the effect of varying amino acid sequence, fatty amine length, and *N*-terminal protecting groups’ addition to the activity against selected pathogens, cytotoxicity and hemolysis. DICAM’s ability to permeate large unilamellar vesicles (LUVs) composed of anionic and zwitterionic lipids or disrupt (permeation and depolarization) the bacterial membrane was also investigated. Several membrane-disrupting DICAMs have been identified with high microbicidal activity against *S. pneumoniae* and *S. pyogenes*—another prominent human pathogen (Kanwal and Vaitla, 2023)—and selectivity for pathogens over human cells. Remarkably, most lethal compounds contain a tripeptide that is either anionic or neutral zwitterionic and preferentially interact with and disrupt anionic lipid membranes with a high percentage of PG. This rules out electrostatic interactions as the main driving force for DICAM attachment to bacterial membranes and opens the possibility of additional modes of action besides membrane permeation and depolarization.

**TABLE 1:** General structure, net charge, hydrophobicity and antibacterial activity of DICAMs^a^.

Compound	Formulae	Net charge	Retention time^b^ (min)	Growth halt or lysis (50/100 μM)^c^				
				*Spn*	*Spy* ^*T*^	*Sa* ^*T*^	*Ec*	*Pa*
Reference compounds								
DICAM 1	Cbz-Asn- **Pro** -Glu-NH-C_18_H_37_	−1	21.41	+++	+++	NT	NT	-
DICAM 2	Asn- **Pro** -Glu-NH-C_18_H_37_ ·HCl	0	12.14	+++	+++	++	-/(+)	-/(+)
DICAM 3	Cbz-Ala- **Pro** -Glu-NH-C_18_H_37_	−1	21.42	+++	+++	-	-/(−)	-/(−)
DICAM 4	Ala- **Pro** -Glu-NH-C_18_H_37_ ·HCl	0	19.24	+++	+++	++	-/(+)	-/(+)
C-terminal amino acid modifications and presence and length of fatty amine								
DICAM 5	Asn- **Pro** -Glu-OMe · HCl	0	5.15	-	NT	NT	NT	NT
DICAM 6	Asn- **Pro** -Tyr(Bzl)-NH-C_18_H_37_ ·HCl	+1	22.10	-	-	-	-	-
DICAM 7	Asn- **Pro** -Phe-NH-C_18_H_37_ · HCl	+1	22.21	-	-	NT	NT	NT
DICAM 8	Cbz-Asn- **Pro** -Glu(O^t^Bu)-NH-C_18_H_37_	0	-	-	NT	NT	NT	NT
DICAM 9	Cbz-Asn- **Pro** -Glu-NH-C_12_H_25_	−1	15.80	-	NT	NT	NT	NT
DICAM 10	Cbz-Asn- **Pro** -Glu-NH-C_16_H_33_	−1	18.23	+++	+++	NT	NT	-
DICAM 11	Ala- **Pro** -Glu-NH-C_12_H_25_ · HCl	0	15.29	-	NT	NT	NT	NT
DICAM 12	Ala- **Pro** -Glu-NH-C_16_H_33_ · HCl	0	17.57	+++	NT	NT	NT	NT
DICAM 13	Asn- **Pro** -NH-C_18_H_37_ · HCl	+1	20.18	-	+	-	-	-
DICAM 14	Ala- **Pro** -Ala-NH-C_18_H_37_ · HCl	+1	19.50	+++	+++	+++	-/(−)	-/(−)
DICAM 15	Cbz-Ala- **Pro** -Trp-NH-C_18_H_37_	0	23.18	-	NT	NT	-/(−)	-/(−)
DICAM 16	Ala- **Pro** -Trp-NH-C_18_H_37_ · HCl	+1	21.04	-	-	-	-/(−)	-/(−)
Central amino acid modification								
DICAM 17	Asn- **Ala** -Glu-NH-C_18_H_37_ · HCl	0	18.63	-	-	-	-/(−)	-/(−)
N-terminal amino acid modifications								
DICAM 18	**Pro** -Glu-NH-C_18_H_37_ · HCl	0	19.54	-	NT	NT	-	-
DICAM 19	Cbz-Glu- **Pro** -Glu-NH-C_18_H_37_	−2	21.22	+++	+++	+	-	-
DICAM 20	Glu- **Pro** -Glu-NH-C_18_H_37_ · HCl	−1	18.71	+++	+++	++	-	-
DICAM 21	Cbz-Orn- **Pro** -Glu-NH-C_18_H_37_	0	19.70	+++	+++	+	-	-
DICAM 22	Orn- **Pro** -Glu-NH-C_18_H_37_ · HCl	+1	16.91	+++	++	+	-	-
DICAM 23	**Ala** - **Pro** -Glu-NH-C_18_H_37_ · HCl	0	19.31	+++	+++	++	-	-
DICAM 24	Ac-Ala- **Pro** -Glu-NH-C_18_H_37_	−1	21.23	+++	+++	++	-/(−)	NT

^a^ The abbreviations used are as follows: *Spn, Streptococcus pneumoniae* R6; *Spy*, *Streptococcus pyogenes* ^T^; *sa, Staphylococcus aureus* ^*T*^ *; ec, Escherichia coli* DH10B; *pa, Pseudomonas aeruginosa* PAO1. Bold, underlined residues are D-amino acids.

^b^ Retention time in RP-HPLC, was used to determine DICAM’s hydrophobicity.

^c^ Growth inhibition at 50 and 100 μM of compound based on OD_550_/_600_ variation: (−) no effect; (+++) full inhibition; (++) partial inhibition; (+) growth retardation; NT, not tested. Data in parenthesis refer to the CFU’s decay in the presence of 0.5 mM EDTA (outer membrane permeant) and 100 µM of compound (3 h incubation): (+) CFU, decay ∼1 log-unit; (−) ≤ 0.3 log-units.

## 2 Results

### 2.1 Desing and chemistry of DICAM library

Based on the structures of the hit compounds (DICAMs 1–4) we designed a library of USLPs in which the *C*-terminal and/or *N*-terminal amino acids, the fatty amine length, and *N*-terminal capping was varied, as detailed in Table 1; Supplementary Table S1, to study the effect of charge and sequence modifications on DICAM’s bioactivity. The impact of changing central d-Pro ( **Pro** ) by d-**Ala** (Ala) was also evaluated.

**FIGURE 2:**
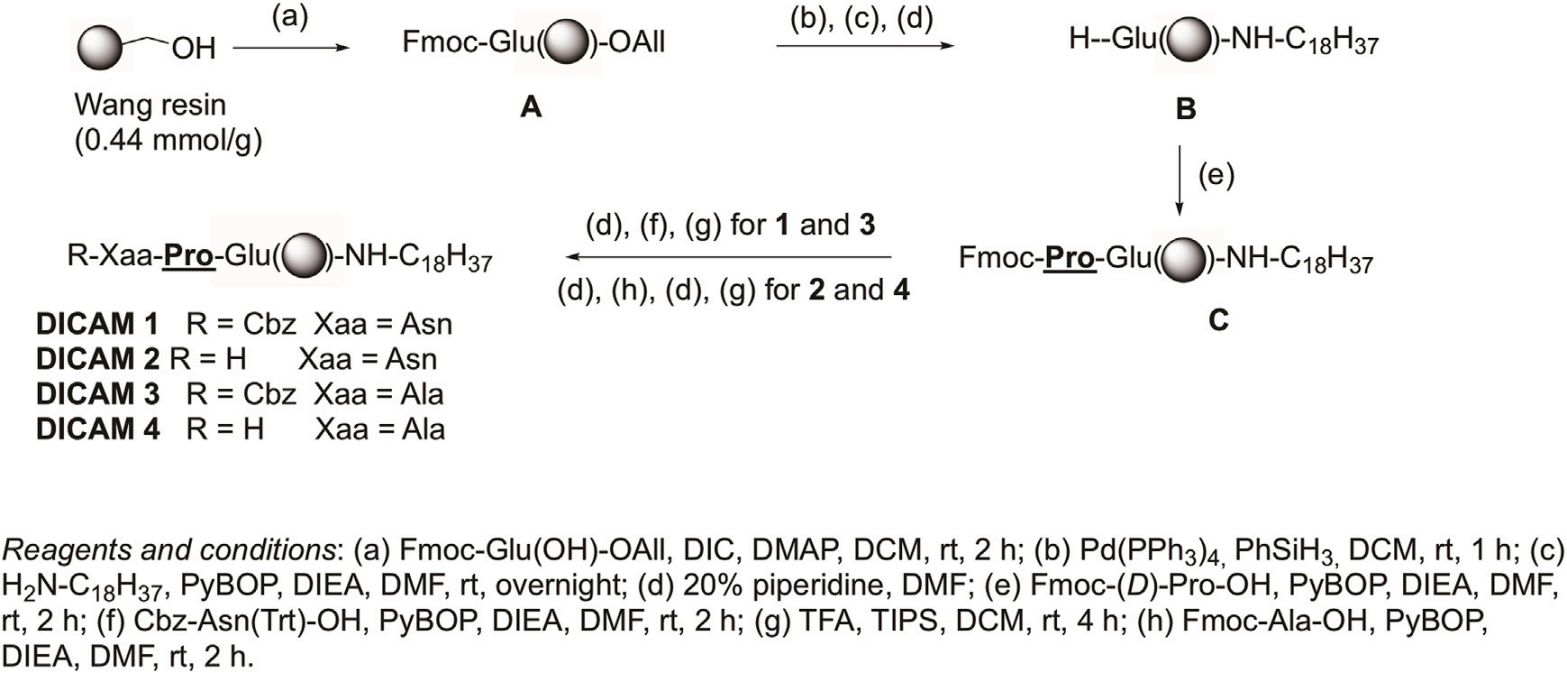
Procedure A for synthesizing compounds with a *C*-terminal Glu is exemplified by preparing prototype DICAMs 1–4. Details of reagents and conditions are given in the figure.

Two different manual SPPS approaches were used for the synthesis of the target lipopeptides, depending on the presence or absence of a Glu residue as *C*-terminal amino acid. The synthetic Procedure A, used for preparation of DICAMs containing a Glu as the *C*-terminal residue, is outlined in Figure 2 and exemplified by the synthesis of prototype DICAMs 1–4. The method involved SPPS protocols using a Wang resin and *N*-Fmoc/allyl chemistry. First, Fmoc-Glu(OH)-OAll was anchored through its side chain to the resin by using *N,N′*-diisopropylcarbodiimide (DIC) and 4-dimethylaminopyiridine (DMAP) as coupling reagent and base, respectively, to give the peptidyl resin **A**. Next, the allyl group was selectively removed with Pd(PPh_3_)_4_ and PhSiH_3_ as an allyl scavenger, and then, octadecyl amine (stearyl amine) was coupled to the *C*-terminal carboxylic group in the presence of benzotriazol-1-yl-oxytripyrrolidinophosphonium hexafluorophosphate (PyBOP) as coupling reagent and diisopropyldiethylamine (DIEA) as base. After Fmoc deprotection under piperidine standard conditions, the coupling of non-natural Fmoc-(*D*)-Pro-OH was similarly carried out with PyBOP/DIEA coupling system. Then, on one hand, coupling of the *N*-deprotected dipeptidyl resin to Cbz-Asn(Trt)-OH or Cbz-Ala-OH under similar PyBOP/DIEA coupling conditions, followed by standard cleavage from the resin with TFA in the presence of trisopropylsilane (TIPS) as cation scavenger was performed. Final purification of the crude compounds afforded the desired *N*-protected lipopeptides DICAM 1 and DICAM 3 in 50% and 68%, respectively. On the other hand, coupling with the appropriated Fmoc-Xaa-OH followed by standard Fmoc deprotection, TFA/TIPS cleavage from the resin, chromatographic purification, and final lyophilization of the pure compounds in the presence of HCl yielded the *N*-deprotected lipopeptides DICAM 2 and DICAM 4 as hydrochloride salts in 42% and 40% yields, respectively. Following general Procedure A we prepared other Glu-containing lipotripeptides (DICAMs 8–12 and DICAMs 17–24) bearing modifications at different parts of the molecule. The synthesis and characterization of all these compounds are detailed in the Materials and Methods section and Supplementary Section S1.1.1.

**FIGURE 3:**
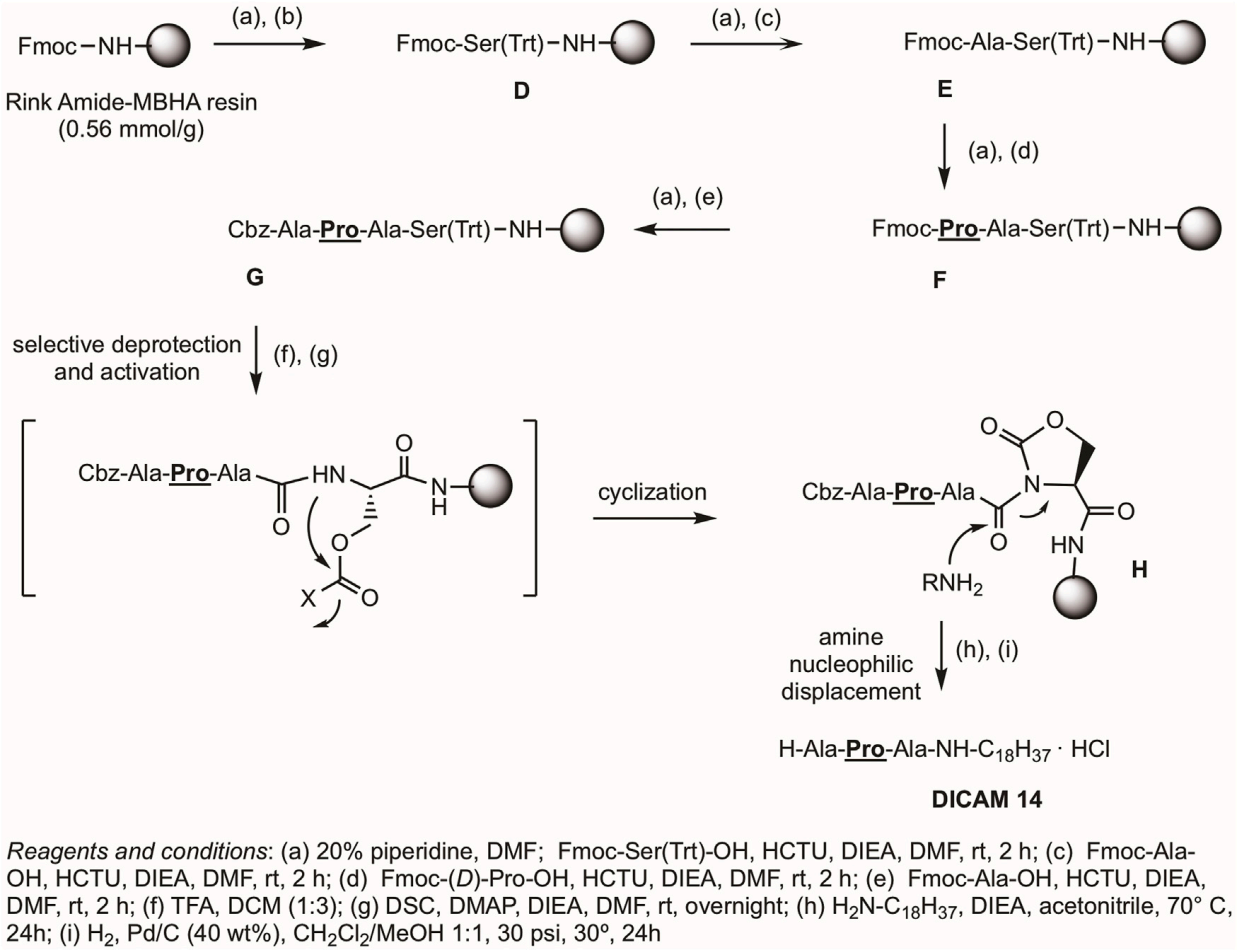
Procedure B uses the cyclic urethane technique (Elashal et al., 2016) for the synthesis of lipotripeptide derivatives without Glu at the *C*-terminus, as exemplified by the synthesis of DICAM 14. Details of reagents and conditions are given in the figure.

Alternative Procedure B, outlined in Figure 3, was used for the syntheses of DICAMs without Glu as C-terminal residue and is exemplified for preparation of DICAM 14, which bears a *C*-terminal Ala residue. Among the reported strategies for preparing *C*-terminal modified peptides, we selected the novel cyclic urethane technique (Elashal et al., 2016) because it avoids using special resins, linkers, or handles. It involved the use of standard Fmoc SPPS on a Rink Amide resin, anchoring a conveniently protected Ser(Trt) as the *C*-terminal residue. After elongation of the desired peptidic sequence, selective Trt deprotection followed by side-chain activation gave a cyclic urethane moiety. Finally, nucleophilic displacement of the cyclic urethane with the appropriated amine as nucleophile would release the desired *C*-terminal modified peptide from the solid support. As shown in Figure 3, after Fmoc deprotection of the Rink amide resin under standard piperidine conditions, Fmoc-Ser(Trt)-OH was attached in the presence of the aminium-based coupling reagent HCTU, and DIEA as the base to give peptidyl resin **D**. The coupling of the three following appropriated amino acids (Fmoc-Xaa-OH as *N*-terminal residue, and Fmoc- **Pro** -OH or Fmoc-Yaa-OH as C-terminal residues) was carried out under similar conditions, to afford the Cbz-tetrapeptidyl resin **G**. Once the elongation was completed, the trityl protecting group from Ser was selectively removed with TFA:DCM (1:3). Next, activation of the hydroxymethyl group of Ser with *N,N′*-disuccinimidyl carbonate (DSC) in the presence of DIEA/DMAP, followed by intramolecular nucleophilic attack of the amide nitrogen of the activated intermediate produced the cyclic urethane ring peptidyl resin **H**. Finally, nucleophilic release upon treatment with octadecylamine in the presence of DIEA and, then, standard Cbz deprotection generated the *N*-deprotected DICAM 14 as a hydrochloride salt, after Biotage purification and lyophilization with HCl (51% yield). Aromatic-containing compounds (DICAMs 7, 15, and 16) and DICAM 13 were also prepared following Procedure B. The syntheses and characterizations are detailed in Materials and Methods and Supplementary Section S1.1.2.

### 2.2 Growth inhibition and bacterial clearance

Planktonic cultures of selected pathogens (*S. pneumoniae* R6, *S. pyogenes* ^T^, *Staphylococcus aureus* ^T^, *Escherichia coli* DH10B, and *Pseudomonas aeruginosa* PAO1 strains) on early logarithmic-phase (T1) were assayed for their sensitivity to a broad range of DICAM compounds, as indicated in Table 1. Their bacteriostatic or lytic effect was first tested by measuring the variation of the OD_550/600_ as a function of time (10 h) when (50/100 μM) DICAMs were added, compared to untreated controls. The screening results, summarized in Table 1, evidenced that a potent antimicrobial activity may exist even when the negative charge of the peptide was higher than in the prototype compounds.

#### 2.2.1 Gram-positive pathogens

**FIGURE 4:**
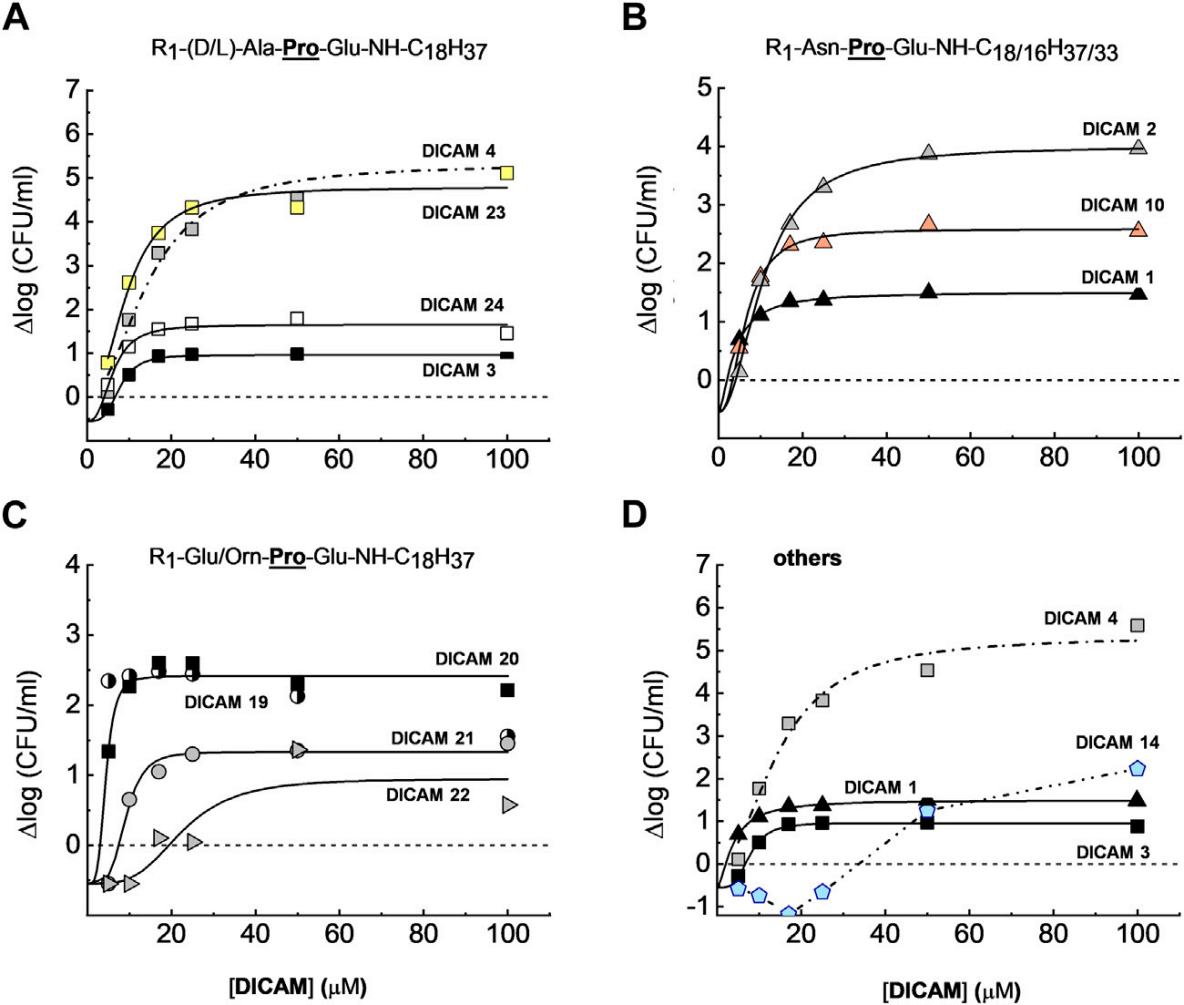
Dose-dependence of *S. pneumoniae* D39 strain viability after treating the cultures with active DICAMs for 1 h (C + Y; 37°C) compared to untreated samples (controls). Killing curves grouped by the type of *N*-terminal amino acid are shown in **(A)** (Ala/Ala); **(B)** (Asn); and **(C)** (Orn or Glu). Panel **(D)** compares killing by compounds with Glu or Ala as C-terminal amino acids. R_1_ is H, Cbz or Ac. Continuous and dashed-dotted curves depict the fitting of the Hill equation to the experimental data (see Section 5.2.3.1). Δlog_max_ is the difference between the upper (Δlog_*f*_) and lower (Δlog_*0*_) limits of Δlog, and SC is the concentration of DICAM at which Δlog = 0 (bacterium population do not grow nor die).

**FIGURE 5:**
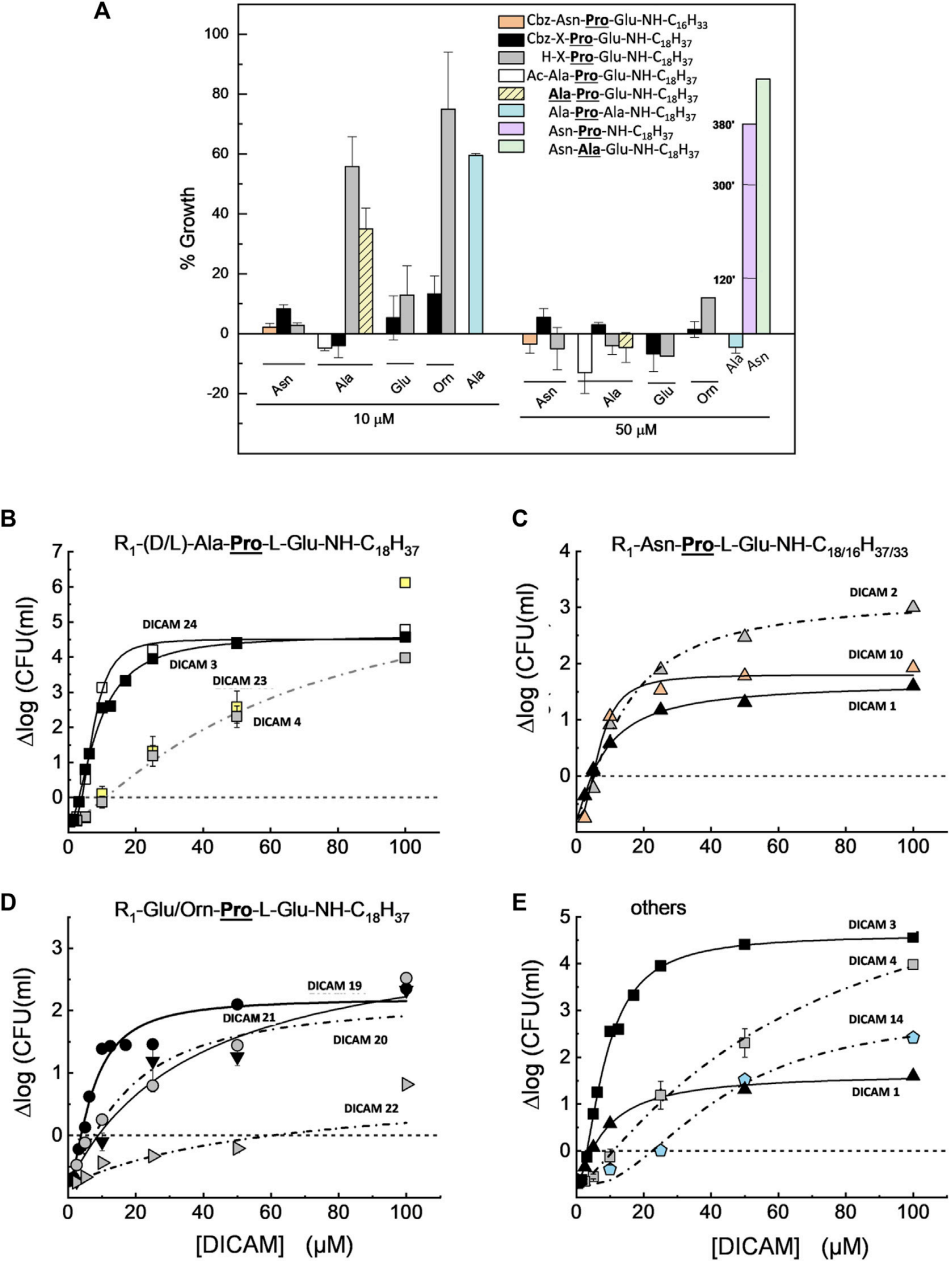
Growth inhibition and dose-dependence of *S. pyogenes* ^T^ viability in cultures treated with active DICAMs for 2 h (37°C, BHI medium) compared to untreated controls. Data represent the average of several assays, with error bars indicating the standard deviation of the mean. **(A)** Percentage of growth upon treatment of cultures with 10 and 50 μM of compound, based on the increment of OD_550_ values of treated vs. untreated samples. Halting of growth by DICAM 13 is given at 120, 300 and 380 min (violet bar). **(B–D)** Dependence of bacterial viability on DICAM dose grouped by *N*-terminal amino acid type: **(A)** (Ala/Ala); **(B)** (Asn); and **(C)** (Orn or Glu). Panel **(E)** compares the killing effects of compounds with Glu or Ala as C-terminal amino acids. Continuous and dashed-dotted curves depict the fitting of Hill equation to the experimental data. R_1_ means as in Figure 4, and SC is the concentration of DICAM at which ∆log = 0.

**FIGURE 6:**
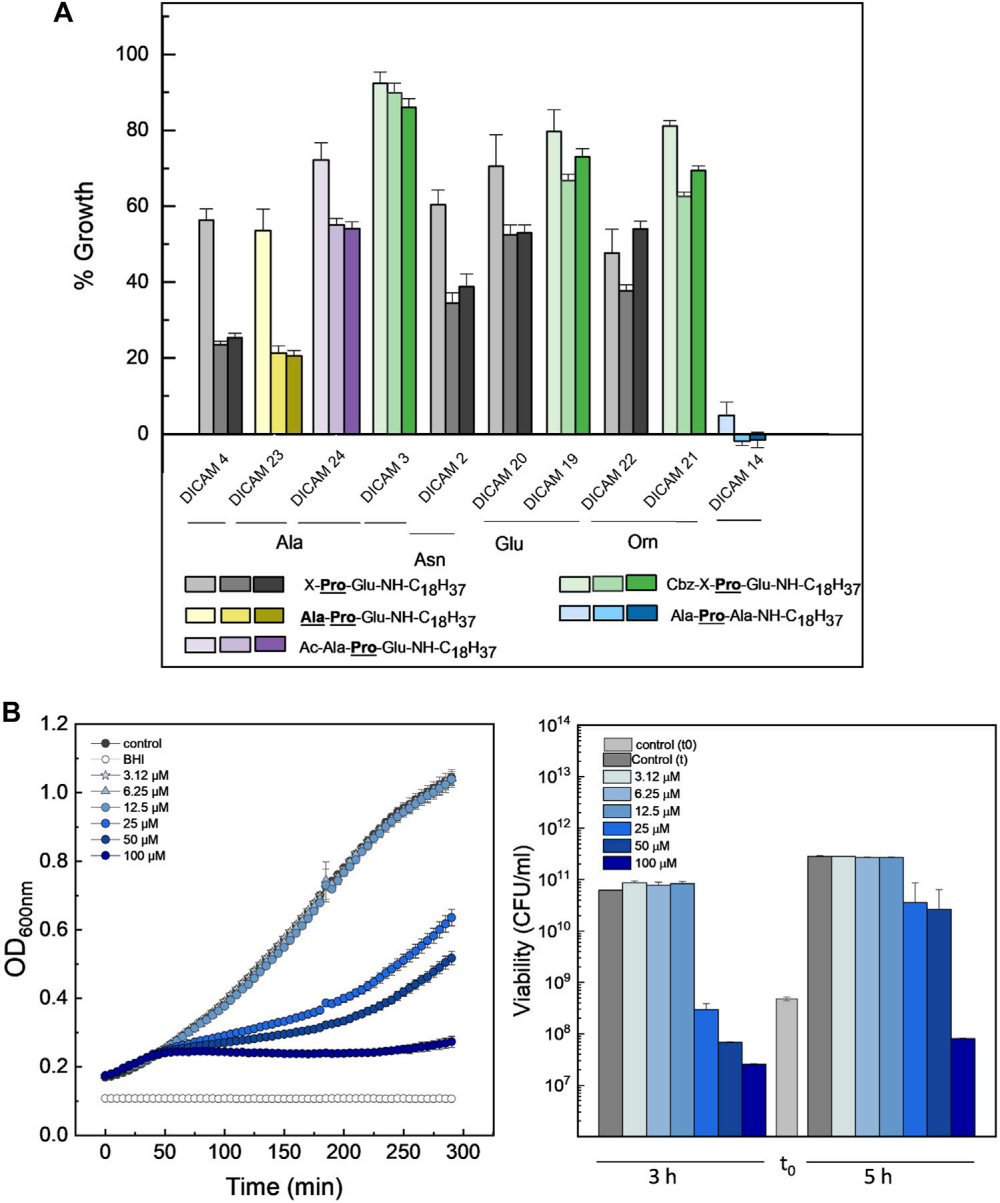
Antimicrobial effect of DICAMs active on *S. aureus* ^T^. **(A)** Growth inhibition after culture challenge at 37°C with 100 μM of compound for 2, 5, and 6.5 h in BHI. Bars represent the percentage of the increment in OD_600_ for treated samples compared to controls and color goes from light to dark as time increases. “X” represents Ala, Asn, Glu or Orn. **(B)** Dependence of the kinetics of growth (OD_600_) (left panel) and bacterial population (right panel) on DICAM 14 dose. Bars in light and dark grey show bacterial populations of the initial inoculum and after incubation (3 and 5 h, respectively). Data shown are the average of three replicates, with error bars indicating the standard deviation of the mean.

The initial screening revealed some of the requirements for DICAMs to be active against the Gram-positive bacteria tested (Table 1). First, the substitution of the fatty amino chain of 18 carbon atoms of the reference compound DICAM 2 by either an OMe group (methylated tripeptide DICAM 5) or an amine with 12 carbon atoms (DICAM 9) abolished the activity against all three pathogens. However, the activity remained intact when the chain of the fatty amine of prototypes DICAMs 1 and 4 was two carbon atoms shorter (DICAM 10 and DICAM 12, respectively), based on *S. pneumoniae* and *S. pyogenes* results. Second, replacing **Pro** with d-Ala (**Ala**) as second amino acid in DICAM 17 also eliminated the antibacterial activity displayed by the prototype DICAM 2 against the three pathogens. Third, Glu and Ala were tolerated as *C*-terminal residues of the lipotripeptides. However, the attachment of an OBut moiety to the Glu side chain (DICAM 8) or the introduction of an aromatic residue at that position (DICAMs 6, 7, 15, and 16) made the compounds inactive against the tested pathogens, despite that aromatic residues commonly favor the antimicrobial activity of lipo/peptides (Wimley and White, 1996; Mishra et al., 2017; Neubauer et al., 2020). In addition, deletion of the C-terminal Glu (DICAM 13) eradicated the activity against *S. pneumoniae* and *S. aureus*. However, a delay and small inhibition of *S. pyogenes* ^T^ growth was still observed. Fourth, Glu (DICAMs 19 and 20) and Orn (DICAMs 21 and 22) were tolerated as *N*-terminal residues, with the impact of these modifications being pathogen-dependent. Besides, active DICAMs induced the lysis of pneumococcal cells but not of *S. pyogenes* ^T^ or *S. aureus* ^T^ as demonstrated by the variation of OD_550/600_ with time (see below). SAR studies continued by measuring the dose dependence of DICAM’s activity at T1 with all the compounds found to be active against *S. pyogenes* or *S. aureus* in the initial screening, and all but one compound in the case of *S. pneumoniae*. The OD values of bacterial cultures were monitored after DICAM addition, and the decrease in the number of viable bacteria was measured relative to controls (Figures 4–6; Supplementary Figure S1). As antibacterial efficacy may decline with the bacterium dose in the inoculum (Belley et al., 2009; Udekwu et al., 2009; Luca et al., 2013), the assays were performed using the bacterial concentrations necessary for the fluorescence experiments in Section 2.3 (10^7^–10^8^ CFU/mL). At these high bacterial densities, MICs were determined as the DICAM concentration at which bacteria neither grow nor are killed (the stationary concentration (SC)), following Udekwu et al., 2009. By fitting the logarithmic plot of cell viability decrease vs. the DICAM dose to a Hill function (see Section 5.2.3.1) we were able to determine the maximum variation in viability (Δlog_max_ = ∆log_*f*_ - ∆log_*0*,_ where ∆log_*f*_ and ∆log_*0*_ are the upper and lower limits of Δlog) in the assay conditions, the SC concentration (Δlog = 0), the dose required for half-variation in Δlog_max_ (*K*_1/2_), and the cooperativity of the antimicrobial effect (*N*_H_). Remarkably, all the compounds active against *S. pneumoniae* also killed *S. pyogenes* ^T^ but not *S. aureus* ^T^. However, the inhibition and/or killing efficiencies varied significantly among pathogens.

*S. pneumoniae* D39 was extremely susceptible to DICAMs 2, 4, 12 and 23 (Figure 4A; Supplementary Figure S1). DICAMs 2, 4 and 23 differ in the *N*-terminal amino acid (Asn, Ala, or **Ala**, respectively), whereas DICAM 12 has a shorter fatty amine chain (16 carbon atoms) compared to DICAM 4. When using saturating doses, they reduced the number of viable cells by ≥4.6 log-units within 60 min (Table 2; Supplementary Figure S1). Introducing a Cbz or an acetyl (Ac) group at the *N*-terminus of DICAM 2 and/or DICAM 4 drastically reduced the Δlog_max_ values of the respective derivatives (DICAMs 1, 3, and 24). However, the *K*_1/2_ values of the modified compounds were comparable to or even lower than those of the unmodified ones, as shown in Table 2. Interestingly, the Hill coefficients varied between 1.5 and 4, which suggests that multiple molecules of DICAMs must cooperate to induce bacterial death.

**TABLE 2:** Dependence of *S. pneumoniae* D39 and *S. pyogenes* ^T^ viability on DICAM’s doses. *K*_1/2_ is the DICAM concentration yielding half-variation in Δlog_max_, and *N*_H_ represents the slope of the Hill function. SC is the concentration of DICAM that prevents both bacterial death and growth (*i.e*., Δlog = 0).

	*S. pneumoniae* D39				*S. pyogenes* ^T^			
Compound General Structure	SC (µM)	Δlog_max_	*K*_1/2_ (µM)	*N*_H_	SC (µM)	Δlog_max_	*K*_1/2_ (µM)	*N*_H_
DICAM 1 Cbz-Asn-**Pro**-Glu-NH-C_18_H_37_·HCl	2 ± 0.2	2.05 ± 0.03	3.8 ± 0.2	1.5 ± 0.1	4.4 ± 0.8	2.5 ± 0.1	8.3 ± 0.9	1.2 ± 0.1
DICAM 2 H-Asn-**Pro**-Glu-NH-C_18_H_37_·HCl	4.1 ± 0.6	4.6 ± 0.1	10.7 ± 0.4	2.1 ± 0.2	5 ± 2	3.9 ± 0.4	14 ± 2	1.4 ± 0.3
DICAM 3 Cbz-Ala-**Pro**-Glu-NH-C_18_H_37_·HCl	6.5 ± 0.5	1.51 ± 0.04	7.8 ± 0.4	3.7 ± 0.5	3.0 ± 0.4	5.4 ± 0.1	8.1 ± 0.4	1.7 ± 0.1
DICAM 4 H-Ala-**Pro**-Glu-NH-C_18_H_37_	4 ± 1.5	6.0 ± 0.4	13 ± 2	1.8 ± 0.3	10 ± 4	6.8 ± 0.8	51 ± 10	1.2 ± 0.1
DICAM 10 Cbz-Asn-**Pro**-Glu-NH-C_16_H_33_·HCl	3.4 ± 0.5	3.13 ± 0.06	6.5 ± 0.3	2.3 ± 0.3	5 ± 1	2.6 ± 0.1	6.8 ± 0.7	2.4 ± 0.5
DICAM 14 H-Ala-**Pro**-Ala-NH-C_18_H_37_·HCl	∼33	-	-	-	22 ± 10	3.7 ± 0.8	41 ± 10	2.1 ± 0.9
DICAM 19 Cbz-Glu-**Pro**-Glu-NH-C_18_H_37_·HCl	3.0 ± 0.8	3.0 ± 0.2	∼5	∼4	3.8 ± 0.9	3.0 ± 0.2	7.1 ± 0.8	1.6 ± 0.3
DICAM 20 H-Glu-**Pro**-Glu-NH-C_18_H_37_·HCl	3.0 ± 0.7	3.0 ± 0.1	4.4 ± 0.6	∼4	8 ± 2	∼3.0	17 ± 4	1.3 ± 0.3
DICAM 21 Cbz-Orn-**Pro**-Glu-NH-C_18_H_37_·HCl	8 ± 1	1.9 ± 0.1	9 ± 1	4 ± 1	∼10	4 ± 2	38 ± 40	1.0 ± 0.5
DICAM 22 H-Orn-**Pro**-Glu-NH-C_18_H_37_·HCl	20 ± 8	1.4 ± 0.4	22 ± 8	∼4	-	-	-	-
DICAM 23 H-Ala-**Pro**-Glu-NH-C_18_H_37_·HCl	2.9 ± 0.8	5.4 ± 0.2	8.6 ± 0.3	2.0 ± 0.4	10 ± 4	6.8 ± 0.8	51 ± 10	1.2 ± 0.1
DICAM 24 Ac-Ala-**Pro**-Glu-NH-C_18_H_37_·HCl	4 ± 0.8	2.2 ± 0.1	6.1 ± 0.6	2.7 ± 0.7	4.0 ± 0.6	5.3 ± 0.1	7.2 ± 0.4	3.0 ± 0.4

*Δlog values represent the difference in the number of CFU/mL of untreated vs. treated cultures in logarithmic units.

Pneumococcal killing also decreased when the *N*-terminal amino acid was Glu (DICAMs 19 and 20) and even more with Orn (DICAMs 21 and 22) (Figure 4; Table 2). However, protection with Cbz marginally affected the antimicrobial capacity of Glu derivatives, based on SC values (≈3 µM) and bacterial viability reduction (>99.5% within 60 min at ≈ 5 μM (DICAMs 19) and 10 μM (DICAM 20)). Contrarily, the killing rate by DICAM 21 was markedly higher than that of DICAM 22, for which much greater concentrations were needed to inhibit growth and cause lysis (Figure 4C; Table 2). The same behavior was found for DICAM 14 which has Ala instead of Glu as the third amino acid of the tripeptide (Figure 4D; Table 2). Besides, at equal concentrations, the most active compounds killed more bacteria and did so faster, as shown in Supplementary Figure S1. Thus, DICAM 4 practically sterilizes the cultures within detection limits after 60 min of incubation with the drug (≥99.999% cell killing; Supplementary Figure S1), while DICAMs 1, 3, or 10 require 2–2.5 h to reduce the viability by 99.99% (≈90% decrease in 1 h; Supplementary Figure S1F).

In contrast to the variability found in Δlog_max_ values, eight out of the twelve compounds active against *S. pneumoniae* have SC values ranging from 2 to 4 µM (Table 2). Among others, they include DICAMs 1, 4 and 19 whose Δlog_max_ values differed, however, by about two orders of magnitude. Moreover, only the two compounds with the lowest Δlog_max_ values (DICAMs 14 and 22) have SC values one order of magnitude higher. Altogether, these results suggest that DICAMs may have different requirements for effectiveness as bactericidal or bacteriostatic agents against *S. pneumoniae*.

The studies on *S. pyogenes* ^T^ revealed that DICAMs with an Ac or a Cbz group at the *N*-terminus were, generally, more potent inhibitors against this pathogen than unblocked ones, in particular when the unsubstituted amino acid was Ala, **Ala** or Orn, as in DICAMs 4, 23 and 22, respectively (Figure 5A). Except for these three compounds and DICAM 14, all markedly active DICAMs reduced *S*. *pyogenes* growth by 88–100% at 10 µM of compounds or less (Figure 5A; Table 2) while, at 50 μM, only the two marginally active ones (DICAMs 13 and 17) allowed for prominent culture growth. Results for the dose-dependence of DICAM killing on *S. pyogenes* ^T^ are summarized in Figure 5B–E; Table 2. Again, no correlation was found between the SC values and bactericidal activities, which further suggested that the requirements to arrest growth and kill bacteria were probably different. The most efficient compounds against *S. pyogenes* ^T^ were DICAM 3 and DICAM 24 (Cbz- and Ac-derivatives with an *N*-terminal Ala), followed by DICAM 2 (the unblocked derivative of DICAM 3). All three have SC estimates of 3–5 µM and can decrease the number of viable bacteria in the inoculum by 99.9–99.99% within 2 h (Figures 5B, C; Tables 2, 3). In the same range of SC values (4−5 µM) were also those of the Cbz derivatives of Asn (DICAMs 1 and 10) and Glu (DICAM 19), although their killing effects were about one order of magnitude lower (Figure 5C, D; Table 2). Besides, DICAMs 4, 14 and 23 showed lower effectiveness as inhibitors and required much higher concentrations of compound to achieve bacterial population reductions comparable to those of respective Cbz- or Ac-derivatives tested (Figure 5B, E). This effect was even higher when the first amino acid was ornithine (Figure 5D), and indicated that these four compounds primarily acted as bacteriostatic agents against *S. pyogenes* ^T^ when present in low-to-moderate concentrations. Hill coefficients ranged from 1.2 to 3 and were generally lower than those for *S. pneumoniae*.

**TABLE 3:** Hemolytic activity and cytotoxicity of active DICAMs. HC_50_ and IC_50_ values are the concentrations required to produce 50% hemolysis or 50% inhibition of cell growth, respectively, and IC_50av_ is the mean of the three IC_50_ values. SI represents the selective index defined as HC_50_/SC or IC_50av_/SC.

Compound	HC_50_ (μM)	IC_50_ (µM)			SI_*Spy*_		SI_*Spn*_	
		A549	IMR-90	HeLa	HC_50_/SC	IC_50av_/SC	HC_50_/SC	IC_50av_/SC
DICAM 1 Cbz-Asn-**Pro**-Glu-NH-C_18_H_37_	12.5 ± 0.6	45 ± 6	39 ± 1	29 ± 2	2.8	8.6	6.25	18.8
DICAM 2 Asn-**Pro**-Glu-NH-C_18_H_37_	8.0 ± 0.3	42 ± 5	46.7 ± 0.7	42 ± 2	1.6	8.7	2	10.6
DICAM 3 Cbz-Ala-**Pro**-Glu-NH-C_18_H_37_	11 ± 3	59.2 ± 0.7	75 ± 13	49.2 ± 1.5	3.7	20	1.7	9.4
DICAM 4 Ala-**Pro**-Glu-NH-C_18_H_37_	7 ± 2	≥25	65 ± 3	32 ± 2	0.7	4	1.75	10
DICAM 10 Cbz-Asn-**Pro**-Glu-NH-C_16_H_33_	11 ± 1	35 ± 2	45 ± 1	28.0 ± 0.7	2.2	7.2	3.2	10.6
DICAM 14 Ala-**Pro**-Ala-NH-C_18_H_37_	36 ± 1	8.0 ± 0.3	7.69 ± 0.06	6.3 ± 0.7	1.6	0.33	1	0.2
DICAM 19 Cbz-Glu-**Pro**-Glu-NH-C_18_H_37_	120 ± 30	45 ± 2	44 ± 1	46 ± 2	31.6	11.8	40	15
DICAM 20 Glu-**Pro**-Glu-NH-C_18_H_37_	6 ± 2	91 ± 2	78.6 ± 0.1	59.2 ± 0.1	0.75	9.5	2	25.4
DICAM 21 Cbz-Orn-**Pro**-Glu-NH-C_18_H_37_	12 ± 3	-	86 ± 15	-	1.2	8.6	1.5	10.7
DICAM 22 Orn-**Pro**-Glu-NH-C_18_H_37_	11 ± 3	59.6 ± 0.9	58.0 ± 0.4	57.2 ± 0.4	-	-	0.6	2.9
DICAM 23 **Ala**-**Pro**-Glu-NH-C_18_H_37_	6 ± 2	80.6 ± 0.2	57 ± 2	13.4 ± 0.9	0.6	5	2	16.7
DICAM 24 Ac-Ala-**Pro**-Glu-NH-C_18_H_37_	9 ± 1	40 ± 3	43.8 ± 0.6	28.4 ± 0.2	2.3	9.3	2.2	9.3

Errors in HC_50_ and IC_50_ values are standard deviations of data from at least three independent experiments.

A much lower antimicrobial effect was observed on *S. aureus* ^T^. In tested compounds, alanine was preferred over the other three amino acids as the first residue to inhibit bacterial growth (Ala/**Ala** > Asn > Glu ≥ Orn), and the presence of an Ac- or a Cbz-group further reduced their inhibitory effect (Figure 6A). Remarkably, the substitution of the C-terminal Glu by Ala makes DICAM 14 the only derivative able to fully arrest the growth of *S. aureus* (Figure 6A), in sharp contrast to its behavior on *S. pyogenes* and *S. pneumoniae*. However, quantification of dose-dependent lethality showed that DICAM 14 only caused a permanent fall in bacterial survival at 100 µM compound (≈85% after 5 h incubation), with its main effect being to delay the growth of the culture at lower concentrations (Figure 6B). DICAMs 4 and 23, the next following in activity, reduced *S. aureus* ^T^ growth down to ≈25% of controls, respectively, after five- or more hours of incubation at 100 µM compound (Figure 6A).

**FIGURE 7:**
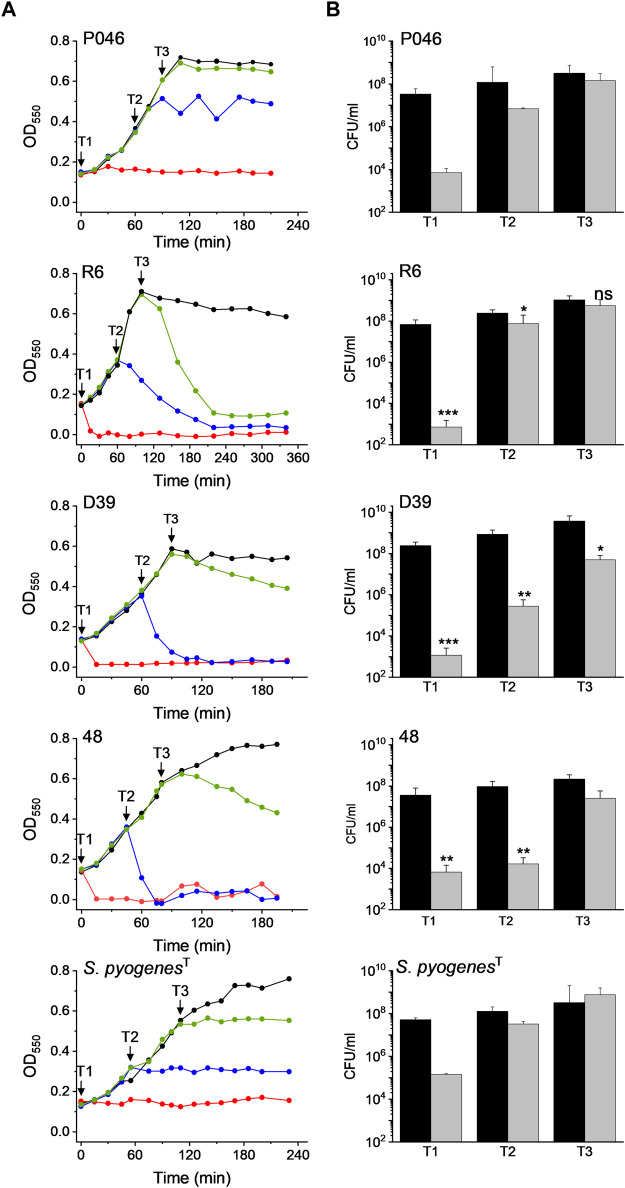
Dependence of DICAM 4 activity on the state of growth of *S. pneumoniae* and *S. pyogenes* ^*T*^. **(A)** Growth curves of *S. pneumoniae* R6, D39, 48 (MDR) and P046 (*lytA* ^*-*^ and *lytC* ^−^) strains and *S. pyogenes* ^T^. Results are representative of 3–6 independent experiments. Black arrows indicate the addition of 50 µM DICAM 4 at T1 (OD_550_ ≈ 0.14), T2 (OD_550_ ≈ 0.3), and T3 (OD_550_ ≈ 0.6), represented by red, blue and green symbols, respectively. Untreated controls (0.5% DMSO) are in black. Incubations were carried out at 37°C in culture medium. **(B)** Variation of cell viability after 2 h of treatment with DICAM 4 under conditions detailed in **(A)**. The number of viable bacteria in the initial inoculum and treated samples are compared (black and grey bars, respectively). Error bars indicate the standard deviation from two (P046, 48 (T3) and *S. pyogenes* ^T^) or 3–4 independent experiments. Statistical analysis was performed using a two-tailed t-test to compare with controls. Asterisks indicate significant differences (*p < 0.05, **p < 0.01, ***p < 0.001) and “ns” stands for no significant differences.

The effect of DICAMs was also evaluated when they were added at the mid-exponential (T2) and early stationary (T3) phases using several pneumococcal strains and *S. pyogenes* ^T^ along with prototypes DICAMs 3 and/or 4 that showed a reversed preference for these pathogens. The addition of 50 μM DICAM 4 at T2 or T3 arrested the growth of *S. pyogenes* ^T^ and either halted (P046 strain) or triggered the lysis (R6, D39, 48 strains) of pneumococcal cultures (Figure 7). The lysis rate and the bactericidal effect were, however, dependent on the metabolic state of the cell, being particularly reduced when the challenge occurred at T3. Even so, the viability of treated cells after 2-h incubation was significantly reduced, mainly for the two pathogenic strains of *S. pneumoniae* (D39 and the multiresistant strain 48). Bacterial killing increased with the time of exposure, as shown in Supplementary Figure S2 for DICAM 4, whose addition at T2 decreased the viability of D39 cultures ≈ 3- and 5-log units after 30 min and 3 h incubation, respectively, and ≈1.7-log units (≥98% death) in approximately 3 h when added at T3. In contrast, minor changes in culture viability were found at T2 and T3 in *S. pyogenes* ^T^. Additionally, visualization of R6 cultures treated at T2 or T3 with DICAMs 3 and/or 4 using phase-contrast microscopy, in combination with monitoring of live/dead staining of cells treated or not with DICAM 3 by fluorescence microscopy, further confirmed the capacity of these compounds to kill and lyse the bacteria challenged at both stages of growth (Supplementary Figures S3, S4).

On the other hand, strain P046, defective in the two pneumococcal autolysins, was the only variant of *S. pneumoniae* resistant to DICAM-promoted bacteriolysis. This observation pointed towards the major autolysin, LytA as the agent responsible for the lytic phenomenon, as also occurred for the β-lactam- and vancomycin-induced lysis of pneumococci (Tomasz and Waks, 1975). Furthermore, an alteration in the ratio of lipoteichoic acid to teichoic acid, which is known to control LytA activity (Flores-Kim et al., 2022), may have resulted from a DICAM-induced perturbation of the cytoplasmic membrane.

#### 2.2.2 Gram-negative pathogens

DICAM’s activity was also explored on *E. coli* DH10B and *P. aeruginosa* PAO1, focussing on the prototype compounds, DICAMs with *N*-terminal or central amino acid modifications, and DICAMs without a glutamic residue at *C*-terminal position, as showed in Table 1. Addition of tested compounds to the culture medium had little or no effect on bacterial growth, even at a concentration of 100 μM (Table 1). Since inactivity could arise from the inability of DICAMs to cross the outer membrane, we next tested their effect on bacterial suspensions in the absence or the presence of 0.5 mM EDTA, an outer membrane permeating agent. A decrease of approximately 90% in bacterial viability was observed after 3 h treatment with DICAM 2 and DICAM 4, compared to controls in EDTA-containing buffer (Supplementary Figure S5). No variations were found with the other compounds tested (Table 1), showing that only DICAMs containing Ala/Asn-**Pro**-Glu tripeptides were able to access the cell membrane and kill these pathogens when the outer membrane permeability was enhanced.

#### 2.2.3 Antimicrobial activity on biofilms of *S. pneumoniae*

**FIGURE 8:**
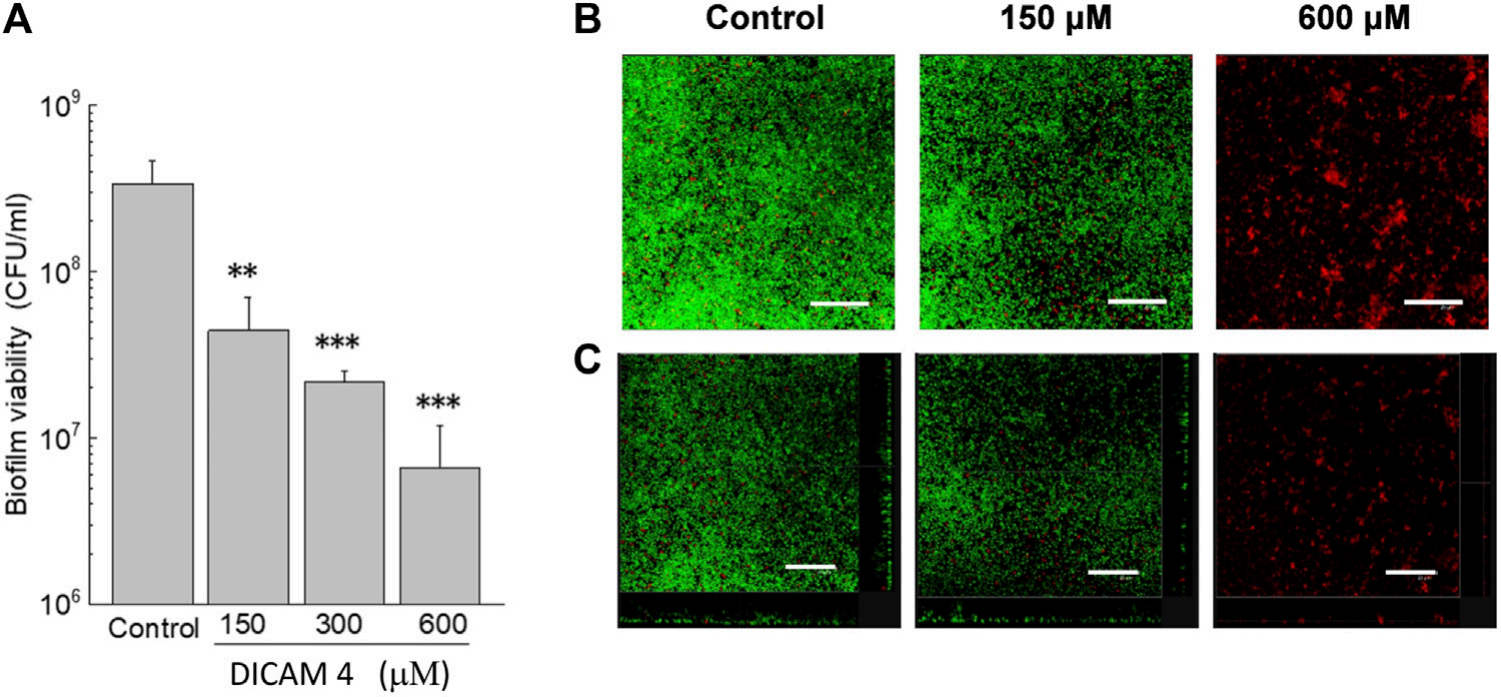
Disaggregation of biofilm *S. pneumoniae* R6 in the presence of DICAM 4. **(A)** Viability of biofilms treated (1 h; 37°C) with 1% DMSO (control) or DICAM 4 at the indicated concentrations, measured by plating on blood agar plates. Data represent the mean ± standard error of three independent experiments. Error bars represent the standard error. Statistical analysis was performed using one-way ANOVA followed by a Dunnett post-test to compare with controls. Asterisks indicate *p* < 0.01 (**) or 0.001(***). **(B)** CLSM images of R6 biofilms showing controls and treatments with 150 and 600 µM DICAM 4. Next, cells of biofilms were stained with the BacLight bacterial viability kit to reveal membrane-intact (green fluorescence) and permeabilized (red fluorescence) bacteria (overlaid images). **(C)** Maximum intensity projections were obtained in the *x*−*y* (individual scans at 0.5 μm intervals) and *x*−*z* (images at 5 μm intervals) planes, and orthogonal CLSM images of control and treated biofilms are shown. A representative region of the *x*–*y* plane over the depth of the biofilm in both the *x*–*z* and *y*–*z* dimensions is shown on the right and bottom of the images. Results are representative of experiments performed in triplicate. In all images, the scale bar equals 25 μm.

The capacity of DICAMs to kill and disperse bacteria grown in sessile form was preliminary evaluated using prototype biofilms of *S. pneumoniae* R6 strain and reference compound DICAM 4. As shown in Figure 8A, the addition of DICAM 4 killed almost 90%–99% of cells in preformed biofilms of the R6 strain at 300 μM and 600 µM of compound, respectively. A drastic reduction in biofilm density and thickness was also observed using confocal laser scanning microscopy (CLSM). In addition to its disintegrating effect on the biofilm, DICAM 4 at 600 μM killed the bacteria that remained attached (Figure 8B).

### 2.3 Bacterial cell membrane perturbation

**FIGURE 9:**
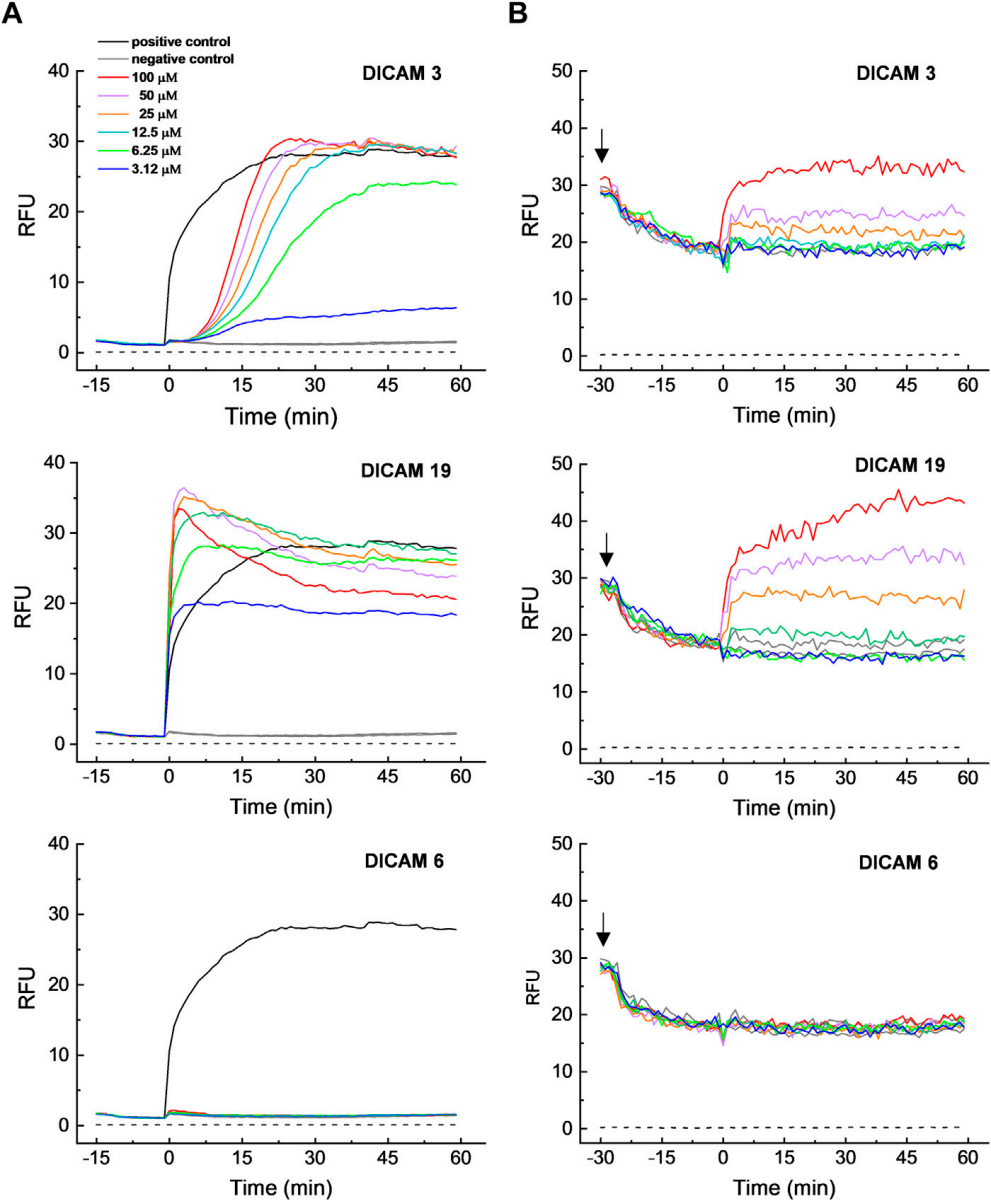
Permeabilization and depolarization of *S. pyogenes* ^T^ membrane by DICAMs. **(A)** Time course profiles of cell membrane permeation to SYTOX Green upon addition (*t* = 0) of DICAMs at the concentrations specified in the figure label (color code). The dye was added to cells in PBS, pH 7.2, and fluorescence only increased when SYTOX Green could cross the membrane and bind DNA. DICAMs were replaced by Triton X-100 (0.01% final concentration) in positive controls, while buffers with or without 0.35% DMSO (due to dye and DICAM vehicle solutions) were used for negative controls. **(B)** Time course of membrane depolarization, monitored by following the increase in DiSC3(5) fluorescence. Bacterial samples in BHI medium were supplemented with DiSC3(5) at the time indicated by black arrows, and the change in the dye fluorescence signal, due to membrane binding and self-quenching (an indication of membrane polarization), was monitored until it was stable. Then, tested compounds at the indicated concentrations were added (*t* = 0) and the loss of membrane potential, and subsequent DisC3(5) dissociation, was measured (color code was as in A). DICAM was replaced by BHI with or without 1.25% DMSO (dye and DICAM vehicle solutions) for negative controls. Dashed lines in all panels represent the background signals. Representative data from independent experiments are shown for both assays.

We confirmed that active DICAMs target the bacterial cell membrane by testing the effects of two negatively-charged, active compounds (DICAMs 3 and 19) and one inactive compound (DICAM 6) on the *S. pyogenes* ^T^ membrane, using DiSC3(5) and SYTOX Green dyes, sensitive to transmembrane potential and permeability, respectively. *S. pyogenes* was chosen as bacterial model because the detection of membrane perturbation would not be interfered with by the lysis of dead cells by DICAMs. The assays were conducted at 10^7^–10^8^ CFU/mL (OD_550_ ≈ 0.2). After adding DICAM 3 or DICAM 19, the transmembrane potential dissipates quickly (within 5–10 min), as evidenced by the rapid increase in the signal of DiSC3(5) as it dissociates from the bacterial membrane (Figure 9B, upper and middle panels). A slightly greater depolarization was observed with DICAM 19 when used at equal concentration. Besides, the kinetic profile at 100 μM DICAM 19, with a slow increase in the fluorescence signal after the initial burst, resembled the kinetics of leakage expected for a hybrid permeation mode (Wimley and Hristova, 2019).

DICAM 3 and DICAM 19 also increased the bacterial membrane permeability, as shown by the increase of the SYTOX Green fluorescence in treated samples. However, their kinetics differed greatly (Figure 9A, upper and middle panels). Permeation by DICAM 3 has a sigmoidal time course, with a dose-dependent lag of more than 7 min before reaching the maximum dose-dependent steady increase in fluorescence intensity. This behavior contrasted with the exponential increase in fluorescence observed in the positive control and most lipo/peptide-induced permeabilizations (Rathinakumar et al., 2009). However, sigmoidal kinetics for membrane disruption have also been reported (Belley et al., 2009; Rathinakumar et al., 2009; Jackman et al., 2015; Boix-Lemoce et al., 2020). Membrane permeation likely occurred through transient pores below 12.5 µM DICAM 3, since the influx of SYTOX green was incomplete but reached a plateau in around 40 min (Wimley, 2015; Wimley and Hristova, 2019). Additionally, the entry of SYTOX Green started later and took longer than the membrane-potential dissipation, which indicates that a sequence of events occurs during *S. pyogenes* ^T^ membrane perturbation by DICAM 3. In contrast, permeabilization by DICAM 19 occurred in a rapid way (Figure 9A, middle panel), yielding a dose-dependent burst in the SYTOX Green signal which was concomitant with the membrane depolarization processes. At DICAM 19 concentrations of 12.5 μM or higher, the burst was followed by some drop in the SYTOX Green signal before attaining the equilibrium value. Similar kinetic profiles have been reported for membrane permeation or depolarization by cationic antimicrobial peptides monitored with SYTOX green or DiSC3(5) (Barns and Weisshaar, 2013; Te Winkel et al., 2016; Vázquez et al., 2022), respectively. As with DICAM 3, the results were compatible with the formation of transient pores at the lowest doses of DICAM 19 assayed. As expected, no depolarization or membrane permeation was observed when *S. pyogenes* ^T^ was treated with DICAM 6 (Figure 9A, B, lower panels), indicating that its lack of antibacterial activity (Table 1) was likely due to its inability to disrupt the membrane integrity.

As shown in Supplementary Figure S6, there is a positive correlation between the dose-dependence of the percentages in membrane permeabilization, growth inhibition (SC values), and cell killing by DICAM 3. However, an equivalent rate of bacterial death by DICAM 19 seems to require a somewhat higher concentration of compound than membrane permeation and growth halting. Furthermore, the significant up-shift observed in the dose dependence of membrane depolarization suggests that dissipation of the membrane potential is not the primary microbicidal mechanism of DICAMs 3 or 19 on *S. pyogenes* ^T^. A similar uncoupling between permeation and depolarization processes was also observed for the action of melittin on *Staphylococcus epidermis* and *S. aureus* (Boix-Lemoce et al., 2020). Moreover, *S. aureus* was permeabilized but not depolarized by the tPMP-1 microbicidal protein (Yeaman et al., 1998).

### 2.4 Permeabilization of calcecin-loaded LUVs

**FIGURE 10:**
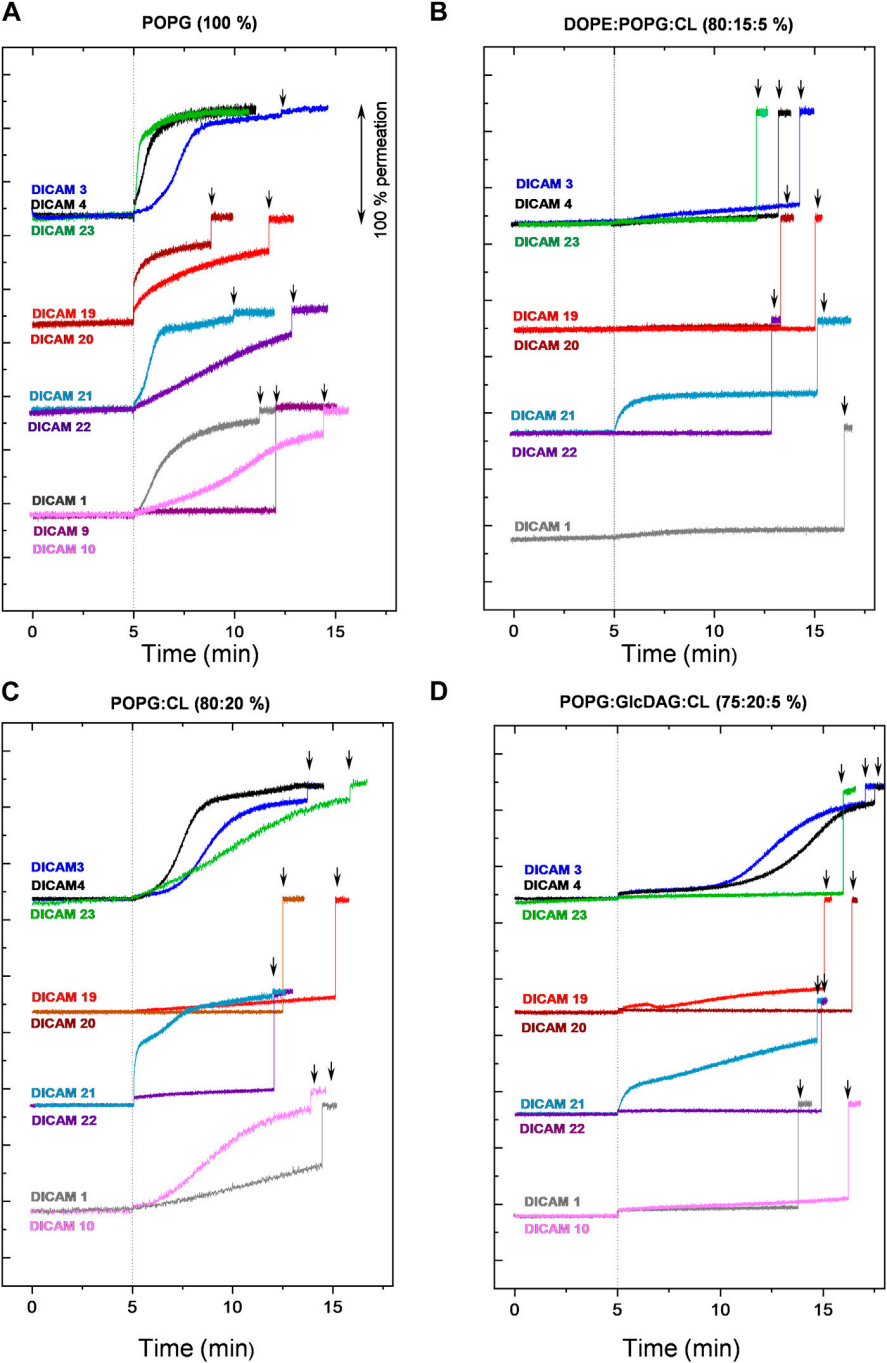
Permeation of LUVs by DICAMs. Kinetics of calcein release from LUVs of different compositions by DICAMs showing antibacterial activity. After recording the basal fluorescence for 5 min, DICAM was added (10 μM final concentration) and the changes in fluorescence (λ_ex_ = 480 nm, λ_ems_ = 550 nm) were monitored and plotted as the percentage of membrane permeabilization relative to the final fluorescence signal recorded after treating LUVs with 0.025% v/v Triton-X100 at the end of the experiment (black arrows). Assays were carried out at 25°C in vesicle buffer (10 mM Tris, 140 mM NaCl, 0.5 mM EDTA, pH 7.4). Detergent-induced permeabilizations of DICAM-untreated LUVs were carried out in parallel. The experimental curves were shifted along the Y-axis for clarity. **(A)** pure POPG, **(B)** DOPE:POPG:CL (80:15:5), **(C)** POPG:CL (80:20), and **(D)** POPG:GlcDAP:CL (75:20:5).

DICAMs were also investigated for their ability to induce the release of calcein entrapped at self-quenching concentration within LUVs whose compositions approximated those of the cell membranes of pathogens tested (Epand and Epand, 2010; Epand et al., 2010). A wide variety of compounds with (DICAMs 1, 3, 4, 10, 19–22, and 24) and without (DICAMs 6–9, 13, 17, and 18) antimicrobial activity were used to gain a better understanding of their effects on bacterial cell membranes and their preferences for lipid membrane composition. DICAMs were added at 10 μM (1.5‒2.5 × SC average values) to the LUV suspension and the kinetics of membrane permeabilization were monitored by following the fluorescence recovery. The results are summarized in Figure 10 and Supplementary Figure S7. All DICAMs with antibacterial activity permeated pure POPG (1-palmitoyl-2-oleoyl-sn-glycero-3-phospho-(1′-rac-glycerol)) vesicles, the time-course profiles of calcein leak varying depending on the compound (Figure 10A). Some permeations showed exponential kinetics, leading to either full (DICAMs 4 and 23) or partial (DICAM 20) leak of calcein, with equilibrium being reached within a few minutes (DICAMs 4 and 23) or at much longer times (DICAM 1, 19 or 22). On the other hand, DICAMs 3, 10 and 21 showed sigmoidal kinetics with different latency periods, such as those observed in *S. pyogenes* permeation by DICAM 3. Inclusion of cardiolipin (CL; heart, bovine cardiolipin; 90% 1ʼ-[1,2-dilinoleoyl-sn-glycero-3-phospho]-3ʼ-[1,2-dilinoleoyl-sn-glycero-3-phospho]-glycerol) in POPG LUVs (POPG:CL, 80:20 mol ratio) slows down or even blocks the permeation to calcein induced by most of these compounds (Figure 10C), favoring in certain cases the appearance of sigmoidal (DICAMs 4 and 23) or biphasic (DICAM 21) kinetics. Calcein leakage was further reduced when the LUVs also contained GlcDAG (1,2-diacyl-3-O-(α-D-glucopyranosyl)-sn-glycerol), (POPG:GlcDAG:CL, 75:20:5) (Figure 10D). This uncharged lipid is found in *S. pyogenes* membranes (40%–55%; Epand et al., 2010) but not in those of pneumococci which are composed of POPG and CL at approximately equal mole ratio (Epand and Epand, 2010). Remarkably, the differential effects observed on LUV permeation in Figure 10C, D correlated with the varying activities of respective DICAMs against these two pathogens, as illustrated, for example, the lower activity of DICAMs 1, 10 and 21 against *S. pyogenes,* or the species-specificity inversion showed by DICAM 3 and DICAM 4 (see also Table 2; Figures 4, 5). On the other hand, only the Orn derivative with a CBz *N*-terminal cap (DICAM 21; zero net charge) was able to permeate and release calcein from LUVs that mimic the lipid composition of the inner membrane of Gram-negative bacteria (DOPE:POPG:CL, 80:15:5 mol ratio; Epand and Epand, 2010) (Figure 10B).

As reported for other USLPs (Makovitzki et al., 2006), some DICAMs that were inactive against the pathogens tested in the present study still permeate LUV membranes (Supplementary Figure S7). These include, for instance, DICAM 13, which lacks the *C*-terminal Glu amino acid and was able to permeate all tested model membranes, or DICAM 17, which carries **Ala** instead of **Pro** as second amino acid and showed a LUV permeabilization pattern similar to those of DICAMs 1 and 4. Also, DICAMs 6 and 7 were particularly effective on DOPE-containing LUVs. However, they showed no antibacterial activity against the Gram-negative pathogens tested (Table 1). Likewise, DICAM 6 did not disrupt the membrane of the *S. pyogenes* ^T^ despite displaying some activity on LUVs composed of POPG:CL:GlcDAG.

Altogether, LUV experiments and *S. pyogenes* membrane-perturbation assays demonstrated that DICAMs can bind, penetrate and perturb lipid membranes without establishing favorable electrostatic interactions with the lipid matrix. Other factors relevant to the differential uptake of DICAMs observed include the feasibility of making hydrophobic and polar interactions with the lipid matrix, the curvature and fluidity of membranes and the formation or not of lipid clusters, which would depend on both the specific lipid composition of target-cell membranes, including the hydrophobic tails, and DICAM’s structure (Domenech et al., 2006; Son and Tew, 2008; Kumar et al., 2018). Besides, the sigmoidal or biphasic nature of permeation kinetics induced by various DICAMs indicated a multi-step mechanism for membrane permeation. Additionally, the contrast between the fast and efficient permeation of *S. pyogenes* membranes by DICAM 19 and its rather slow release of calcein from CL-containing LUVs could denote some additional interactions of this lipo-tripeptide with other components of the bacterial envelope and/or differences in the acidic-chain composition.

### 2.5 Toxicity and hemolysis

The potential toxicities of DICAMs used in dose-dependent activity studies, enumerated in Table 2, were investigated on human erythrocytes (hRBCs) and three cellular lines (human lung carcinoma epithelial cells A549, human fetal lung fibroblasts IMR-90, and human HeLa cervix carcinoma cells) after exposure to DICAMs. Results are summarized in Supplementary Figure S8; Table 3 as HC_50_ and IC_50_ values (concentrations yielding 50% hemolysis or 50% cell growth inhibition, respectively). Most DICAMs tested caused hemolysis at relatively low concentrations with HC_50_ values ranging between 6 μM and 13 μM. Two exceptions were found: DICAM 14 with a HC_50_ of 36 μM and DICAM 19 with a much higher HC_50_ of 120 μM. Notably, the drop in the hemolytic activity shown by DICAM 19 requires both the Glu residue at the *N*-terminus of the peptide and the Cbz-capping and does not depend on hydrophobicity as measured by RP-HPLC (Table 1). The cytotoxicity on the other mammalian cell lines was lower than that on hRBCs, and the IC_50av_ values varied between 36 μM and 86 μM, except for DICAM 14 which was the most cytotoxic (IC_50av_ = 7.3 μM) while HeLa cell line was the most sensitive one. The fatty amine length barely affects the hemolytic activity and cytotoxicity. The selectivity indexes (SI), defined as the ratios of HC_50_ —or the average IC_50_ value— to the respective SC concentration, are shown in Table 3. These data indicated that DICAM 19, with SI values of 12–40, may be a suitable therapeutic option against *S. pyoge*nes and *S. pneumoniae*, followed by DICAM 3 in the former case (SI_*Spy*_ ≥ 3.7), and by DICAMs 1 (SI_*Spn*_ ≥6.2) and 10 (SI_*Spn*_ ≥ 3.2) in the latter (Greber et al., 2017; Neubauer et al., 2020). They also constitute suitable templates for further developing more selective and efficient antibacterials.

Interestingly, the comparatively high HC_50_ value of DICAM 19 does not associate with an equally much higher IC_50_ value on the cell-cultured test, and such differential toxicity could arise from the specific features of the hRBC cellular surface. In this line, hRBCs are richly coated with sialic-rich glycoproteins and hence negatively charged (Jan and Chien, 1973), which may hinder the access and attachment of the twice negatively-charged DICAM 19 to the cell membrane. Besides, as compared to the *S. pneumoniae* or *S. pyogenes* membranes (Panos et al., 1966; van de Rijn and Kessler, 1979; Lu and Rock, 2006), the higher proportion of fatty acids with more than one unsaturation in the erythrocyte membranes may confer them greater fluidity (Owen et al., 1982), increasing the possibility that unbalanced tensions created by DICAM 19 incorporation into the membrane can be at least partially dissipated without causing permeation. On the other hand, it has been found that cholesterol stabilizes lipid membranes and can reduce the activity of certain antimicrobial lipo/peptides (Owen et al., 1982). Specifically, fengicin, a mixture of negatively charged lipid cyclopeptides containing two glutamic acid residues, can be incorporated into cholesterol-rich membranes without disrupting the membrane structure, which has been proposed to be related to the formation of hydrogen bonds between fengicin and cholesterol (Fiedler and Heerklotz, 2015). Interestingly, fengycins also have low hemolytic activity. On the contrary, cholesterol enhanced membrane permeation by surfactin, which is more hemolytic than fengycin (Fiedler and Heerklotz, 2015).

## 3 Discussion

The discovery of new antimicrobial drug candidates from any source is urgent, and antimicrobial lipo/peptides have the potential to become lead structures due to their ability to effectively eradicate pathogenic bacteria and biofilms. Currently, only daptomycin, a natural cyclic lipopeptide, has been clinically approved but there are other compounds under clinical trials or preclinical stages (Koo and Seo, 2019; Kundu, 2020). Novel drug candidates, either designed or evolved, should have improved antimicrobial properties, including selectivity for pathogen membrane permeabilization compared to host cell membrane permeabilization. To keep development costs low, novel lipo/peptides have to be small in size and have a simple structure. In this context, engineered USLPs are the shortest biologically active lipopeptides reported so far. In them, the fatty acyl chain must compensate for the reduction of hydrophobic amino acids within the peptide chain, while the proximity of the charged moiety may offset the oligomerization tendency.

### 3.1 Antibacterial activity and selectivity

Here we report the *de novo* synthesis and functional characterization of DICAMs, a family of membrane-active USLPs with uncommon features. To the best of our knowledge, it comprises the first active USLPs reported so far in which a peptide with a negative or zero net charge is conjugated to a fatty amine chain through the *C*-terminal carboxyl of the peptide. DICAMs may be highly active against selected Gram-positive pathogens under standard culture-growth conditions (SC values in the low micromolar range) and salt concentrations found in the human nasopharynx and airway surface secretions (Moscoso et al., 2006). Based on assayed compounds, microbicidal activity requires: *a*) a tripeptide fussed to a fatty amine with more than 12 carbon atoms, *b*) **Pro** as second amino acid (no substitutable by **Ala**, and *c*) an unsubstituted Glu as third amino acid. Besides, based on DICAMs 4 and 23 activities, d-amino acids may be tolerated at the *N*-terminus of the tripeptide, which can increase their stability against proteases (Lee et al., 2019; Zhong et al., 2020). Specifically, several DICAMs showed a quick and efficient killing of *S. pneumoniae* and *S. pyogenes*, two major Gram-positive human pathogens. *S. pneumoniae* causes over 1.2 million deaths annually (GBD, 2017 Causes of Death Collaborators, 2018) and is on the WHO’s priority list of multidrug-resistant bacteria that require urgent development of novel antimicrobials (Taconelli et al., 2018; Cools et al., 2021). *S. pyogenes* is responsible for a wide variety of diseases ranging from mild localized infections to life-threatening invasive ones (Kanwal and Vaitla, 2023). Every year, it leads to over 500,000 deaths worldwide, as well as post-immune mediated diseases like rheumatic fever, post-streptococcal glomerulonephritis and rheumatic heart disease (World Health Organization, 2022).

The growth and viability of pneumococcal strains were adversely affected by active DICAMs regardless of their virulence or antibiotic susceptibility status. Of notice, the virulent encapsulated variants may be more susceptible to DICAMs than the non-encapsulated ones, according to DICAM 4 activities against *S. pneumoniae* strains. DICAMs may be also active against biofilm-grown cells. The antimicrobial effects were observed against cultures of *S. pneumoniae* and *S. pyogenes* at different stages of growth. However, the activity was reduced and/or slowed down, particularly, when DICAMs were added at the stationary phase of growth, considering results with reference compounds DICAMs 3 and 4. It is noteworthy that CL concentration in *S. pneumoniae* increases as the culture goes from T1 to T3 (Tombe et al., 1979), with the subsequent reduction in PG content. This could explain, among other factors, the observed decrease in DICAM’s activity against this pathogen at T2 and T3.

*S. pneumoniae* was extremely sensitive to DICAMs 2, 4, 12 and 23 (Asn, Ala or **Ala** as first amino acid), followed by DICAMs 19 and 20 (Cbz-Glu or Glu as first amino acid). They all reduced the number of viable cells by 99.2%–99.8% after 1-h incubation at 3 × SC values. In contrast, *S. pyogenes* ^T^ was more susceptible to the *N*-terminal capped compounds of Ala (DICAMs 3 and 24) and Glu (DICAM 19) and the *N*-deprotected Asn derivative (DICAM 2), which reduced the bacterial viability by 94.5%–99.9% after 2 h of incubation at 3 ✕ SC. In both pathogens, the cooperativity observed in the microbicidal effects indicated that multiple molecules of DICAMs must collaborate to induce bacterial death. Based on the SI values, DICAM 19 will be a suitable therapeutic option for both pathogens, followed by DICAM 3 (*S. pyogenes*), and DICAMs 1 and 10 (*S. pneumoniae*) (Greber et al., 2017; Neubauer et al., 2020). Besides, topical use of DICAMs may be relevant for the various epithelial infections (cellulitis, impetigo, erysipelas, necrotizing fasciitis, etc.) caused by *S. pyogenes*. DICAMs 19, 10, 3 and 1 constitute also suitable templates for further developing more selective and efficient antibacterials. Possible approaches to achieve this aim could include *i*) a broader scan of amino acids at the *N*-terminal position, including the use of D enantiomers and other cappings; *ii*) the conjugation of branched or unsaturated fatty amines with similar hydrophobicity or the use of two shorter lipid chains instead of a single long lipid chain (Ghosh et al., 2016; Neubauer et al., 2020); and *iii*) combination of DICAMs with nanoparticles or delivery systems, which could also improve their stability (Kumar et al., 2018; Mulani et al., 2019).

### 3.2 Membrane targeting

Five of the eight most active compounds against *S. pneumoniae* or *S. pyogenes* ^T^ have a negative net charge while the other three are neutral zwitterions, and all contain at least one Glu residue. These results showed that the unfavorable electrostatic interaction with the negatively charged surface of both pathogens does not prevent these DICAMs from binding to, disrupting, and permeating the bacterial membrane. Furthermore, their efficient permeation of LUVs composed of pure POPG and the drastic reduction observed on LUVs containing 80% DOPE strongly indicate a preference for acidic lipids over neutral zwitterionic ones. Likewise, even in the presence of EDTA, *E. coli* and *P. aeruginosa* showed resistance to DICAMs 6, 7, and 13, which have a positive net charge and can efficiently permeate LUVs containing 80% DOPE, but were moderately susceptible to neutral zwitterions (DICAMs 2 and 4) which did not permeate these LUVs. Altogether, these results suggest that active DICAMs prefer acid lipid membranes. Interestingly, this preference is also observed in anionic nonribosomally synthesized cyclic lipopeptides like daptomycin or surfactin, whose interaction with target cell membranes is not primarily driven by electrostatic forces (Fiedler and Heerklotz, 2015; Mescola et al., 2020). Daptomycin (net charge −3), binds specifically to PG-enriched fluid regions in the bacterial membrane of Gram-positive bacteria where it interferes with the synthetic machinery of the cell wall (Grein et al., 2020; Kotsogianni et al., 2021). In contrast, the antibacterial action of surfactin (net charge −2) comprises a complex pattern of effects leading to perturbation or disruption of membrane integrity (Zhen et al., 2023), which was enhanced by POPG and attenuated by POPE (1-palmitoyl-2-oleoyl-sn-glycero-3-phosphoethanolamine) (Fiedler and Heerklotz, 2015; Carrillo et al., 2023). Penetration of surfactin in the lipid bilayer is facilitated by the presence of mono or divalent cations (K^+^, Na^+^, and Ca^2+^) that totally or partially neutralize the two acidic residues (Maget-Dana and Ptak, 1992; 1995), while daptomycin requires Ca^2+^ (Kotsogianni et al., 2021). Besides, a fully active configuration of daptomycin also seems to depend on the hydrogen bonds of glycerol and high contents of CL, while the increment in the proportion of positively-charged Lysyl-PG (1,2-dipalmitoyl-*sn*-glycero-3-[phospho-*rac*-(3-lysyl(1-glycerol))]) prevent its microbicidal effect (Taylor and Palmer, 2016; Gray and Wenzel, 2020; Karas et al., 2020; Kotsogianni et al., 2021). As the size of the polar heads of CL and phosphatidylethanolamine (PE) lipids is smaller than that of the hydrophobic tails, both lipids may induce a negative curvature, leading to a greater packing and stiffening of the bilayer, hampering microbicidal effects (Lewis and McElhaney, 2009).

Our results demonstrate that DICAMs are effective at high salt concentrations (≥0.12 M), as probed by their ability to inhibit bacterial growth, clear bacteria and perturb membranes in standard culture media and salt-containing buffers. However, they do not require Ca^2+^ for activity, as demonstrated by the permeation of LUVs in the presence of EDTA, which also improved DICAM’s activity against Gram-negative bacteria. Hence, it can be speculated that salt counterions might reduce, as in surfactin, the electrostatic repulsion between DICAMs and the bacterial surface by shielding their charges. This would aid the negatively charged DICAMs to insert into acidic lipid bilayers, providing that feasible hydrophobic and polar (hydrogen bonds and/or van der Waals forces) favorable contacts are sufficient to stabilize the interaction. In such a scenario, the energetic cost of segregating charged molecules would be lower than that of uncharged ones (charge-charge repulsion is unavoidable), while the head-to-head distances at the equilibrium would be larger, increasing the positive intrinsic curvature of POPG-containing membranes, and both effects would favor membrane permeation (Fiedler and Heerklotz, 2015). Additionally, the low sensitivity of *S. aureus* ^T^ to most DICAMs tested may be related, among other factors, to the presence of the positively charged lipid Lys-POPG in its cellular membrane, as indicated by the daptomycin results mentioned above (Gray and Wenzel, 2020). On the other hand, the finding that the microbicidal effect of DICAM 4 against the encapsulated D39 variant of *S. pneumoniae* was higher than against the nonencapsulated isogenic strain R6 evidenced that the capsular polysaccharide may contribute to the lethality of DICAMs in Gram-positive pathogens. On the contrary, in Gram-negative bacteria, the outer membrane acts as a barrier, preventing the access of DICAMs to the inner membrane, unless outer membrane permeating agents are added. Interestingly, the possibility of bacterial killing without lysis, as it occurred in *S. pyogenes,* may be advantageous in DICAM use as antimicrobials, by preventing the release of large amounts of pro-inflammatory bacterial components and their harmful physiological consequences (Holzheimer, 2001).

### 3.3 Mechanism of action

Fluorescence assays with SYTOX Green and DiSC3(5) support a membrane perturbation mechanism of killing that can involve the transition from the formation of “transient pores” to “equilibrium pores” as the dose of DICAM increases. The results suggest as well that bacterial membrane perturbation occurs through a series of steps, the timing of which may be compound-dependent, as evidenced by the uncoupling observed between the fluorescence variation kinetics of DiSC3(5) and SYTOX Green when *S. pyogenes* ^T^ was treated with DICAM 3 or DICAM 19. In both cases, depolarization events (permeation to small ions) were detected at DICAM concentrations resulting in complete permeabilization of the *S. pyogenes* ^T^ membrane to SYTOX and leading to over 99% cell death. A partial permeation of *S. aureus* and *S. epidermis* membranes without altering the membrane potential was observed as well at certain concentrations of melittin (Boix-Lemoce et al., 2020), a paradigmatic pore-forming peptide (Ladokhin et al., 1997). This observation has been suggested to be linked to the formation of transient pores that cause limited damage to the membrane, but bacteria may still recover and maintain the proton motive force to some extent.

Domains rich in anionic lipids are often associated with regions where the cell wall synthetic machinery of the bacteria is located (Rosch et al., 2007; Tsui et al., 2011; Pogliano et al., 2012; Müller et al., 2016). They also play an important role in protein translocation across the membrane and in determining the topology of membrane proteins (de Vrije et al., 1988; van Kompenburg et al., 1997; Rosch et al., 2007; Gold et al., 2010; Tsui et al., 2011). Therefore, compounds that preferentially bind to and perturb anionic-enriched lipid regions, may also cause inhibition of cell wall biosynthesis and interfere with protein secretion through the Sec translocase pathway. In line with this, daptomycin preferentially interacts with and rearranges fluid lipid microdomains rich in PG, where the cell wall biosynthesis protein machinery is concentrated (Pogliano et al., 2012; Müller et al., 2016; Grein et al., 2020). This interaction leads to the formation of a complex with PG and a bactoprenyl-coupled lipid intermediate that blocks cell wall biosynthesis and triggers the delocalization of the biosynthetic machinery (Müller et al., 2016; Grein et al., 2020). In *S. pneumoniae*, subunits of the Sec translocase pathway localize dynamically to the equator and septa of dividing cells, coincidental to regions of peptidoglycan biosynthesis and cell division. Localization depends on the anionic phospholipid content and is lost at the stationary phase (Tsui et al., 2011). In *S. pyogenes*, the anionic phospholipids appear to be concentrated at a single region enriched in PG, which overlaps with the membrane microdomain dedicated to the secretion and folding of proteins (ExPortal) (Rosch et al., 2007). Therefore, in addition to permeabilizing the cell membrane, the microbicidal effect of DICAMs against these pathogens could involve additional mechanisms of action. Exploration of this possibility warrants further investigations.

## 4 Conclusion

We have designed, synthesized, and characterized DICAMs, a novel family of membrane-active USLPs with unique features, comprising tripeptides (net charge from −2 to +1) attached to a long fatty amine through the C-terminal carboxyl of the peptide. DICAMs also include at least one D-amino acid to enhance stability against proteases and their potential impact on membrane fluidization (Neubauer et al., 2020). Several DICAMs with strong activity against two major human pathogens — *S. pneumoniae* and *S. pyogenes* — and good-to-tolerable pathogen vs. host cell selectivity have been identified. Differential specificities of DICAMs for each pathogen have also been established. They may kill bacteria grown as planktonic cultures or forming biofilms and completely inhibit bacterial growth at low micromolar concentrations under standard culture-growth conditions and ionic strengths found in fluids from the human airway. Much lower antimicrobial effects were observed against *S. aureus* ^T^, *E. coli* DH10B and *P. aeruginosa* PAO1. Remarkably, the most lethal compounds comprise either an anionic or a neutral zwitterionic tripeptide (**Pro** and Glu as second and third amino acids) attached to a fatty amine of 16/18 carbon atom long. Moreover, they preferentially interact with and disrupt anionic lipid membranes with a high proportion of PG, inducing permeation and depolarization in a dose-dependent way. Thus, attachment of DICAMs to bacterial membranes should be driven by non-electrostatic interactions and salt counterions might work by shielding the negative charges, minimizing unfavorable electrostatic effects. Our results also showed that several molecules of DICAMs may cooperate for their microbicidal effect and that other elements of the bacterial envelope, including the capsular polysaccharide, may modify DICAM’s activity. On the other hand, the possibility that the microbicidal activity of DICAMs may involve other modes of action in addition to membrane permeation seems plausible, considering that PG-rich microdomains and elements of the cell wall biosynthetic machinery colocalize in the cell membrane of susceptible bacteria (de Vrije et al., 1988; van Klompenburg et al., 1997; Rosch et al., 2007; Gold et al., 2010; Tsui et al., 2011; Pogliano et al., 2012; Müller et al., 2016; Grein et al., 2020). In summary, our study provides insight into the features that make DICAMs attractive candidates as antimicrobials and good templates for developing novel compounds with improved bioactivity and toxicity profiles of use in medical or technological settings.

## 5 Materials and Methods

### 5.1 Chemical methods

#### 5.1.1 General methods for lipopeptide synthesis

Unless otherwise noted, analytical grade solvents and commercially available reagents were used without further purification. DIC, PyBOP, and HCTU were purchased from Fluorochem (United Kingdom), TFA from Fluka (Germany) and all other reagents from Aldrich (Germany). Fmoc-protected amino acids were bought from Fluorochem (United Kingdom), Novabiochem (Merck, Germany) and Iris Biotech (Germany). Fmoc-protected Rink Amide MBHA resin (0.56 mmol/g loading) and Wang resin (0.44 mmol/g loading) were purchased from Iris Biotech (Germany). H-Asn- **Pro** -Glu-OMe (DICAM 5), and H-Asn-**Pro**-Tyr(Bzl)-NH-C_18_H_37_ (DICAM 6) were purchased from Peptide Protein Research (United Kingdom). The pure products were analyzed by HPLC and HRMS (High Resolution Mass Spectrometry) (Supplementary Section S4, S5).

DICAMs were synthesized, by two different solid-phase peptide approaches, manually on a 20-position vacuum manifold (Omega) connected to a vacuum pump using 20-mL polypropylene plastic syringes (Dubelcco) with a pre-inserted frit and a Teflon stopcock to do the washings and remove the solvents and excess of the reagents. The SPPS coupling/deprotection reactions were performed over the previously swollen adequate resin inside of the syringes by adding the corresponding reagents, and subsequent automatic stirring on a Grant-Bio (POS-300) orbital shaker. After cleavage, the acidic crudes were sedimented in Et_2_O on a Hettlich Universal 320R centrifuge at 5,000 rpm. All the crude and samples were lyophilized using water/acetonitrile mixtures on a Telstar 6–80 instrument. Monitoring of the reactions and final compounds was performed by using the colorimetric ninhydrin (Kaiser) test for primary amines, the chloranil test for secondary amines, or by HPLC/MS on an HPLC-Waters 12,695 connected to a Waters Micromass ZQ spectrometer.

DICAMs were purified by HPFC (High Performance Flash Chromatography) on an SP1 Isolera Biotage instrument using two different methods. Method A: N-terminal Cbz-peptides were purified on a cartridge (Sfär Silica-HC-D 10 g). As mobile phase, a mixture of A:B, was used, where A = DCM and B = 20% MeOH in DCM, with a flow rate of 7 mL/min, using a gradient from 0% of B to 100% of B in 30–45 min and they were detected at 217 nm. Method B: H-peptides were purified using reverse-phase columns (KP-C18-HS 12 g). As mobile phase, mixtures of A:B were used, where A = 0.05% TFA in water and B = acetonitrile, with a flow rate of 7 mL/min, using a gradient from 0% of B to 100% of B in 30–45 min and were detected at 217 nm. After purification, the target compounds were lyophilized and dried, under reduced pressure, in the presence of P_2_O_5_. For the *N*-deprotected DICAMs, the lyophilization was carried out in the presence of HCl to afford the desired peptides as hydrochloride salts.

The purity of the lipopeptides was checked by analytical RP-HPLC on an Agilent Infinity instrument equipped with a Diode Array and a C18 Sunfire column (4.6 mm × 150 mm, 3.5 μm). As mobile phase A:B mixtures were used, where A = 0.05% TFA in water and B = acetonitrile. The samples were analyzed at 217 and 254 nm in a gradient from 10% of B to 100% of B in 20 min followed by isocratic 100% of B in 10 min.

Retention times were used to determine the hydrophobicity (Neubauer et al., 2020). HRMS (EI+) was carried out in an Agilent 6,520 Accurate-Mass Q-TOF LC/MS spectrometer using water/acetonitrile mixtures. Results can be found in Supplementary Section S3, S4.

#### 5.1.2 General procedure A for the synthesis of C-terminal Glu-containing lipopeptides

The peptides were synthesized by standard SPPS on a Wang resin (0.44 mmol/g loading) using *N*-Fmoc or *N*-Cbz protected natural or unnatural amino acids (R groups) and orthogonal protection strategies for the side-chains of the different residues or the terminal COOH of the Glu amino acid when introduced at the *N*-terminus. Thus, suitable protected amino acids Fmoc-Glu(OH)-OAll, R-Asn(Trt)-OH, R-Orn(Boc)-OH and R-Glu(O^*t*^Bu)-OH were used. Coupling reactions were performed in the presence of DIC or PyBOP and DMAP or DIEA as coupling reagents and bases. For detailed descriptions of the various steps, please refer to the Supplementary Section S1.1.

##### 5.1.2.1 Application of procedure A to the synthesis of DICAM 1

Starting from 0.114 mmol of resin and using Cbz-Asn(Trt)-OH as *N*-terminal residue, the *N*-protected lipopeptide DICAM 83 (Cbz-Asn-**Pro**-Glu-NH-C_18_H_37_) was purified and isolated as a white lyophilized cotton-like solid (42 mg, 50% overall yield).

HPLC: 21.41 min (99% analytical purity). HRMS (ESI,+) m/z: calculated for C_40_H_65_N_5_O_8_ 743.4833; found 744.4853 (2.65 ppm). Results can be seen in Supplementary Sections S3, S4.

The syntheses of the other DICAMs produced by this procedure are detailed in Supplementary Section S1.1.1.

#### 5.1.3 General procedure B for the synthesis of *C*-terminal non-containing Glu lipopeptides

Lipopeptides were synthesized manually on a Rink amide MBHA resin (0.56 mmol/g loading). Fmoc-group was deprotected according to the general method described above. First, Fmoc-Ser(Trt)-OH (1.25 eq) was coupled to the solid support using HCTU (1.25 eq) and DIEA (2.50 eq) in DMF at room temperature for 2 h. Similarly, elongation of the peptidyl resin was performed using the HCTU/DIEA coupling system.

##### 5.1.3.1 On-resin activation of Ser residue to cyclic urethane moiety

To the corresponding Fmoc-tetrapeptidyl resin (1 eq), containing a Ser as *C*-terminal residue, a solution of DSC (15 eq), DIEA (15 eq) and a catalytic amount of DMAP in DMF was added. The activated cyclic urethane peptide resin inside of the syringes was left on a shaker at room temperature overnight. The solution was drained and the resin was washed with DMF to be used in the following step.

##### 5.1.3.2 Synthesis of peptide-fatty amine conjugate from solid support

To the corresponding activated peptide as cyclic urethane on the resin (0.114 mmol), stearyl amine (89 mg, 2.3 eq,) in dry THF (1 mL) was added and the resin was heated at 60°C for 4 h or overnight. The resin was filtered and the solvent was concentrated under reduced pressure. The residue was dissolved in DCM (20 mL) and was washed with 1 N HCl solution. The organic phase was dried (Na_2_SO_4_), filtered, and evaporated to dryness. The deprotection of resulting Cbz-lipopeptide was carried by hydrogenolysis using CH_2_Cl_2_/MeOH 1:1 (6 mL) as a solvent and 10% Pd/C (40 wt %) as a catalyst, in the presence of H_2_ at 30 psi at 30°C for 2 h. The final crude residue was purified on an SP1 Isolera Biotage, lyophilized in the presence of HCl and dried under P_2_O_5_ to give the lipopeptides as hydrochloride salts.

##### 5.1.3.3 Application of procedure B to the synthesis of DICAM 7

Starting from 0.114 mmol of resin using Cbz-Asn(Trt)-OH as *N*-terminal residue, and Fmoc-Phe-OH as *C*-terminal residue, *N*-deprotected DICAM 76 (H-Asn- **Pro** -Phe-NH-C_18_H_37_ ·HCl) was obtained as a white lyophilized cotton-like solid (40 mg, 53% overall yield).

HPLC: 22.21 min (97% analytical purity). HRMS (ESI,+) m/z: calculated for C_36_H_61_N_5_O_4_ 627.4712; found 627.4724 (1.86 ppm). Results can be found in Supplementary Sections S3, S4.

The syntheses of the other DICAMs obtained by this procedure are detailed in section Supplementary Sections S1.1.2.

### 5.2 Biological methods

#### 5.2.1 Products

POPG, DOPE, GlcDAG and beef heart CL were from Avanti Polar Lipids (G.B.). SYTOX Green, DiSC3(5) and the LIVE/DEAD BacLight Bacterial Viability Kit were from Invitrogen (Fisher Scientific, Spain). Calcein was from Sigma-Aldrich. Human whole blood K2EDTA-preserved was from NeoBiotech (France) and defibrinated sheep blood from Oxoid (Thermo Fisher Scientific, Spain). All other chemicals were from Sigma-Aldrich unless otherwise stated.

#### 5.2.2 Microorganisms

Bacterial strains used in this work (Supplementary Table S2) were stored at −80°C in glycerol 15% (v/v). Gram-positive bacteria were grown at 37°C without aeration in Brain Heart Infusion (BHI) except *S. pneumoniae*, grown in C medium supplemented with 0.08% (w/v) yeast extract (C + Y medium) (Lacks and Hotchkiss, 1960). Gram-negative bacteria were grown at 37°C with shaking at 200 rpm in Luria-Bertani (LB). Solid culture was carried out either on LB agar plates for Gram-negative strains or trypticase soy agar (TSA) plates containing 5% (v/v) defibrinated sheep blood for Gram-positives unless otherwise stated.

#### 5.2.3 Growth inhibition and bactericidal activity

##### 5.2.3.1 Planktonic cultures

Susceptibility to DICAMs was first tested by growth-inhibition assays at 50 and 100 μM of compound. Briefly, working solutions at 5 mM or 10 mM DICAM in pure DMSO were prepared from DICAM stocks (40 mM in pure DMSO) and diluted with fresh culture medium to 5× the desired final concentration. Aliquots (50 µL) of these solutions were added to 200 µL of bacterium cultures grown to early exponential phase (OD_550/600_ *c. a*. 0.15–0.2 in sterile 96-well plates (Cultek, Spain). Incubation continued for 10 h at 37°C and turbidity (OD_550/600_) values were measured every 20 min to monitor growth and lysis, using a VersaMax microplate reader (Molecular Devices; United Kingdom). Next, the activity dependence on DICAM’s dose was examined by measuring the turbidity of treated and untreated cultures as a function of time. Also, the decay of bacterial viability (CFU/mL) at selected times of treatment was assessed by colony counting on LB-agar or TSA plates after overnight incubation at 37°C. By default, 10 μL of 10-fold serially diluted samples were plated, which set the lower detection limit at 10^2^ CFU/mL (10^1^ CFU/mL when 100 μL of the undiluted sample was seeded).

As antibacterial efficacy may decline with the bacterium dose in the inoculum (Belley et al., 2009; Udekwu et al., 2009; Luca et al., 2013), activity assays were performed using the bacterial concentrations required for the fluorescence experiments in Section 2.3 (10^7^–10^8^ CFU/mL). At such bacterial densities, MICs were determined as the DICAM concentration at which bacteria neither grow nor are killed under assay conditions (the stationary concentration (SC)), as indicated in Section 2.2. The relationship between variation of bacterium population in logarithmic units, Δlog, and the DICAM concentration, [D], was described as a Hill function:

$$\Delta \log=\Delta \log_{0}+\left(\Delta \log_{f}-\Delta \log_{0}\right){\left[D\right]}^{N_{H}}/\left(K_{1/2}^{N_{H}}+{\left[D\right]}^{N_{H}}\right)$$

where ∆log_*f*_ and ∆log_*0*_ are the upper and lower limits of Δlog, *K*_1/2_ is the DICAM concentration producing half variation in Δlog_max_ = (∆log_*f*_ - ∆log_*0*_), and *N*_H_ is the slope of the dose-response curve (Hill coefficient). The values of Δlog_max_, *K*_1/2_, and *N*_H_ were estimated from the data fitting, along with the SC value (DICAM concentration at which Δlog = 0). Blanks without bacteria (negative controls) and cultures grown in the absence of DICAMs (positive controls) were always run in parallel. No interference in control growth or cell viability was observed in the presence of 1% DMSO (the highest concentration in the assays, contributed by DICAM’s vehicle solution).

The effect of DICAMs at different phases of culture growth was also investigated. DICAMs (50 μM) were added to cultures in the early exponential phase (OD_550_ ≈ 0.15; T1), late exponential phase (OD_550_ ≈ 0.3–0.4; T2), or near the stationary phase of growth (OD_550_ ≈ 0.5–0.7; T3) and the evolution of OD values was followed at 37°C for at least 2 h. By default, the variation in cell viability was also determined. Negative and positive controls were run in parallel.

Phase contrast and fluorescence microscopies were also used to examine the effect of DICAMs on bacterial cultures. The viability of bacterial populations was assessed using the LIVE/DEAD BacLight Bacterial Viability Kit to stain cells (10 min at room temperature in the dark; 1:1 mixture of SYTO 9 and propidium iodide), following the manufacturer’s instructions. Bacteria were then observed at ×100 magnification using a Leica DM400B fluorescence microscope (Leica Microsystems; Spain). SYTO 9 penetrates all bacteria and stains them green, while propidium iodide penetrates only those with a damaged membrane, staining them red.

##### 5.2.3.2 Resting cells

For activity assays on resting cells, bacteria grown to exponential phase (OD_600_ ≈ 0.3) were centrifuged and washed with 20 mM Tris-HCl (pH 7.5), adjusting the final OD_600_ to ≈0.6 (≈10^8^ CFU/mL) with the same buffer. If required, EDTA (0.5 mM final concentration) was next added and the bacterial suspension was dispensed (225 µL per well) in 96-well plates, and then treated with 100 µM DICAM (25 µL). Samples were incubated at 37°C for 3 h and turbidity measurements were taken at selected intervals, also measuring the decay in cell viability at the end of incubation. Controls with buffer (with and without 1% DMSO), 100 µM DICAM, or 0.5 mM EDTA were run in parallel.

##### 5.2.3.3 Biofilm assays

Biofilms were prepared as described (Domenech and García, 2020). Briefly, *S. pneumoniae* R6 cells in late exponential growth (OD_550_ ≈ 0.6; C + Y medium) were pelleted by centrifugation, resuspended in an equal volume of fresh medium, diluted 1/100 (4.5 × 10^6^ CFU/mL), and then dispensed (200 µL per well) in 96-well flat-bottom polystyrene plates (Costar 3,595; Corning; Spain). After plate incubation at 34°C for 4.5 h without shaking, bacterial growth (non-adherent cells plus biofilm-forming cells) was determined by measuring the OD_550_ using a VersaMax microplate absorbance reader. The supernatant (non-adherent cells) was replaced by 200 μL of DICAM 4 (150, 300 or 600 µM) prepared by dilution in sterile water and plate incubation was continued for 1 h at 37°C. Plates were then washed and the biofilms disaggregated in sterile water for viable cell determination in Mueller-Hinton blood agar plates after overnight incubation at 37°C. For the observation of biofilms by CLSM, bacteria were grown on glass-bottomed dishes (WillCo-dish; WillCo Wells, Netherlands) and treated as above, plating 2 mL of the final inoculum per disc and then adding 0.5 mL of DICAM solution. After removing non-adherent cells, biofilms were stained (10 min at room temperature in the dark) with the LIVE/DEAD BacLight Bacterial Viability Kit and then observed using a Leica TCS-SP2-AOBS-UV CLSM (Leica Microsystems, Spain) at ×63 magnification (zoom 2). Laser lines at 488 nm (SYTO 9 excitation) and 561 nm (propidium iodide excitation) were provided by an argon laser and a DiodeP solid state laser, respectively. Detection ranges were set to eliminate cross-talk between fluorophores. The image resolution was 8 bits and the format was 512 × 512 pixels. Laser intensity and gain were kept the same for all images and they were analyzed using LCS software from Leica. Maximum intensity projections were obtained in the *x*–*y* (individual scans at 0.5–1 μm intervals) and *x*–*z* (images at 5–6 μm intervals) planes. Orthogonal projections were also obtained. All the experiments were performed in triplicate and controls adding either water or the DICAM vehicle solution were run in parallel.

#### 5.2.4 Permeation and depolarization assays

Permeation and depolarization of *S. pyogenes* ^T^ membrane by DICAMs were assayed with the fluorescent probes SYTOX Green and DiSC3(5), respectively, in a Varioskan Flash microplate reader (Thermo Scientific; Spain) using FluoroNunc 96-well plates (Thermo Scientific; Spain). For permeation assays, cells were grown to an OD_550_ ≈ 0.3, washed once with PBS pH 7.2 (3,800 × *g*, 7 min, room temperature), and adjusted to a final OD_550_ ≈ 0.4 (∼10^8^ CFU/mL) with the same buffer before mixing in the dark with SYTOX Green (5 µM final concentration). One hundred µL of this suspension was dispensed per plate well and the signal stabilization was monitored for 15 min (λ_ex_ = 485 nm; λ_em_ = 520 nm). Then, 100 µL of two-fold serially diluted lipopeptide samples was added and changes in fluorescence intensity were registered for 60 min at 37°C. SYTOX Green (900 Da) binds to intracellular DNA when the cell membrane is damaged, thereby increasing fluorescence intensity (Roth et al., 1997). Positive controls (100% membrane permeabilization) were carried out by adding Titron X-100 (0.01% final concentration) to untreated bacterial samples (Rathinakumar et al., 2009). For membrane depolarization assays, 180 µL of an OD_550_ ≈ 0.2 culture (BHI; ≈ 10^7^ CFU/mL) supplemented with DiSC3(5) (1 µM final concentration) was distributed per well, and signal stabilization was registered for 30 min (λ_ex_ = 622 nm; λ_em_ = 670 nm) (Te Winkel et al., 2016). Subsequently, 20 µL of a two-fold serial dilution of lipopeptide in culture medium was added, and fluorescence intensity was recorded for 60 min at 37°C. Partition of DiSC3(5) to the surface of polarized cells decreases the probe fluorescence, and its release, caused by membrane depolarization, results in an increase in fluorescence. In both assays, controls with either PBS or BHI (with and without the maximum amount of DMSO introduced in the assays by DICAMs and dyes) were run in parallel. The background signal of controls with bacterial cells without the fluorescent probe, as well as the dye fluorescence in the presence of the tested compounds without bacteria, were also registered. No change in the fluorescence intensity of the probes occurred upon DICAM addition in the absence of bacterial cells.

#### 5.2.5 LUV permeation

LUVs were prepared for calceine liberation assays as described (Chongsiriwatana and Barron, 2009). Lipid composition (mole %) of LUVs was: POPG (100), POPG:CL (80:20), POPG:CL:DOPE (12:5:80), and POPG:CL:GlcDAG (75:5:20). Briefly, the nitrogen-dried lipid mixture was dissolved at 1.25 mM with 100 mM calceine in vesicle buffer (10 mM Tris, 140 mM NaCl, 0.5 mM EDTA, pH 7.4) followed by five thaw-freeze cycles. Calceine-entrapped LUVs were prepared by extrusion through 100 nm polycarbonate membranes (Avanti Polar Lipids). Non-entrapped calceine was separated from LUVs by gel filtration on a Sephacryl^®^ S200-HR column (Sigma). LUVs permeabilization was monitored at 25°C by fluorescence emission at 550 nm (λex = 480 nm) as a function of time in a Fluoromax-4 spectrofluorometer (Horiba, France) using agitation. Signal stabilization of samples (1,500 µL vesicle buffer plus 45 µL LUVs) was recorded for 5 min before 10 µM final DICAM addition, and calcein release was monitored for a maximum of 20 min. After incubation, samples were solubilized in Triton X-100 (0.025%) and the fluorescence signal recorded thereafter was used to normalize the entire data set. Solubilization of DICAM-untreated LUVs in Triton X-100 was used as a positive control. No perturbation was detected in controls containing DMSO.

#### 5.2.6 Hemolytic activity

The hemolytic activities of DICAMs were determined using hRBCs as described (Neubauer et al., 2020). Pooled gender human whole blood K2EDTA-preserved was fractioned and hRBCs were washed 4 times with PBS pH 7.4 (1,200 × *g; *10 min; 12°C) and resuspended in the same buffer (2% v/v). DICAMs were two-fold serially diluted (200–0.8 µM range) in PBS and 100 µL of each dilution was mixed with 100 µL of the hRBCs suspension in 96-well conical bottom plates (Cultek; Spain). After 1 h incubation at 37°C with agitation, the plates were centrifuged (800 × *g*; 5 min; room temperature) and 150 µL of the supernatant was transferred to a 96-well flat bottom plate to measure colorimetrically the hemoglobin released at 540 nm (OD_540_) with the reference filter at 620 nm. Negative controls (no hemolysis) with PBS (pH 7.4) and positive controls (100% hemolysis) with 0.5% (v/v) Triton X-100 were run in parallel. DMSO did not induce hemolysis at the concentration added by DICAM’s vehicle solution. The hemolysis percentage was estimated as ((A-A_0_)/(A_max_-A_0_)) × 100 where A, A_0,_ and A_max_ (≈0.7 OD) are the OD_540_ values of the sample, negative control, and positive control, respectively. The HC_50_ was calculated by interpolation from the hemolysis percentage vs. DICAM concentration plots. NeoBiotech is committed to sourcing human and animal biological material following the highest ethical and regulatory standards.

#### 5.2.7 Cytotoxicity assays

DICAM’s cytotoxicity was determined employing a modified MTT assay as described elsewhere (Antúnez-Mojica et al., 2016) using human lung carcinoma epithelial cells A549, human fetal lung fibroblasts IMR-90, and human HeLa cervix carcinoma cells treated with increasing concentrations of DICAMs (0.8–200 µM range). A549 cells were grown in RPMI 1640 (Gibco; Fisher Scientific, Spain) supplemented with 10% FCS, 2 mM glutamine, 100 IU/mL penicillin and 100 μg/mL streptomycin. IMR-90 and HeLa cells were cultured in DMEM (Gibco; Fisher Scientific, Spain) supplemented with 10% FCS, 2 mM L-glutamine, 1 mM pyruvate, 100 IU/L penicillin, 100 μg/mL streptomycin and 40 μg/mL gentamycin. Cell viability was assessed after 48 h exposure to DICAMs. Vinblastine was used as a positive control. IC_50_ value is the concentration that gives 50% inhibition of cell growth and was determined using SigmaPlot (Systat) fitting to a Four Parameter Logistic curve.

#### 5.2.8 Statistical analysis

Statistical analyses were performed using GraphPad Instat v. 6.0 (GraphPad Software, San Diego, USA) and are detailed in figure legends. In all cases, a *p*-value ≤0.05 was considered statistically significant.

## Data Availability

The original contributions presented in the study are included in the article/Supplementary Material, further inquiries can be directed to the corresponding authors.

## Abbreviations

CL, Cardiolipin (heart, bovine cardiolipin; 90% 1ʼ-[1,2-dilinoleoyl-*sn*-glycero-3-phospho]-3ʼ-[1,2-dilinoleoyl-*sn*-glycero-3-phospho]-glycerol); CLSM, Confocal laser scanning microscopy; DCM, Dichloromethane; DIC, *N,N′*-diisopropylcarbodiimide; DIEA, *N,N′*-diisopropyldiethylamine; DMAP, 4-dimethylaminopyridine; DMF, dimethylformamide; DOPE, 1,2-dioleoyl-sn-glycero-3-phosphoethanolamine; GlcDAG, 1,2-diacyl-3-O-(α-D-glucopyranosyl)-sn-glycerol; HC_50_, concentration required to produce 50% hemolysis; HCTU, *O*-(1*H*-6-Chlorobenzotriazole-1-yl)-1,1,3,3-tetramethyluronium hexafluorophosphate; hRBCs, human erythrocytes; K2EDTA, potassium ethylenediaminetetraacetic acid, used to prevent blood clotting; IC_50_, concentration that gives 50% inhibition of cell growth; IC_50av_, IC_50_ averaged over all cell lines; LUVs, large unilamellar vesicles; Lysyl-PG, 1,2-dipalmitoyl-*sn*-glycero-3-[phospho-*rac*-(3-lysyl(1-glycerol))]; MIC, Minimal inhibitory concentration; PE, phosphatidylethanolamine; PG, phosphatidylglycerol; POPE, 1-palmitoyl-2-oleoyl-sn-glycero-3-phosphoethanolamine; POPG, 1-palmitoyl-2-oleoyl-sn-glycero-3-phospho-(1′-rac-glycerol); PyBOP, benzotriazol-1-yl-oxytripyrrolidinophosphonium hexafluorophosphate; SAR, structure-activity relationship; SC, Stationary concentration (defined as the concentration of DICAM that prevents both bacterial death and growth); SI, selectivity index, defined as HC_50_/SC or IC_50av_/SC; SPPS, solid phase synthesis; TFA, Trufluoroacetic acid; TIS, triisopropylsilane; USLP, ultra-short lipopeptide.
