# Supplementary material for: Design, synthesis and structure-activity relationship (SAR) studies of an unusual class of non-cationic fatty amine-tripeptide conjugates as novel synthetic antimicrobial agents
Source: Front Pharmacol. 2024 Aug 2;15:1428409. doi: 10.3389/fphar.2024.1428409 (PMC11329928; doi:10.3389/fphar.2024.1428409)
Supplement: Supplementary file 1 [file DataSheet1.pdf]

Supplementary Material

S1 Supplementary Materials and Methods

S1.1 Synthesis of fatty amine-tripeptide conjugates

S1.1.1 *General procedure A for the synthesis of C-terminal Glu-containing lipopeptides*

The peptides were synthesized by standard SPPS on a Wang resin (0.44 mmol/g loading) using *N*-Fmoc or *N*-Cbz protected natural or unnatural amino acids (R groups) and orthogonal protection strategies for the side-chains of the different residues or the terminal COOH of the Glu amino acid when introduced at the *N*-terminus. Thus, suitable protected amino acids Fmoc-Glu(OH)-OAll, R-Asn(Trt)-OH, R-Orn(Boc)-OH and R-Glu(O^{t}Bu)-OH were used. Coupling reactions were performed in the presence of DIC or PyBOP and DMAP or DIEA as coupling reagents and bases.

S1.1.1.1 *Anchoring of Fmoc-Glu-OAll to the resin*

Wang resin (1 eq, 0.44 mmol/g) was placed in a dried 60 mL filtration tube with polyethylene frit and swelled with DCM (5 × 30 s). In parallel Fmoc-Glu(OH)-OAll (10 eq) was dissolved in dry DCM (3 mL/mmol of resin) and cooled in an ice bath under argon atmosphere. DIC (5 eq) was added dropwise and the reaction mixture was stirred for 10 min. The solvent was eliminated under reduced pressure. The residue was dissolved in DMF (5 mL/mmol of resin) and added to the resin. DMAP (0.1 eq) was added and the reaction mixture was shaken at room temperature for 2 h. The resin was then washed with DMF (5 × 30 s). A mixture of acetic anhydride (2 eq) and pyridine (2 eq) in DMF (5 mL/mmol of resin) was added to the resin and the reaction mixture was shaken at room temperature for 30 min. Finally, the resin was washed twice with DMF (4 × 30 s) and DCM (4 × 30 s).

S1.1.1.2 *Selective deprotection of the allyl ester group (OAll)*

To the previous *C*-terminal protected Glu-peptidyl resin pre-swelled in anhydrous DCM in a syringe (5 × 30 s), $Pd(PPh_3)_4$ (0.25 eq) and $PhSiH_3$ (24 eq) were added in anhydrous DCM (3 mL of solvent per mmol of peptidyl-resin). The syringe was sealed and gently bubbled with argon. Then, the reaction mixture was stirred at room temperature for 1 h. Next, the supernatant was removed and new coupling mixture was added, incubating for 1 h (2 × 1 h in total). The peptidyl-resin was then filtered and washed successively with DCM/DMF/0.2 M Et_2NCS_2Na in DMF/DCM to remove residual palladium impurities.

S1.1.1.3 *General coupling procedure of the fatty amine*

To the deprotected *C*-terminal pre-swelled peptidyl-resin (1 eq), the corresponding fatty amine (4 eq), PyBOP (1 eq) and DIEA (2 eq) dissolved in DMF were added. The reaction mixture was shaken at room temperature overnight. After that, the peptidyl-resin was washed with DMF/DCM/DMF/DCM. The reaction progress was monitored by HPLC-MS.

S1.1.1.4 *General Fmoc deprotection procedure*

To a Fmoc-protected pre-swelled resin a solution of 20% piperidine in DMF was added and the reaction mixture was shacked at room temperature for 1 × 1 min and 3 × 10 min. Then, the *N*-deprotected peptidyl-resin was washed with DMF/DCM/DMF/DCM 4 ×30 s.

S1.1.1.5 *Elongation of the peptidic sequence*

To a pre-swelled peptidyl-resin in a pre-fritted vessel (1 eq) Fmoc-aa-OH (2 eq), PyBOP (2 eq) and DIEA (4 eq) dissolved in DMF were added. The reaction mixture was shaken at room temperature for 2 h. The peptidyl-resin was washed with DMF/DCM/DMF/DCM (4 × 30 s) and the reaction progress was followed by the colorimetric ninhydrin test or by HPLC-MS.

S1.1.1.6 *General cleavage procedure*

The dried lipopeptidyl-resin derivative (1 volume) in a fritted syringe was treated with TFA:TIPS:H_2O 95:2.5:2.5 (5 volumes) at room temperature for 4 h. The filtrates were precipitated over cold Et_2O and centrifuged three times at 5,000 rpm for 10 min. After removing the supernatant, the pellets were redissolved in water/acetonitrile and lyophilized. The crudes were purified on an SP1 Isolera Biotage, lyophilized and dried under P_2O_5 to give the target peptides in high purity.

DICAM 2 (H-Asn- **Pro**-Glu-NH-$C_{18}H_{37}$·HCl). The general procedure A using Fmoc-Asn(Trt)-OH as the *N*-terminal residue and 0.114 mmol of resin was followed. After standard Fmoc deprotection of the final lipopeptidyl resin. The resulting compound was purified, lyophilized and dried in the presence of HCl to produce the hydrochloride salt of DICAM 2 as a white cotton-like solid (31 mg, 42 % overall yield). HPLC: 12.14 min (99% analytical purity). HRMS (ESI,+) m/z: calculated for $C_{32}H_{59}N_5O_6$ 609.4465; found 609.4470 (1.4 ppm).

DICAM 3 (Cbz-Ala-**Pro**-Glu-NH-$C_{18}H_{37}$). Starting from 0.114 mmol of resin, and after following the general procedure A using Cbz-Ala-OH as *N*-terminal residue, the *N*-protected DICAM 3 was isolated, after purification and lyophilization as a white cotton-like solid (80 mg, 68% overall yield). HPLC: 21.42 min (99 % analytical purity). HRMS (ESI,+) m/z: calculated for $C_{39}H_{64}N_4O_7$ 700.4775; found 701.4765 (1.4 ppm).

DICAM 4 (H-Ala-**Pro**-Glu-NH-$C_{18}H_{37}$·HCl). The general procedure A using Fmoc-Ala-OH as *N*-terminal residue and 0.114 mmol of resin was followed. After standard Fmoc deprotection of the final lipopeptidyl resin, the compound was purified and lyophilized in the presence of HCl and dried to afford the hydrochloride salt of the *N*-deprotected DICAM 4 as a white cotton-like solid (69 mg, 40 % overall yield). HPLC: 19.24 min (98% analytical purity). HRMS (ESI,+) m/z: calculated for $C_{31}H_{58}N_4O_5$ 566.5393; found 566.4407 (2.59 ppm).

DICAM 8 (Cbz-Asn-**Pro**-Glu(OtBu)-NH-$C_{18}H_{37}$). The peptide was synthesized starting from 0.114 mmol of resin following the general procedure A and using Cbz-Asn(Trt)-OH as *N*-terminal residue. Then, *tert* butyl group was introduced under solution synthesis conditions. Thus, to a solution of the peptide in DCM (5 mL), H_2SO_4 (15 µL) and 2-methylpropene (2 eq, 13 mg) were added. The reaction mixture was stirred overnight at room temperature. The *N*-protected lipopeptide was purified and lyophilized to give DICAM 8 as a white cotton-like solid (91 mg, 61% yield). HPLC: 4.08 min (99% analytical purity). HRMS (ESI,+) m/z: calculated for $C_{44}H_{73}N_5O_8$ 799.5459; found 799.5464 (0.64 ppm).

DICAM 9 (Cbz-Asn-**Pro**-Glu-NH-$C_{12}H_{25}$). Following general procedure A with 0.114 mmol of resin and Cbz-Asn(Trt)-OH as *N*-terminal residue, the *N*-protected DICAM 9 was obtained as a white lyophilized cotton-like solid (59 mg, 79 % overall yield) after purification. HPLC: 15.80 min (99 %

analyt9ical purity). HRMS (ESI,+) m/z: calculated for $C_{34}H_{53}N_5O_8$ 659.3887; found 659.3894 (1.02 ppm).

DICAM 10 (Cbz-Asn-**<u>Pro</u>**-Glu-NH-$C_{16}H_{33}$). Starting from 0.114 mmol of resin, by following the general procedure A using Cbz-Asn(Trt)-OH as *N*-terminal residue, the *N*-protected DICAM 10 was purified and lyophilized to yield 60 mg (73% overall yield) as a white cotton-like solid. HPLC: 18.23 min (99% analytical purity). HRMS (ESI,+) m/z: calculated for $C_{38}H_{61}N_5O_8$ 715.4520; found 701.4526 (1.09 ppm).

DICAM 11 (H-Ala-**<u>Pro</u>**-Glu-NH-$C_{12}H_{25}$ ·HCl). Starting from 0.114 mmol of resin, and after following the general procedure A using Fmoc-Ala-OH as *N*-terminal residue, the *N*-deprotected DICAM 11 was isolated, after purification, as a white lyophilized cotton-like solid (13 mg, 22% overall yield). HPLC: 15.29 min (98% analytical purity). HRMS (ESI,+) m/z: calculated for $C_{25}H_{46}N_4O_5$ 482.3450; found 482.3468 (3.79 ppm).

DICAM 12 (H-Ala- **<u>Pro</u>**-Glu-NH-$C_{16}H_{33}$ ·HCl). From 0.114 mmol of resin, and Fmoc-Ala-OH as *N*-terminal residue, the N-deprotected DICAM 12 was purified and lyophilized to give a white cotton-like solid (22 mg, 34% overall yield). HPLC: 17.57 min (99% analytical purity). HRMS (ESI,+) m/z: calculated for $C_{29}H_{54}N_4O_5$ 538.4079; found 538.4094 (2.74 ppm).

DICAM 17 (H-Asn-**<u>Ala</u>**-Glu-NH-$C_{18}H_{37}$ ·HCl). From 0.114 mmol of resin, and Fmoc-Asn(Trt)-OH as *N*-terminal residue, DICAM 17 was purified and isolated as a white lyophilized cotton-like solid (30 mg, 42 % overall yield). HPLC: 18.63 min (99% analytical purity). HRMS (ESI,+) m/z: calculated for $C_{30}H_{57}N_5O_6$ 583.4300; found 583.4309 (1.54 ppm).

DICAM 18 (H- **<u>Pro</u>**-Glu-NH-$C_{18}H_{37}$·HCl). Starting from 0.114 mmol of resin, and following the general procedure A using Fmoc-(D)-Pro-OH as *N*-terminal residue, the *N*-deprotected DICAM 18 was purified and isolated as a white lyophilized cotton-like solid (29 mg, 47% overall yield). HPLC: 19.54 min (99% analytical purity). HRMS (ESI,+) m/z: calculated for $C_{28}H_{53}N_3O_4$ 495.4015; found 495.4036 (3.28 ppm).

DICAM 19 (Cbz-Glu-**<u>Pro</u>**-Glu-NH-$C_{18}H_{37}$). Following general procedure A with 0.114 mmol of resin and Cbz-Glu(tBu)-OH as *N*-terminal residue, the *N*-protected DICAM 19 was obtained as a white lyophilized cotton-like solid (53 mg, 61% yield) after purification. HPLC: 21.22 min (99% analytical purity). HRMS (ESI,+) m/z: calculated for $C_{41}H_{66}N_4O_9$ 758.4825; found 758.4830 (0.64 ppm).

DICAM 20 (H-Glu-**<u>Pro</u>**-Glu-NH-$C_{18}H_{37}$ ·HCl). The general procedure A was followed with 0.114 mmol of resin, and after using Fmoc-Glu(tBu)-OH as *N*-terminal residue. The *N*-deprotected DICAM 20 was purified, lyophilized and isolated as a white cotton-like solid (39 mg, 51% yield). HPLC: 18.71 min (97% analytical purity). HRMS (ESI,+) m/z: calculated for $C_{33}H_{60}N_4O_7$ 624.4449; found 624.4462 (2.09 ppm).

DICAM 21 (Cbz-Orn-**<u>Pro</u>**-Glu-NH-$C_{18}H_{37}$). Starting from 0.114 mmol of resin, and after following the general procedure A using Cbz-Orn(Boc)-OH as *N*-terminal residue, the *N*-protected DICAM 21 was purified and isolated as a white lyophilized cotton-like solid (37 mg, 43% overall yield). HPLC: 19.70 min (98% analytical purity). HRMS (ESI,+) m/z: calculated for $C_{41}H_{69}N_5O_7$ 743.5177; found 743.5197 (2.68 ppm).

DICAM 22 (H-Orn-**<u>Pro</u>**-Glu-NH-$C_{18}H_{37}$ ·HCl). From 0.114 mmol of resin, and Fmoc-Orn(Boc)-OH as N-terminal residue, N-deprotected DICAM 22 was obtained after purification and lyophilization as a white cotton-like solid (30 mg, 41% overall yield). HPLC: 16.91 min (99% analytical purity). HRMS (ESI,+) m/z: calculated for $C_{33}H_{63}N_5O_5$ 609.4823; found 609.4829 (1.09 ppm).

DICAM 23 (H-**Ala**-**Pro**-Glu-NH-$C_{18}H_{37}$ ·HCl). Following general procedure A with 0.114 mmol of resin and Fmoc-(D)-Ala-OH as *N*-terminal residue, the *N*-deprotected DICAM 23 was obtained as a white lyophilized cotton-like solid (33 mg, 48% overall yield) after purification. HPLC: 19.31 min (99% analytical purity). HRMS (ESI,+) m/z: calculated for $C_{33}H_{63}N_5O_5$ 609.4806; found 609.4829 (3.84 ppm).

DICAM 24 (Ac-Ala-**Pro**-Glu-NH-$C_{18}H_{37}$). From 0.114 mmol of resin and using Fmoc-Ala-OH as *N*-terminal residue the general procedure A was followed. Then, the *N*-terminal group of the resin was acetylated, after standard piperidine on-resin Fmoc deprotection, upon treatment with a mixture of Ac_2O:DIEA:DMF (1:1:1, in volume) at room temperature (1 x 1 min) and then (4 x 10 min). The resin was washed with DMF/DCM/DMF/DCM (4 x 0.5 min), precipitated over cold Et_2O and centrifuged three times at 5000 rpm for 10 min. After removing the supernatant, the pellets were redissolved in water/acetonitrile and lyophilized. *N*-acetylated DICAM 24 was obtained as a white lyophilized cotton-like solid (28 mg, 40% overall yield) after purification. HPLC: 21.23 min (98% analytical purity). HRMS (ESI,+) m/z: calculated for $C_{33}H_{60}N_4O_6$ 608.4488; found 608.4513 (3.12 ppm).

S1.1.2 Compounds synthesized by procedure B

DICAM 13 (H-Asn-**Pro**-NH-$C_{18}H_{37}$·HCl). The general **procedure B** was followed using Cbz-Asn(Trt)-OH as *N*-terminal residue and Fmoc-(*D*)-Pro-OH as C-terminal residue. The reaction mixture was heated at 70 °C for 4 h. Cbz deprotection of the final lipopeptide, purification and lyophilization, in the presence of HCl, afforded the *N*-deprotected DICAM 13 as a white cotton-like solid (23 mg, 39% overall yield). HPLC: 20.18 min (98% analytical purity). HRMS (ESI,+) m/z: calculated for $C_{27}H_{52}N_4O_3$ 480.4025; found 480.4039 (3 ppm).

DICAM 14 (H-Ala-**Pro**-Ala-NH-$C_{18}H_{37}$ ·HCl). General **procedure B** was followed with 0.114 mmol of resin using Cbz-Ala(Trt)-OH as *N*-terminal residue and Fmoc-Ala-OH as C-terminal residue. After purification and lyophilization, in the presence of HCl, the hydrochloride of *N*-deprotected DICAM 14 was obtained (32 mg, 51% overall yield) as a white cotton-like solid. HPLC: 19.50 min (99% analytical purity). HRMS (ESI,+) m/z: calculated for $C_{29}H_{56}N_4O_3$ 508.4344; found 508.4352 (1.72 ppm).

DICAM 15 (Cbz-Ala-**Pro**-Trp-NH-$C_{18}H_{37}$). Starting from 0.114 mmol of resin, and following the general procedure B using Cbz-Asn(Trt)-OH as *N*-terminal residue, and Fmoc-Trp-OH as C-terminal residue, *N*-protected **DICAM 15** was isolated, after purification, as a white lyophilized cotton-like solid (55 mg, 63% overall yield). HPLC: 23.18 min (97% analytical purity). HRMS (ESI,+) m/z: calculated for $C_{45}H_{67}N_5O_5$ 757.5136; found 757.5142 (0.86 ppm).

DICAM 16 (H-Asn-**Pro**-Trp-NH-$C_{18}H_{37}$ ·HCl). The general procedure B was followed using Cbz-Asn(Trt)-OH as *N*-terminal residue and Fmoc-Trp-OH as C-terminal residue, hydrochloride of DICAM 16 (36 mg, 48% overall yield) was obtained as a white cotton-like solid after workup. HPLC: 21.04 min (97% analytical purity). HRMS (ESI,+) m/z: calculated for $C_{37}H_{61}N_5O_3$ 623.4766; found 623.4774 (1.42 ppm).

S2 Supplementary Tables

Table S1. General DICAM's structure

DICAMs 1─16, 18─24 (R-Xaa-**Pro**-Yaa-R')

DICAM 17 (H-Asn-**Ala**-Glu-NH-$C_{18}H_{38}$)

Compound	Formulae	R	Xaa	Yaa	R'
DICAM 1	Cbz-Asn-**Pro**-Glu-NH-$C_{18}H_{37}$	Cbz	Asn	Glu	NH-$(CH_2)_{17}CH_3$
DICAM 2	Asn-**Pro**-Glu-NH-$C_{18}H_{37}$ ·HCl	H	Asn	Glu	NH-$(CH_2)_{17}CH_3$
DICAM 3	Cbz-Ala-**Pro**-Glu-NH-$C_{18}H_{37}$	Cbz	Ala	Glu	NH-$(CH_2)_{17}CH_3$
DICAM 4	Ala-**Pro**-Glu-NH-$C_{18}H_{37}$ ·HCl	H	Ala	Glu	NH-$(CH_2)_{17}CH_3$
DICAM 5	Asn-**Pro**-Glu-OMe · HCl	H	Asn	Trp	OMe
DICAM 6	Asn-**Pro**-Tyr(Bzl)-NH-$C_{18}H_{37}$ ·HCl	H	Asn	Tyr(Bzl)	NH-$(CH_2)_{17}CH_3$
DICAM 7	Asn-**Pro**-Phe-NH-$C_{18}H_{37}$ · HCl	H	Asn	Phe	NH-$(CH_2)_{17}CH_3$
DICAM 8	Cbz-Asn-**Pro**-Glu(O^{t}Bu)-NH-$C_{18}H_{37}$	Cbz	Asn	Glu	NH-$(CH_2)_{17}CH_3$
DICAM 9	Cbz-Asn-**Pro**-Glu-NH-$C_{12}H_{25}$	Cbz	Asn	Glu	NH-$(CH_2)_{11}CH_3$
DICAM 10	Cbz-Asn-**Pro**-Glu-NH-$C_{16}H_{33}$	Cbz	Asn	Glu	NH-$(CH_2)_{15}CH_3$
DICAM 11	Ala-**Pro**-Glu-NH-$C_{12}H_{25}$ · HCl	H	Ala	Glu	NH-$(CH_2)_{11}CH_3$
DICAM 12	Ala-**Pro**-Glu-NH-$C_{16}H_{33}$ · HCl	H	Ala	Glu	NH-$(CH_2)_{15}CH_3$
DICAM 13	Asn-**Pro**-NH-$C_{18}H_{37}$ · HCl	H	Asn	-	NH-$(CH_2)_{17}CH_3$
DICAM 14	Ala-**Pro**-Ala-NH-$C_{18}H_{37}$ · HCl	H	Ala	Ala	NH-$(CH_2)_{17}CH_3$
DICAM 15	Cbz-Ala-**Pro**-Trp-NH-$C_{18}H_{37}$	Cbz	Ala	Trp	NH-$(CH_2)_{17}CH_3$
DICAM 16	Ala-**Pro**-Trp-NH-$C_{18}H_{37}$ · HCl	H	Ala	Trp	NH-$(CH_2)_{17}CH_3$
DICAM 17	Asn-**Ala**-Glu-NH-C18H37·HCl	H	Asn	Glu	NH-$(CH_2)_{17}CH_3$
DICAM 18	**Pro**-Glu-NH-$C_{18}H_{37}$ · HCl	H	-	Glu	NH-$(CH_2)_{17}CH_3$
DICAM 19	Cbz-Glu-**Pro**-Glu-NH-$C_{18}H_{37}$	Cbz	Glu	Glu	NH-$(CH_2)_{17}CH_3$
DICAM 20	Glu-**Pro**-Glu-NH-$C_{18}H_{37}$ · HCl	H	Glu	Glu	NH-$(CH_2)_{17}CH_3$
DICAM 21	Cbz-Orn-**Pro**-Glu-NH-$C_{18}H_{37}$	Cbz	Orn	Glu	NH-$(CH_2)_{17}CH_3$
DICAM 22	Orn-**Pro**-Glu-NH-$C_{18}H_{37}$ · HCl	H	Orn	Glu	NH-$(CH_2)_{17}CH_3$
DICAM 23	**Ala**-**Pro**-Glu-NH-$C_{18}H_{37}$ · HCl	H	**Ala**	Glu	NH-$(CH_2)_{17}CH_3$
DICAM 24	Ac-Ala-**Pro**-Glu-NH-$C_{18}H_{37}$	Ac	Ala	Glu	NH-$(CH_2)_{17}CH_3$

Bold, underlined residues represent D-amino acids.

Table S2. Bacterial strains			
**Organism**	**Strain**	**Notes**	**References/Origin**
E. coli	DH10B	Laboratory strain	Durfee et al., 2001
P. aeruginosa	PAO1	Standard laboratory strain	CIB[a]
S. pneumoniae	R6	Laboratory D39 derivative. Non-encapsulated	Hosking et al., 2001
	D39	Parental strain of R6. Serotype 2	Lanie et al., 2007
	P046	R6 but *lytA*⁻, *lytC*⁻	Moscoso et al., 2006
	48	Multidrug resistant strain. Serotype 23F	Ramos-Sevillano et al., 2012
S. aureus [T]	ATCC 12600	Type strain	ATCC[b]
S. pyogenes[T]	ATCC 12344	Type strain	ATCC[b]

S3 Supplementary Figures

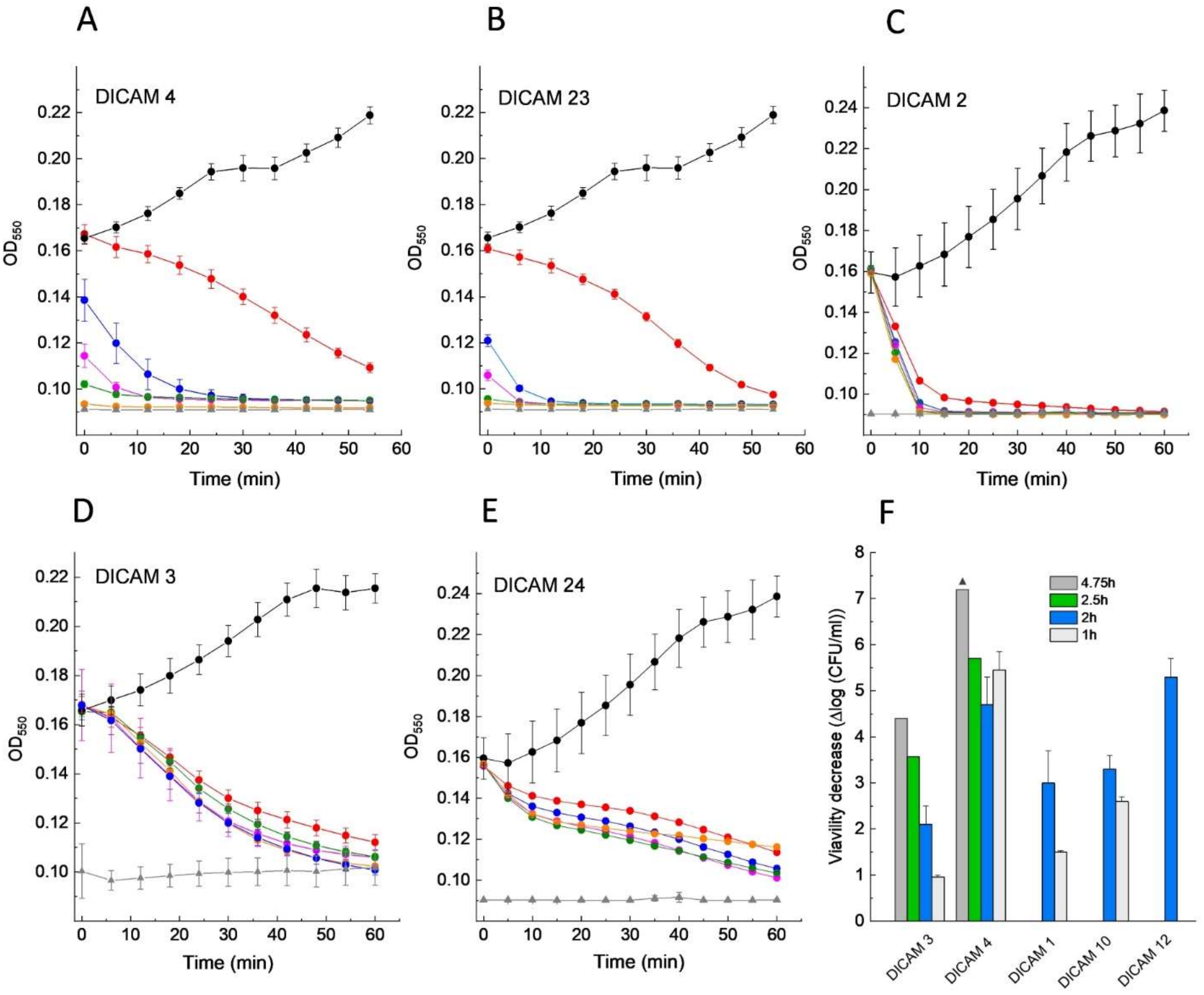

Supplementary Figure S1. **(A–E) OD_{550} kinetics of *S. pneumoniae* D39 lysis by a selection of DICAMs with high (A–C) or moderate (D, E) activity against this pathogen**. Representative time-course profiles (average of three replicas each) at increasing compound doses (10, 17, 25, 50 and 100 µM; red, blue, magenta, green and orange symbols, respectively) are shown. Black and grey symbols depicted the growth of controls and C+Y background, respectively. **F**) Time dependence of the killing rate of selected DICAMs (50 µM). Highly active compounds like DICAM 4 nearly sterilize the culture in less than 1 h, while moderately active ones such as DICAMs 1, 3, or 10 can reduce initial inoculum viability by 99.9% in around 2 h. The black triangle (▲) indicates sterilization of the bacterial suspension within the detection limit (no colonies counted in 100 µL of undiluted samples). All measurements were performed at 37 °C in C+Y, and error bars represent the standard deviation from replicates (A–E) or independent experiments (F).

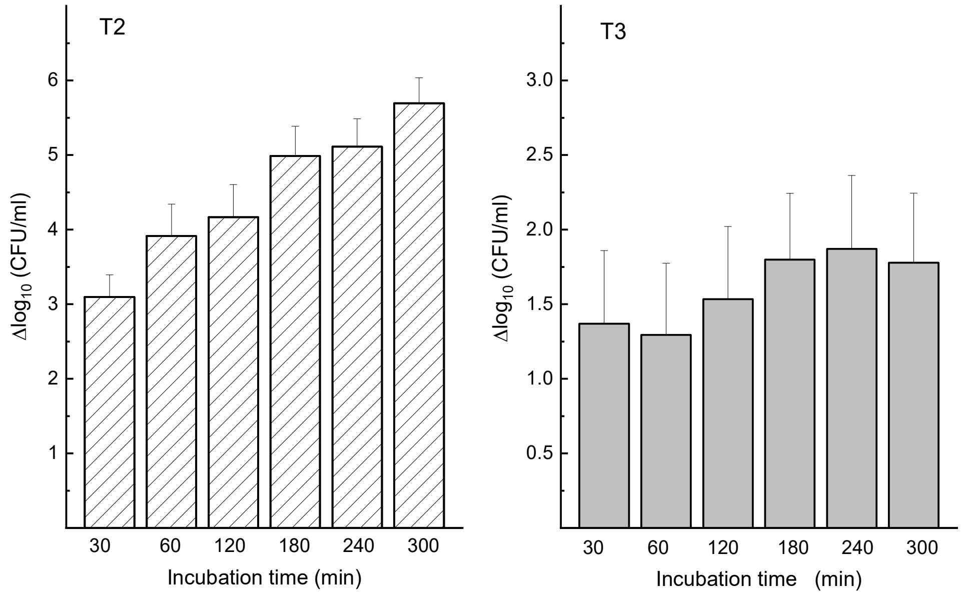

Supplementary Figure S2. **Time-dependence of *S. pneumoniae* D39 viability (C+Y; 37 °C) after culture challenge with 50 µM DICAM 4 at T2 (OD_{550} = 0.37) or T3 (OD_{550} = 0.6)**. Each value represents the average of three replicas and error bars the standard error of the mean.

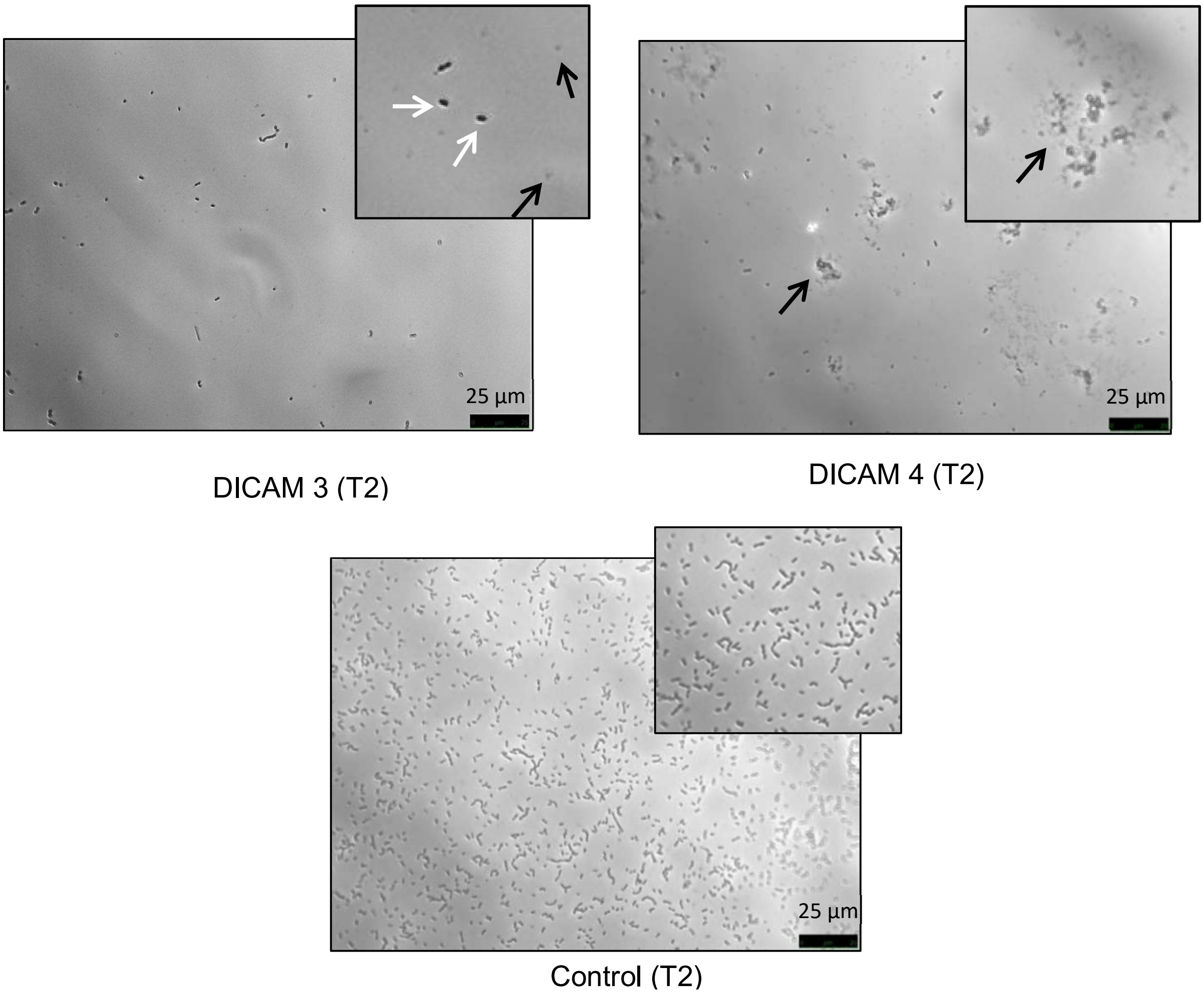

Supplementary Figure S3. Contrast phase microscopy of *S. pneumoniae* R6 strain treated or not with DICAM 3 or DICAM 4 (50 µM) at T2 (OD550 ≈ 0.3). Cultures were incubated for 60 min after DICAM addition (37 °C, C+Y) and then visualized (40×), showing a drastic reduction in the population of intact cells after the treatment. Black arrows indicate apparently empty cocci or cellular debris, and white arrows unlysed bacteria.

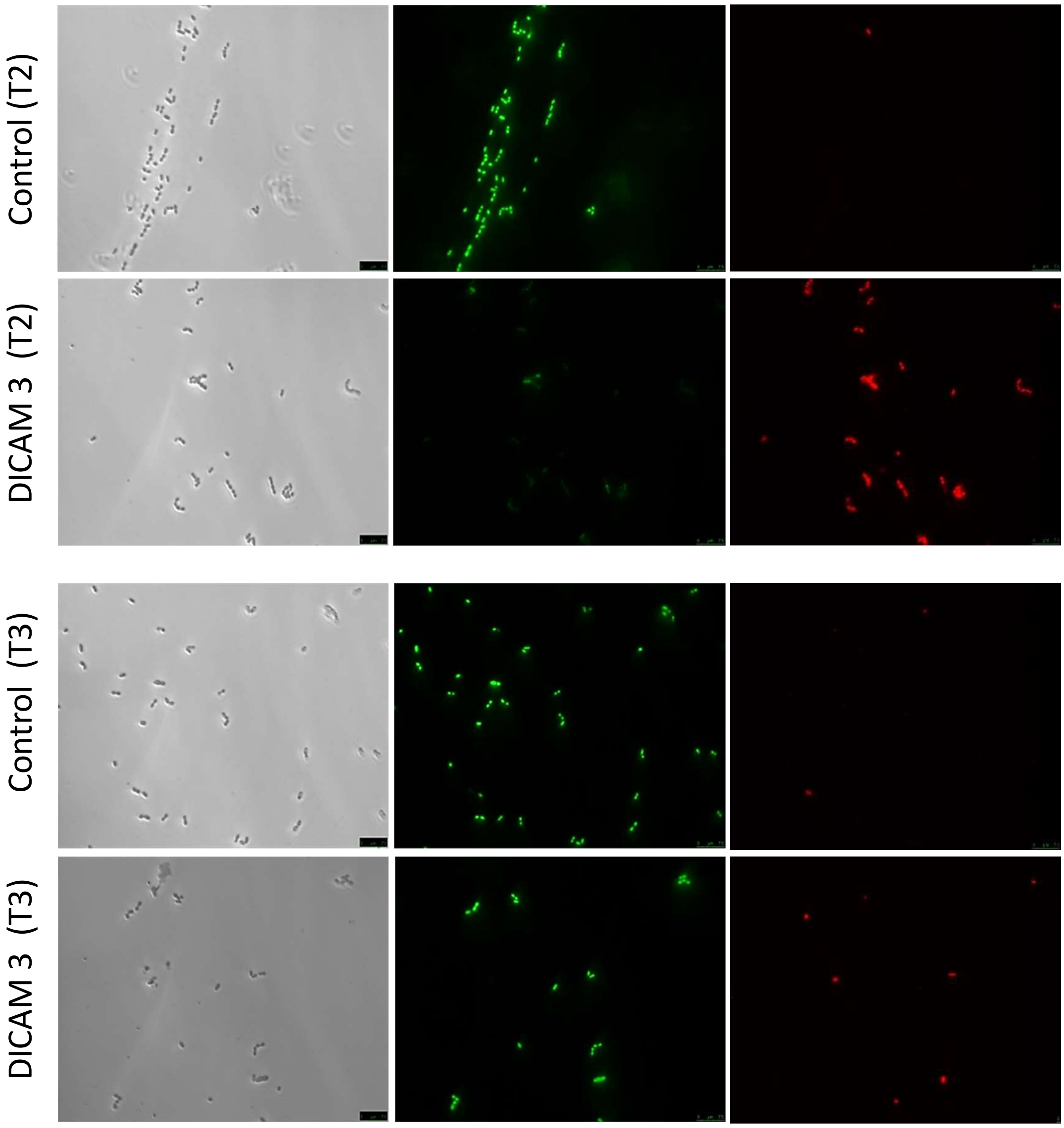

Supplementary Figure S4. **Fluorescence microscopy images *of S. pneumoniae* R6 treated (30 min, 37 °C) and untreated with 50 µM DICAM 3 added at either T2 (OD_{550} ≈ 0.3) or T3 (OD_{550} ≈ 0.6)**. Then, cells were stained with the live/dead Baclight bacterial viability kit and visualized at 100×. Bacteria with intact or permeabilized membrane fluoresce green and red, respectively. Left panels are the phase contrast micrographs. Black bars represent 75 µm.

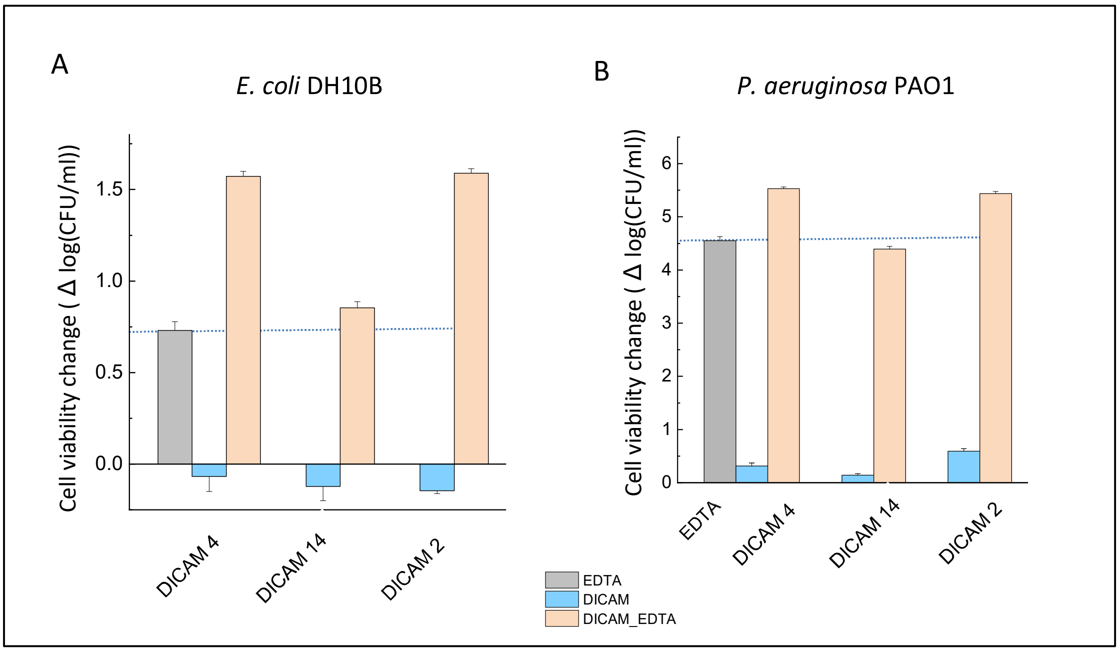

Supplementary Figure S5. **Viability decrease of A) *E. coli* DH10B, and B) *P. aeruginosa* PAO1 upon treatment (3 h, 37 °C) with 100 µM of the indicated DICAM in the absence (blue bars) and in the presence (orange bars) of 0.5 mM EDTA** with relation to controls carried out in buffer (20 mM Tris-HCl, 0.25% DMSO, pH 7.5). Grey bars show the reduction of cell viability induced by the outer membrane permeating agent (EDTA) in these conditions. Average values and data errors from a representative experiment with two-to-three replicas for each condition are shown.

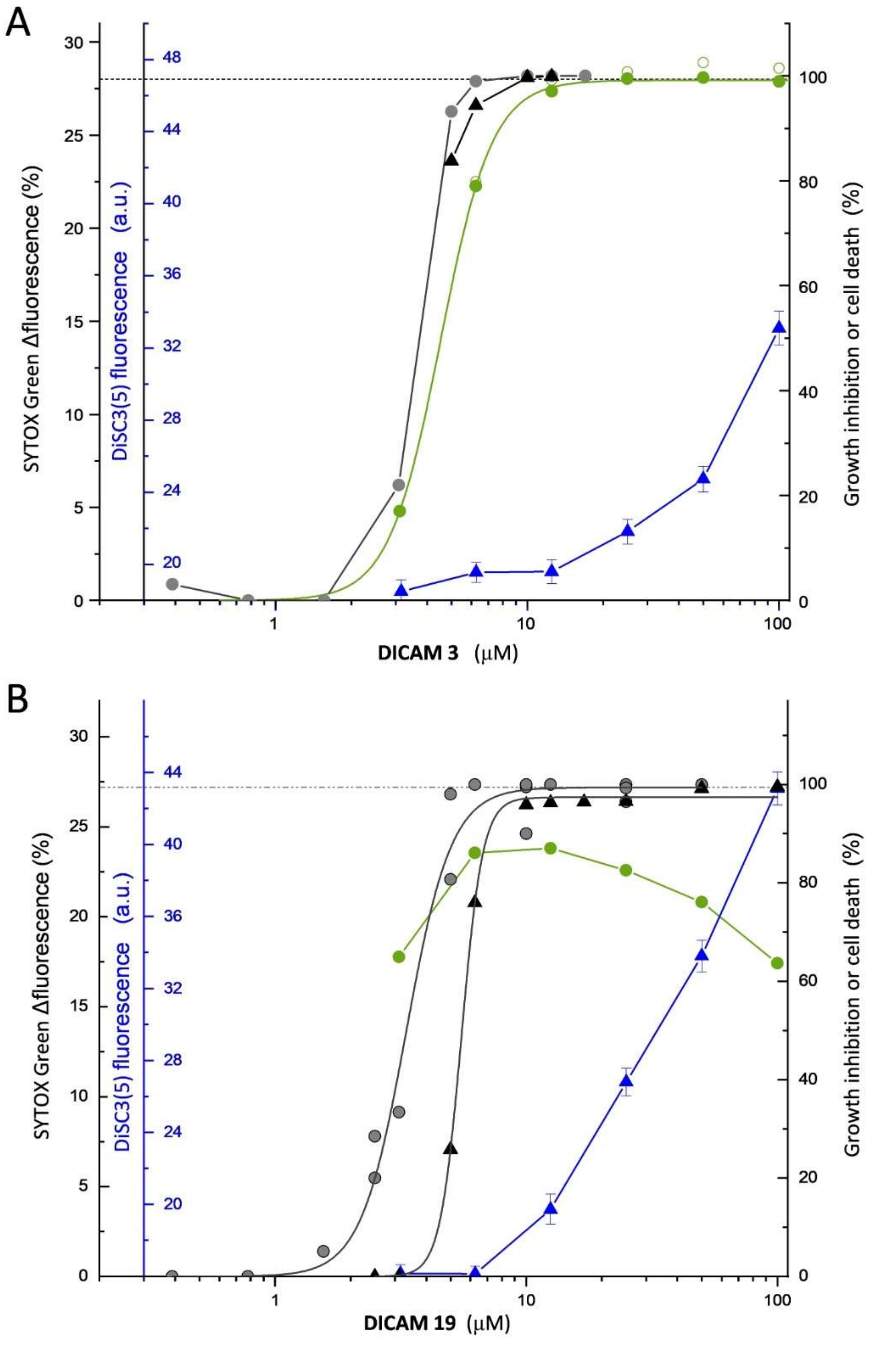

Supplementary Figure S6. **Dependence of *S. pyogenes*[T] growth, viability and membrane integrity on added doses of DICAM 3 (A) or DICAM 19 (B)**. Membrane perturbation was evaluated by SYTOX Green and DiSC3(5) assays as detailed in the text (see also Figure 9). The fluorescence signal attained at the equilibrium was plotted as the percentage of membrane permeation (SYTOX Green; green circles), or in arbitrary units for depolarization (DiSC3(5); blue triangles and left Y-blue axis). Bacterial viability is shown as the percentage of CFU reduction after 120 min treatment when compared to untreated controls (black triangles), and growth inhibition as the percentage increase in the OD_{550} values of treated *vs* untreated cultures in the same time interval (grey circles).

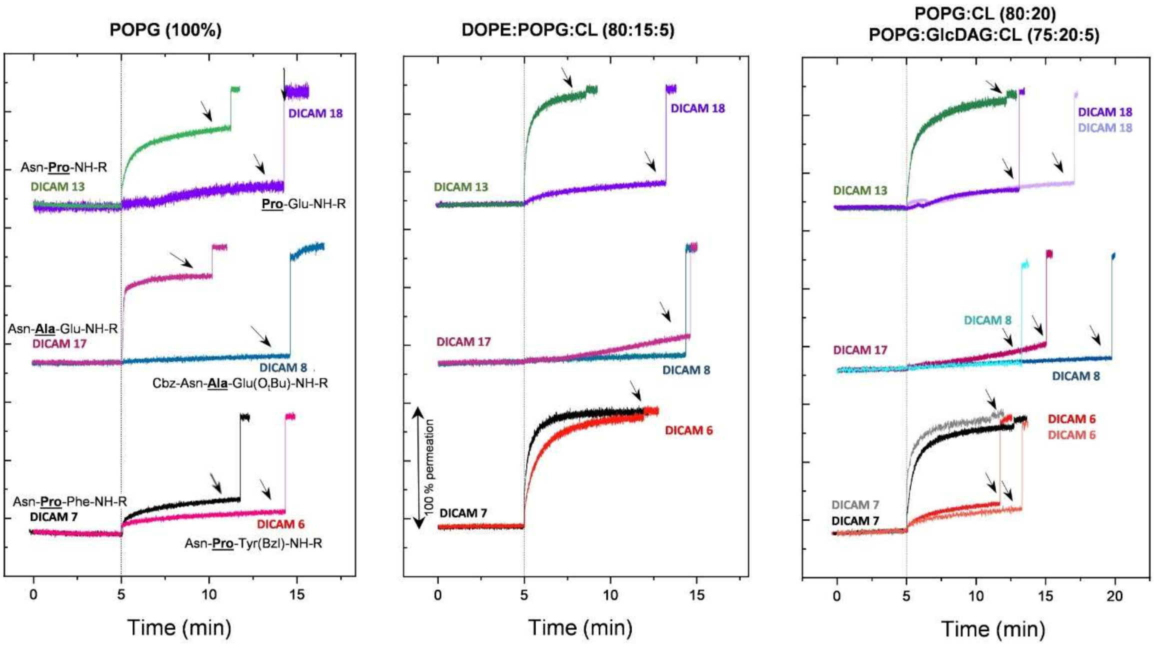

Supplementary Figure S7. Permeation of LUVs by non-antibacterial DICAMs. Kinetics of calcein release from LUVs of different compositions by DICAMs without antibacterial activity. After recording the basal fluorescence for 5 min, DICAMs were added (10 µM final concentration). The change in fluorescence (λ_{ex} = 480 nm, λ_{em} = 550 nm) was monitored and plotted as the percentage of membrane permeabilization relative to the total increment obtained after treating LUVs with 0.025% v/v Triton-X100 at the end of the experiment. Addition of Triton-X100 is indicated by the black arrows**.** DICAM-untreated LUVs were taken as positive controls. Assays were carried out at 25 °C in vesicle buffer (10 mM Tris, 140 mM NaCl, 0.5 mM EDTA, pH 7.4). The figure labels indicated the color code and curves were shifted along the Y-axis for clarity. (A) pure POPG, (B) DOPE:POPG:CL 80:15:5, and (C) POPG:CL 80:20 (violet, navy, black, and orange traces) or POPG:GlcDAP:CL 75:20:5 (green, light violet, purple, cyan, grey and red traces).

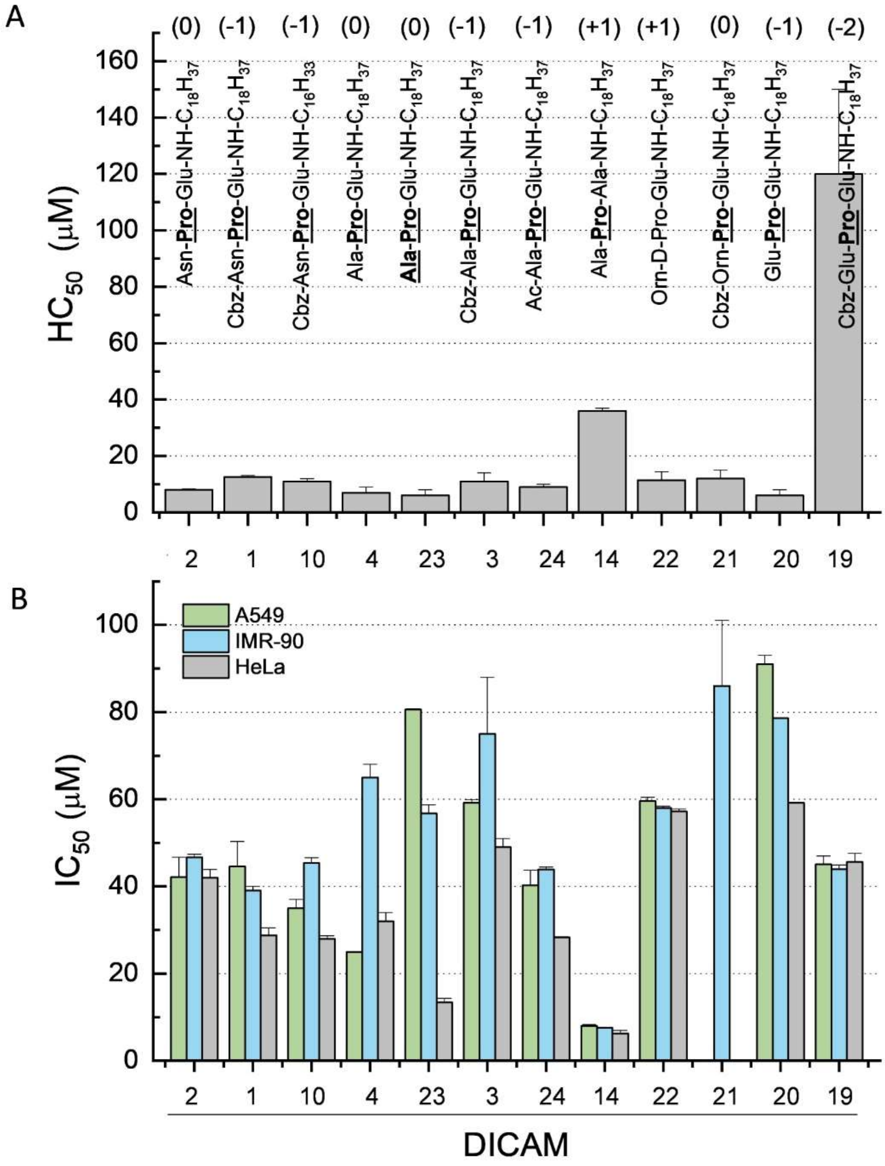

Supplementary Figure S8. **A) Hemolytic activity (1 h treatment at 37 °C), and B) cytotoxicity against three different cell lines (48 h treatment at 37 °C) of selected DICAMs**. HC_{50} and IC_{50} values are the mean ± standard error of at least three independent experiments with error bars indicating the standard deviation of the mean. Data in parenthesis in A) represents the net charge. Combination of Glu as the first amino acid of the tripeptide with Cbz-capping drastically reduced hemolytic activity. Toxicities for a given cell line can vary by a factor of two due to differences in the *N*-terminal amino acid, capping, or fatty amine length, with no obvious trend with hydrophobicity.

S4 HPLC chromatograms

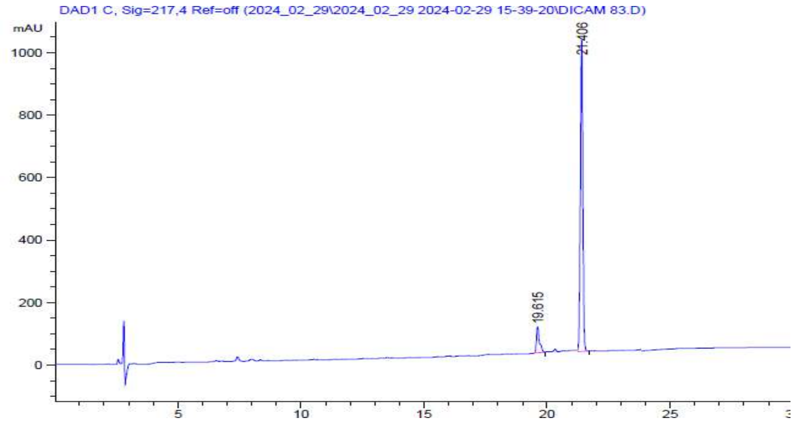

HPLC of DICAM 1

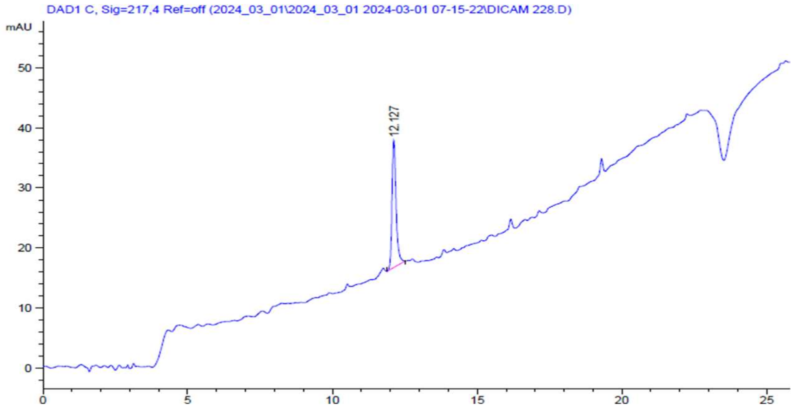

HPLC of DICAM 2

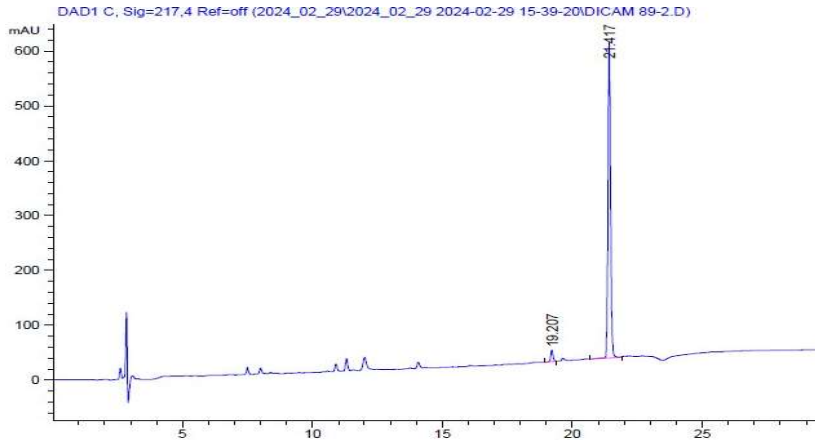

HPLC of DICAM 3

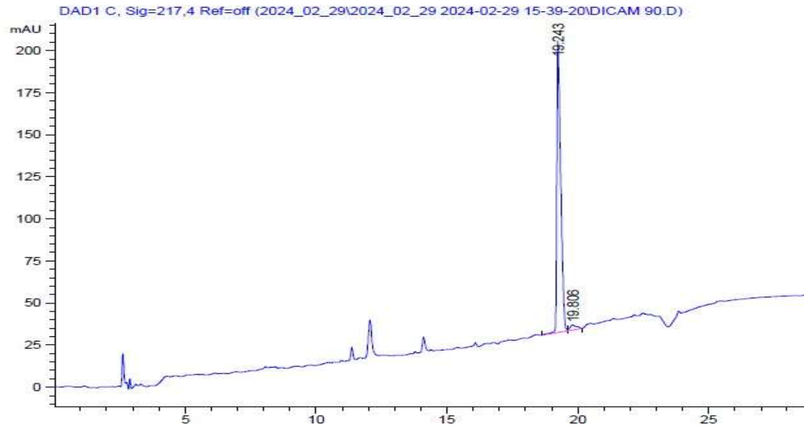

HPLC of DICAM 4

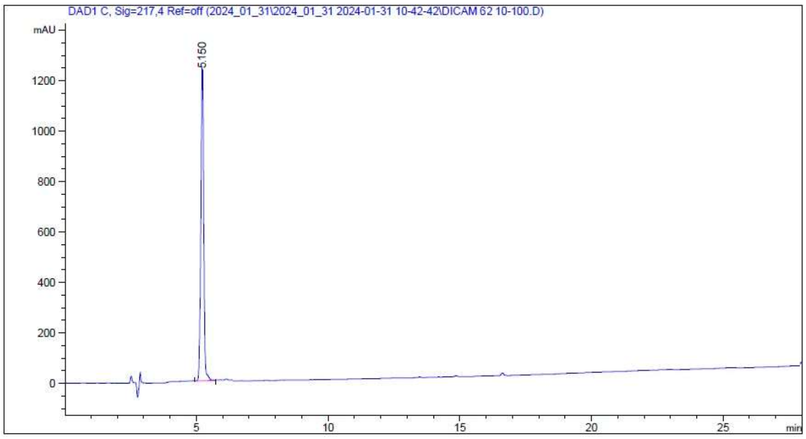

HPLC of DICAM 5

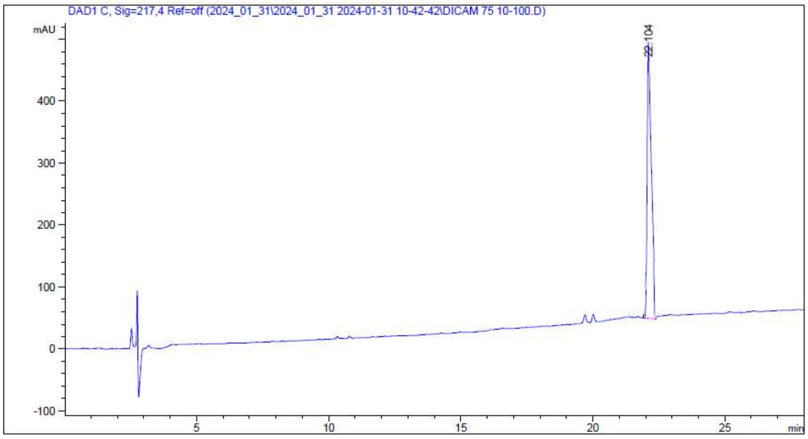

HPLC of compound DICAM 6

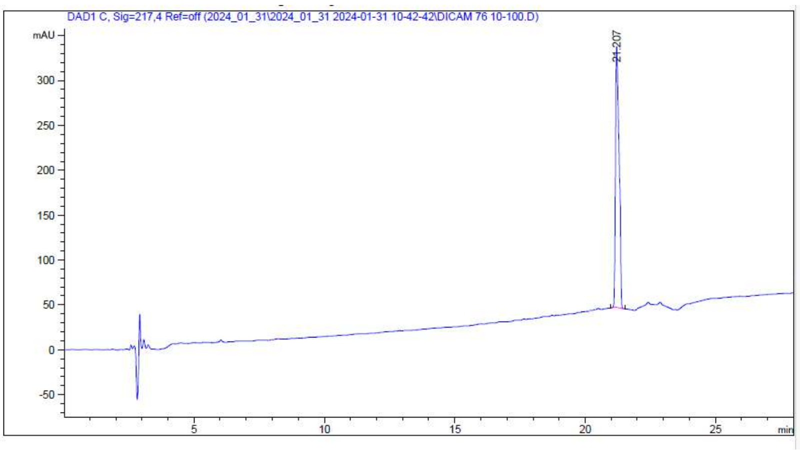

HPLC of DICAM 7

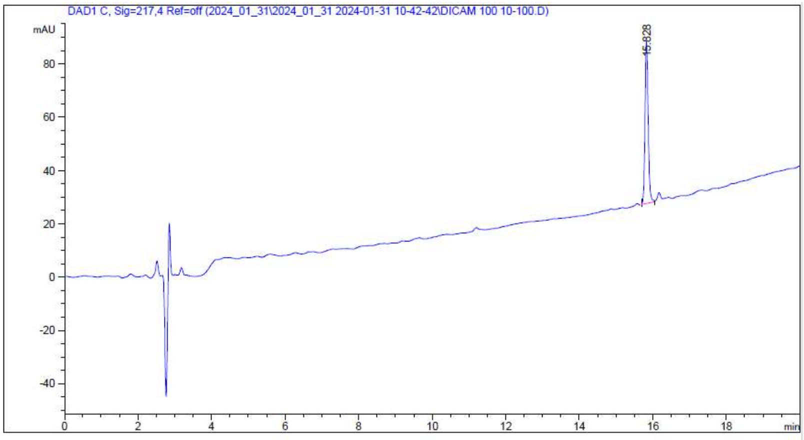

HPLC of DICAM 9

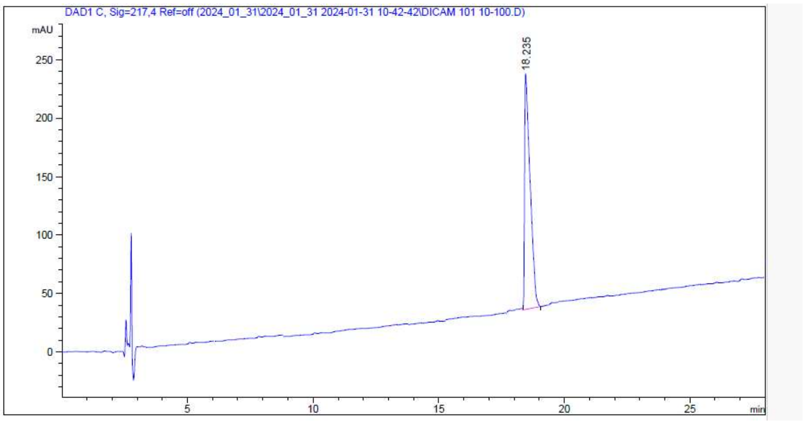

HPLC of DICAM 10

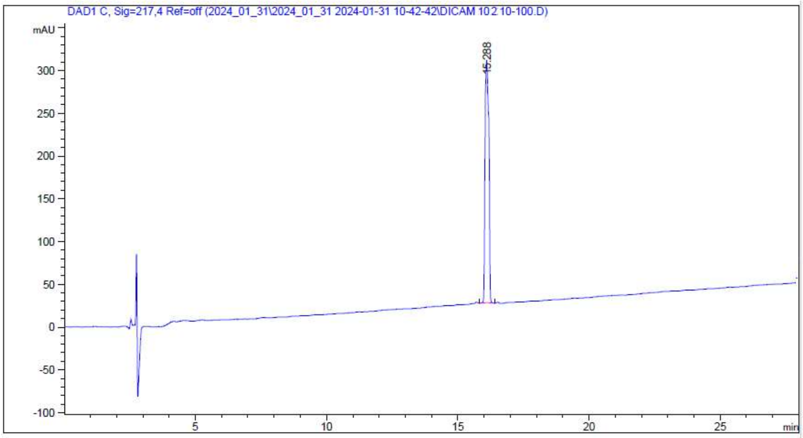

HPLC of DICAM 11

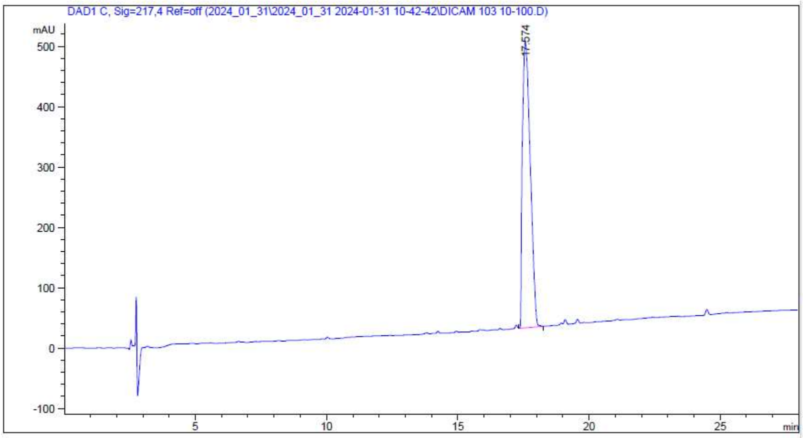

HPLC of DICAM 12

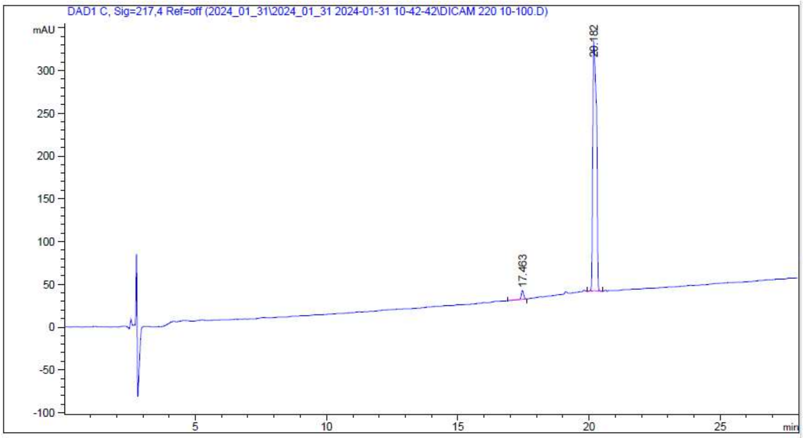

HPLC of DICAM 13

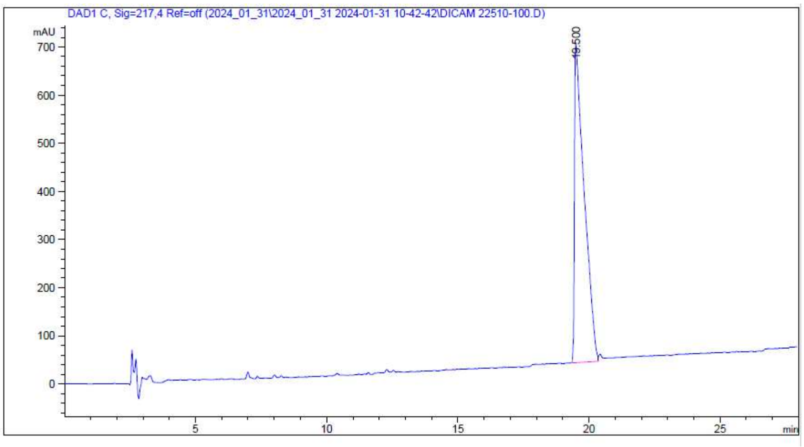

HPLC of DICAM 14

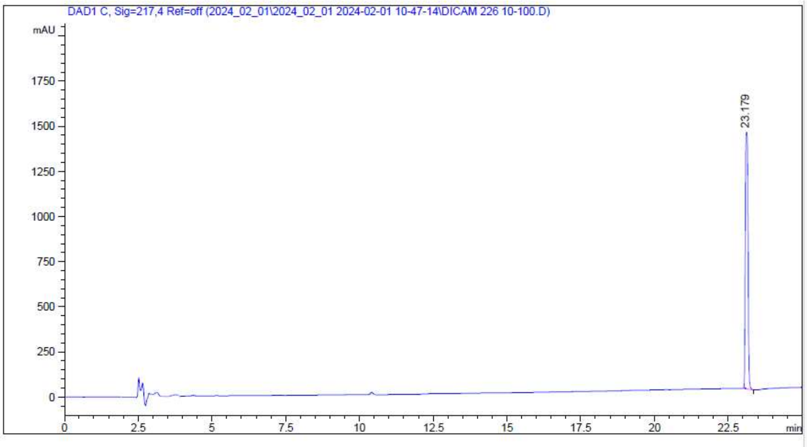

HPLC of DICAM 15

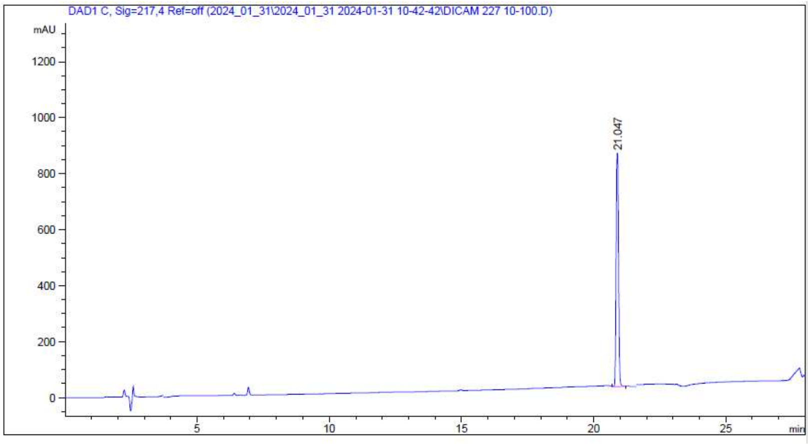

HPLC of DICAM 16

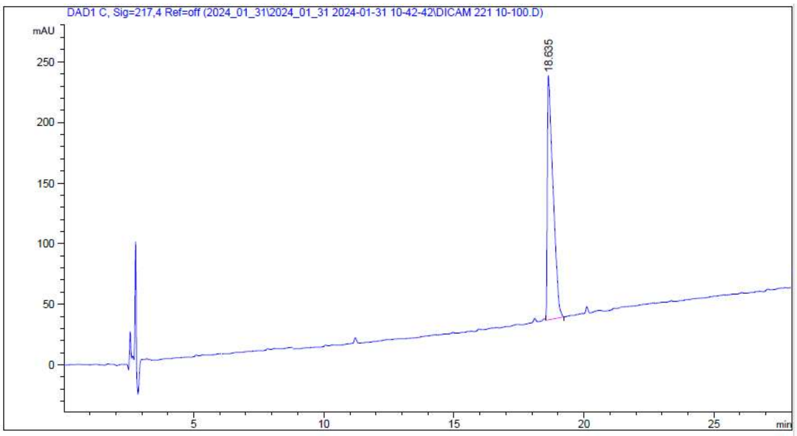

HPLC of DICAM 17

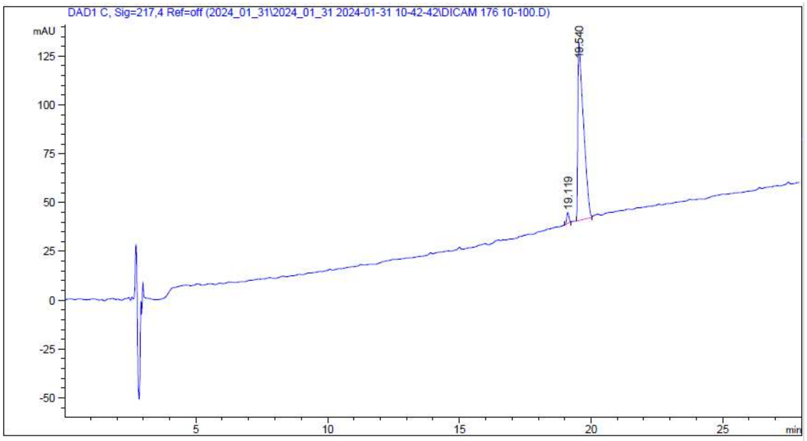

HPLC of DICAM 18

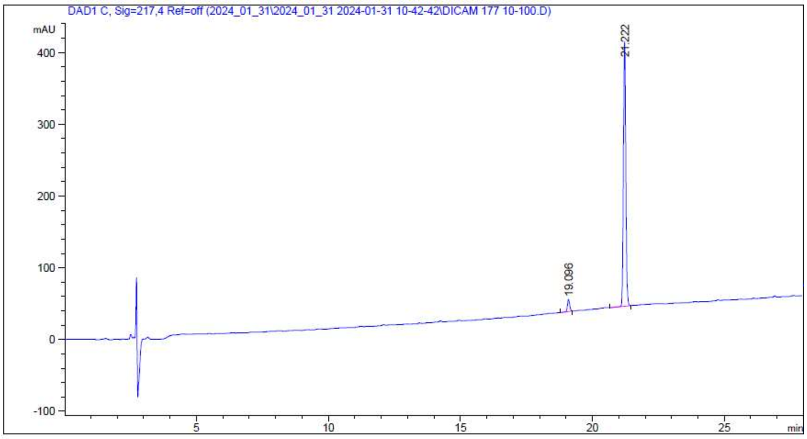

HPLC of DICAM 19

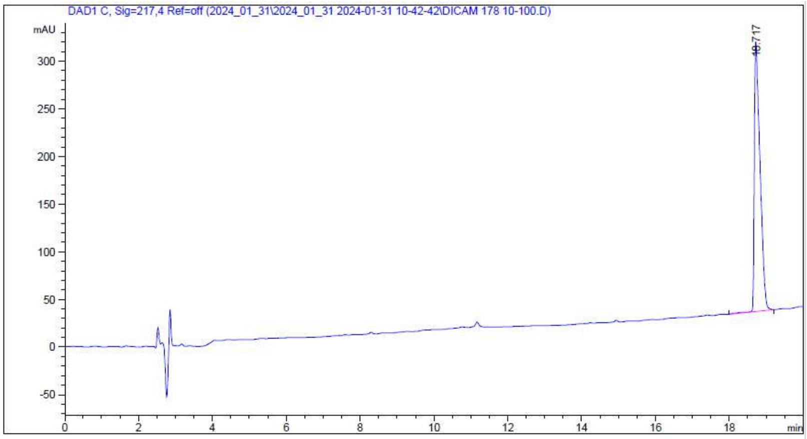

HPLC of DICAM 20

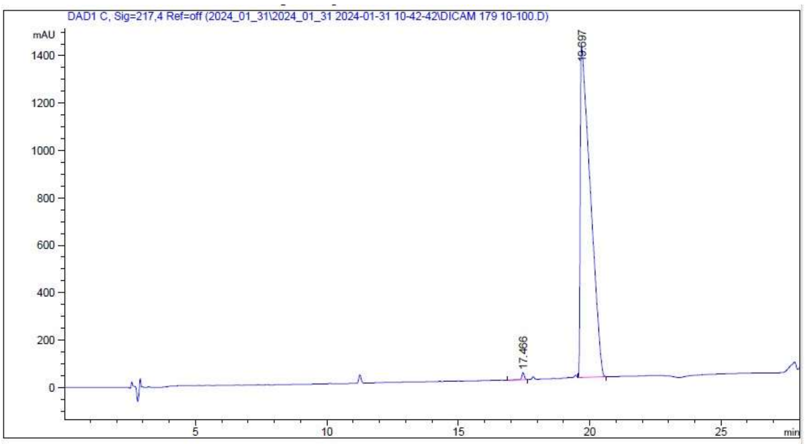

HPLC of DICAM 21

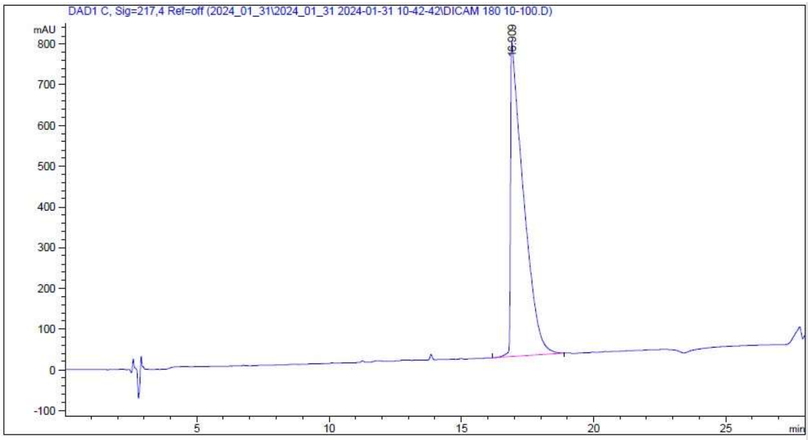

HPLC of DICAM 22

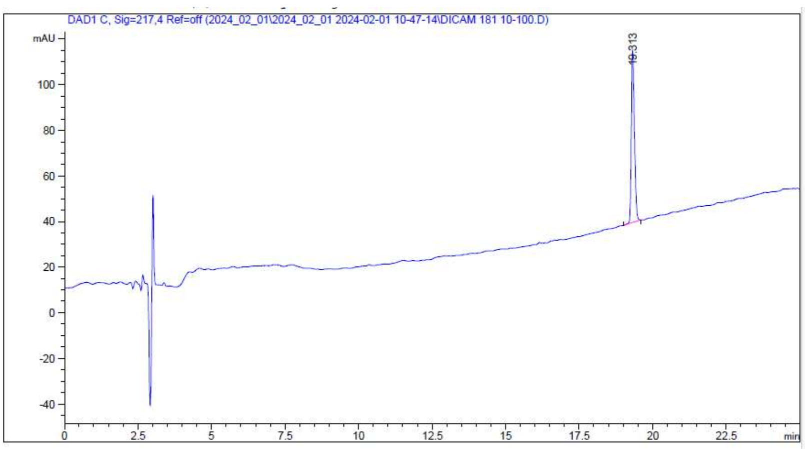

HPLC of DICAM 23

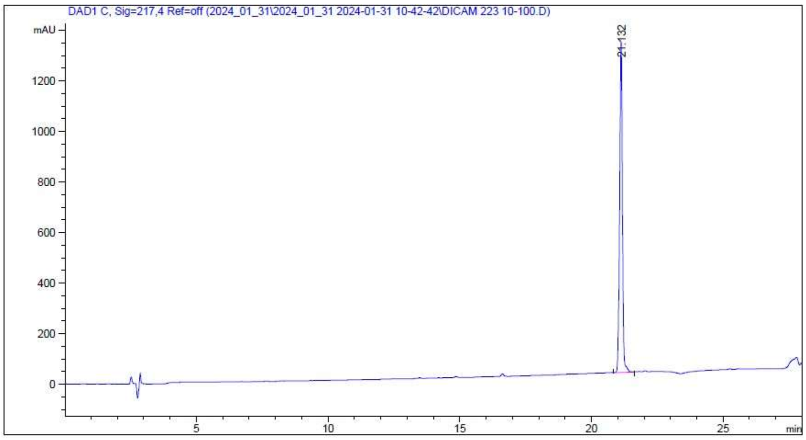

HPLC of DICAM 24

S5 HRMS spectra

Compound Table

Compound Label	RT	Mass	Abund	Formula	Tgt Mass	Diff (ppm)	Hits (DB)
Cpd 1: C40 H65 N5 O8; 1.981	1.981	743.4833	653206	C40 H65 N5 O8	743.4853	-2.65	1

Compound Label	*m/z*	RT	Algorithm	Mass
Cpd 1: C40 H65 N5 O8; 1.981	744.4912	1.981	Find by Formula	743.4833

MS Zoomed Spectrum

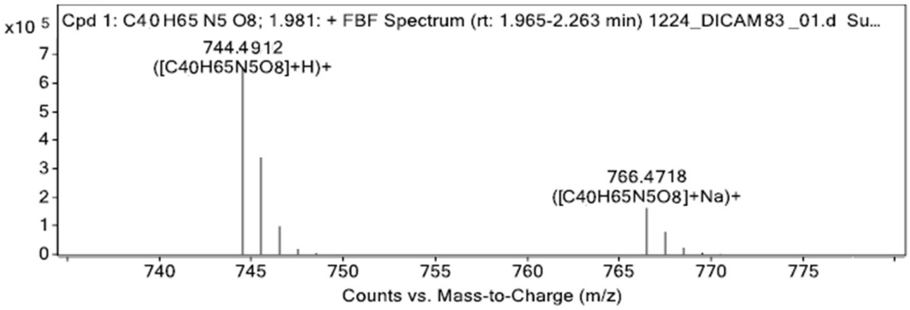

HMRS spectra of DICAM 1

Compound Table

Compound Label	RT	Mass	Abund	Formula	Tgt Mass	Diff (ppm)	Hits (DB)
Cpd 1: C39 H64 N4 O7; 1.623	1.623	700.4765	84738	C39 H64 N4 O7	700.4775	-1.4	1

Compound Label	*m/z*	RT	Algorithm	Mass
Cpd 1: C39 H64 N4 O7; 1.623	701.4835	1.623	Find by Formula	700.4765

MS Zoomed Spectrum

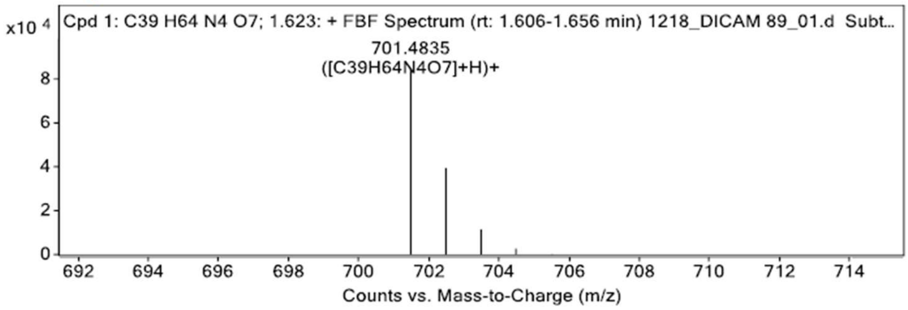

HMRS spectra of DICAM 3

Compound Table

Compound Label	RT	Mass	Abund	Formula	Tgt Mass	Diff (ppm)	Hits (DB)
Cpd 1: C31 H58 N4 O5; 2.822	2.822	566.4393	152442	C31 H58 N4 O5	566.4407	-2.59	1

Compound Label	*m/z*	RT	Algorithm	Mass
Cpd 1: C31 H58 N4 O5; 2.822	589.4288	2.822	Find by Formula	566.4393

MS Zoomed Spectrum

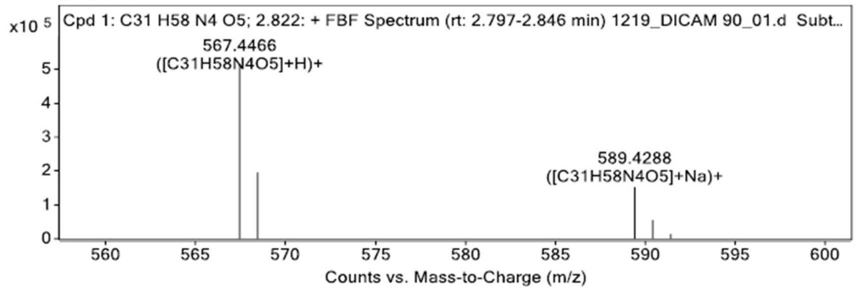

HMRS spectra of DICAM 4

Compound Table

Compound Label	RT	Mass	Abund	Formula	Tgt Mass	Diff (ppm)	Hits (DB)
Cpd 1: C15 H24 N4 O7; 0.296	0.296	372.1645	557748	C15 H24 N4 O7	372.1660	-2.74	1

Compound Label	*m/z*	RT	Algorithm	Mass
Cpd 1: C15 H24 N4 O7; 0.296	395.1549	0.296	Find by Formula	372.1645

MS Zoomed Spectrum

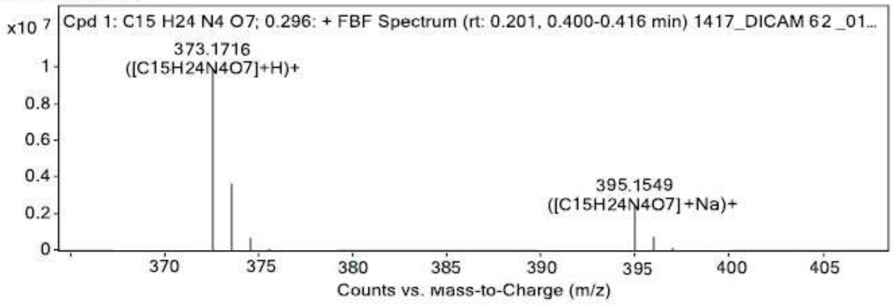

HMRS spectra of DICAM 5

Compound Table

Compound Label	RT	Mass	Abund	Formula	Tgt Mass	Diff (ppm)	Hits (DB)
Cpd 1: C43 H67 N5 O5; 1.981	1.981	733.5142	653206	C43 H67 N5 O5	733.5122	-2.68	1

Compound Label	*m/z*	RT	Algorithm	Mass
Cpd 1: C43 H67 N5 O5; 1.981	734.5201	1.981	Find by Formula	733.5142

MS Zoomed Spectrum

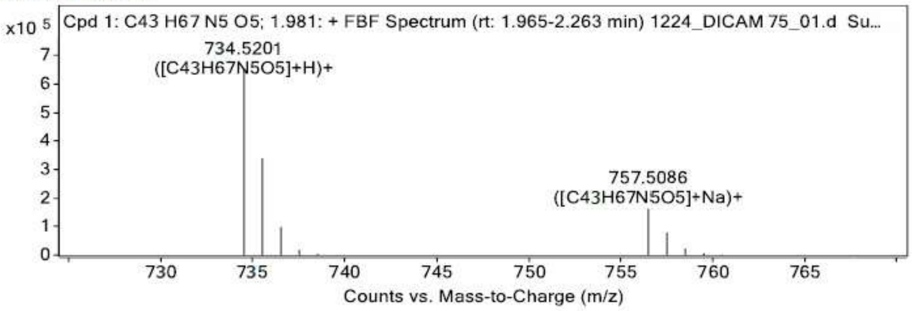

HMRS spectra of DICAM 6

Compound Table

Compound Label	RT	Mass	Abund	Formula	Tgt Mass	Diff (ppm)	Hits (DB)
Cpd 1: C36 H61 N5 O4; 1.443	1.443	627.4712	638785	C36 H61 N5 O4	627.4724	-1.86	1

Compound Label	*m/z*	RT	Algorithm	Mass
Cpd 1: C36 H61 N5 O4; 1.443	650.4613	1.443	Find by Formula	627.4712

MS Zoomed Spectrum

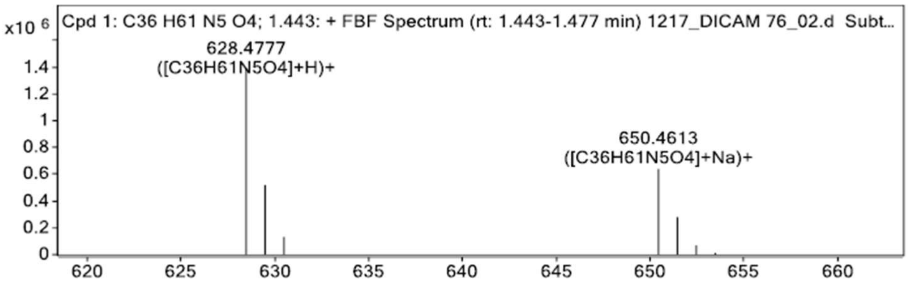

Counts vs. Mass-to-Charge (m/z)

HMRS spectra of DICAM 7

Compound Table

Compound Label	RT	Mass	Abund	Formula	Tgt Mass	Diff (ppm)	Hits (DB)
Cpd 1: C44 H73 N5 O8; 1.825	1.825	799.5459	283770	C44 H73 N5 O8	799.5464	-0.64	1

Compound Label	*m/z*	RT	Algorithm	Mass
Cpd 1: C44 H73 N5 O8; 1.825	822.5354	1.825	Find by Formula	799.5459

MS Zoomed Spectrum

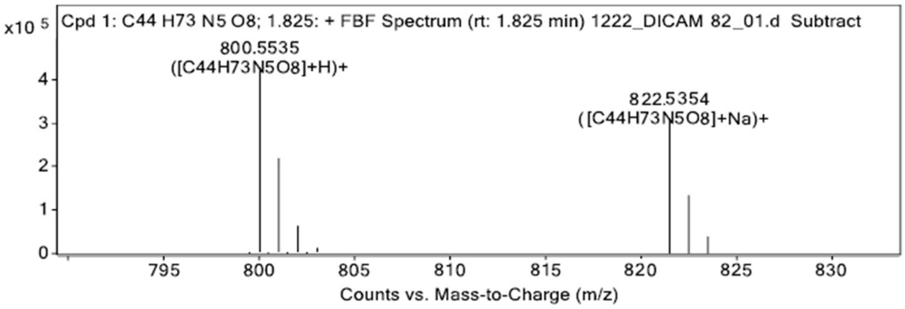

HMRS spectra of DICAM 8

Compound Table

Compound Label	RT	Mass	Abund	Formula	Tgt Mass	Diff (ppm)	Hits (DB)
Cpd 1: C34 H53 N5 O8; 0.280	0.28	659.3887	561521	C34 H53 N5 O8	659.3894	-1.02	1

Compound Label	*m/z*	RT	Algorithm	Mass
Cpd 1: C34 H53 N5 O8; 0.280	682.3788	0.28	Find by Formula	659.3887

MS Zoomed Spectrum

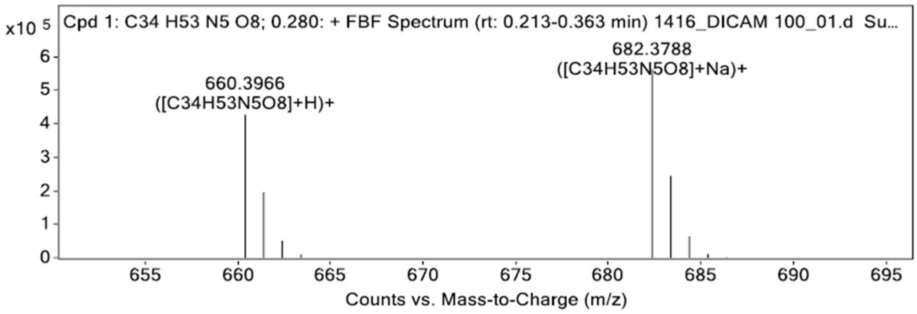

HMRS spectra of DICAM 9

Compound Table

Compound Label	RT	Mass	Abund	Formula	Tgt Mass	Diff (ppm)	Hits (DB)
Cpd 1: C38 H61 N5 O8; 1.623	1.623	715.4520	84738	C38 H61 N5 O8	715.4526	-1.09	1

Compound Label	*m/z*	RT	Algorithm	Mass
Cpd 1: C38 H61 N5 O8; 1.623	716.4595	1.623	Find by Formula	715.4520

MS Zoomed Spectrum

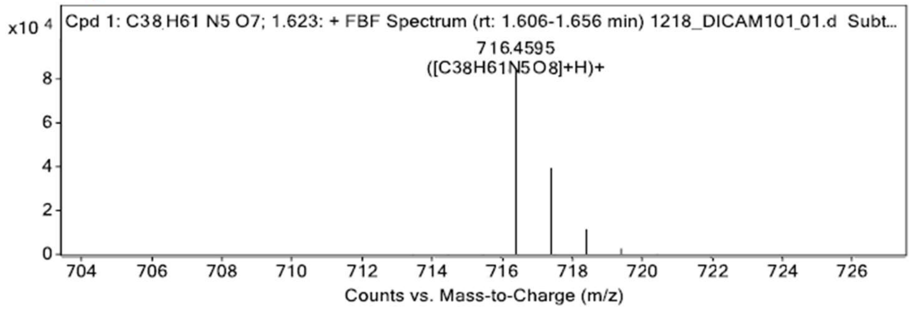

HMRS spectra of DICAM 10

Compound Table

Compound Label	RT	Mass	Abund	Formula	Tgt Mass	Diff (ppm)	Hits (DB)
Cpd 1: C25 H46 N4 O5; 1.467	1.467	482.345	187984	C25 H46 N4 O5	482.3468	-3.79	1

Compound Label	*m/z*	RT	Algorithm	Mass
Cpd 1: C25 H46 N4 O5; 1.467	483.3521	1.467	Find by Formula	482.345

MS Zoomed Spectrum

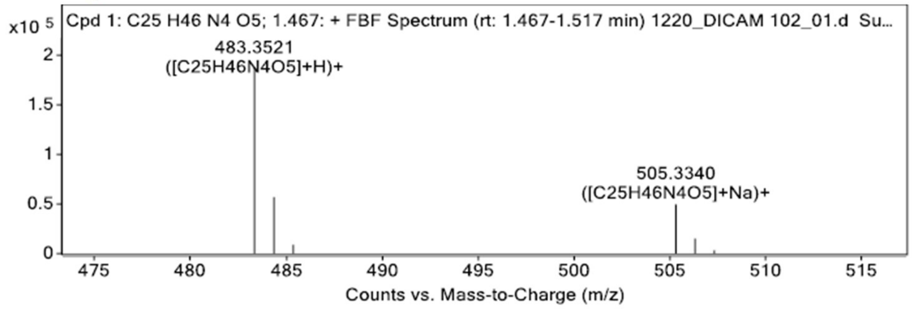

HMRS spectra of DICAM 11

Compound Table

Compound Label	RT	Mass	Abund	Formula	Tgt Mass	Diff (ppm)	Hits (DB)
Cpd 1: C29 H54 N4 O5; 0.296	0.296	538.4079	557748	C29 H54 N4 O5	538.4094	-2.74	1

Compound Label	*m/z*	RT	Algorithm	Mass
Cpd 1: C29 H54 N4 O5; 0.296	561.3983	0.296	Find by Formula	538.4079

MS Zoomed Spectrum

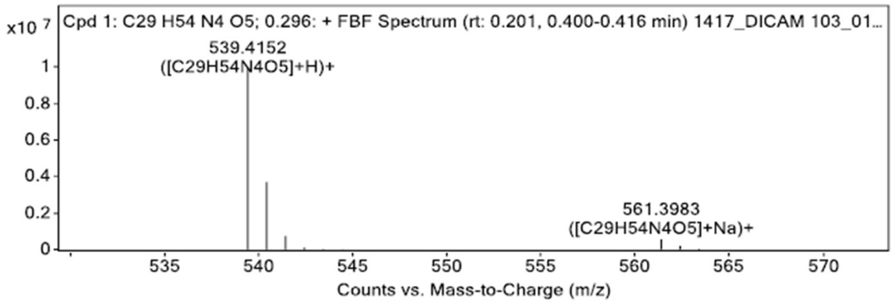

HMRS spectra of DICAM 12

Compound Table

Compound Label	RT	Mass	Abund	Formula	Tgt Mass	Diff (ppm)	Hits (DB)
Cpd 1: C27 H52 N4 O3; 2.062	2.062	480.4025	531867	C27 H52 N4 O3	480.4039	-3	1

Compound Label	*m/z*	RT	Algorithm	Mass
Cpd 1: C27 H52 N4 O3; 2.062	481.4103	2.062	Find by Formula	480.4025

MS Zoomed Spectrum

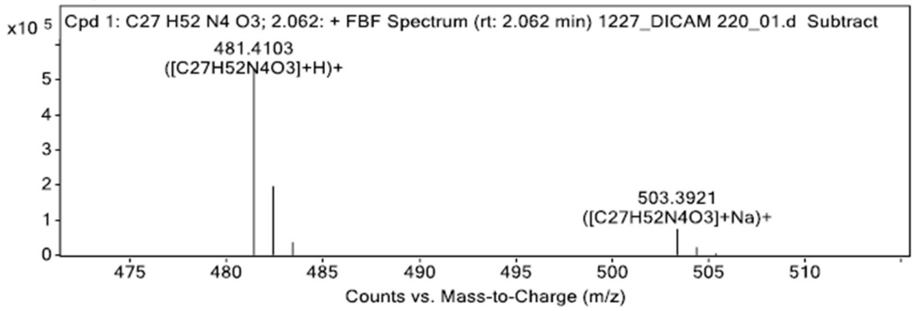

HMRS spectra of DICAM 13

Compound Table

Compound Label	RT	Mass	Abund	Formula	Tgt Mass	Diff (ppm)	Hits (DB)
Cpd 1: C29 H56 N4 O3; 2.646	2.646	508.4344	203970	C29 H56 N4 O3	508.4352	-1.72	1

Compound Label	*m/z*	RT	Algorithm	Mass
Cpd 1: C29 H56 N4 O3; 2.646	509.4418	2.646	Find by Formula	508.4344

MS Zoomed Spectrum

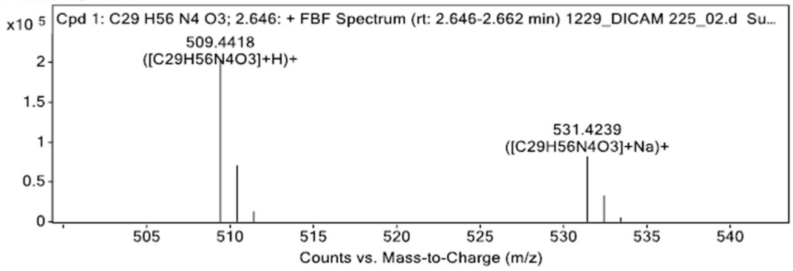

HMRS spectra of DICAM 14

Compound Table

Compound Label	RT	Mass	Abund	Formula	Tgt Mass	Diff (ppm)	Hits (DB)
Cpd 1: C45 H67 N5 O5; 0.392	0.392	757.5136	43372	C45 H67 N5 O5	757.5142	-0.86	1

Compound Label	*m/z*	RT	Algorithm	Mass
Cpd 1: C45 H67 N5 O5; 0.392	780.503	0.392	Find by Formula	757.5136

MS Zoomed Spectrum

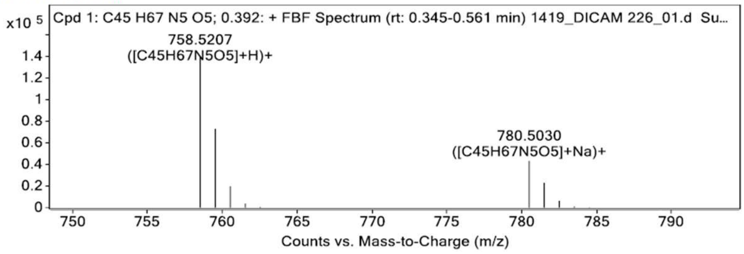

HMRS spectra of DICAM 15

Compound Table

Compound Label	RT	Mass	Abund	Formula	Tgt Mass	Diff (ppm)	Hits (DB)
Cpd 1: C37 H61 N5 O3; 0.276	0.276	623.4766	466098	C37 H61 N5 O3	623.4774	-1.42	1

Compound Label	*m/z*	RT	Algorithm	Mass
Cpd 1: C37 H61 N5 O3; 0.276	646.4668	0.276	Find by Formula	623.4766

MS Zoomed Spectrum

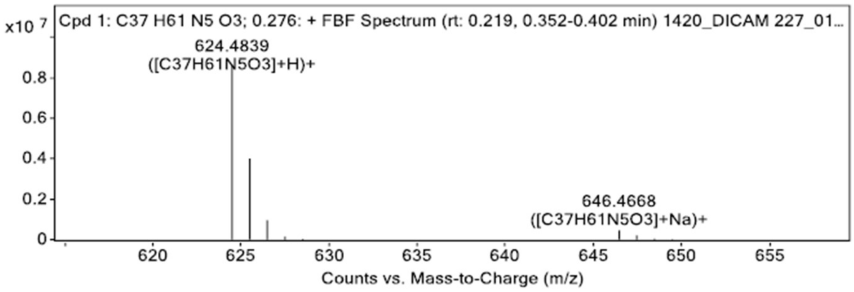

HMRS spectra of DICAM 16

Compound Table

Compound Label	RT	Mass	Abund	Formula	Tgt Mass	Diff (ppm)	Hits (DB)
Cpd 1: C30 H57 N5 O6; 2.508	2.508	583.43	883456	C30 H57 N5 O6	583.4309	-1.54	1

Compound Label	*m/z*	RT	Algorithm	Mass
Cpd 1: C30 H57 N5 O6; 2.508	606.4199	2.508	Find by Formula	583.43

MS Zoomed Spectrum

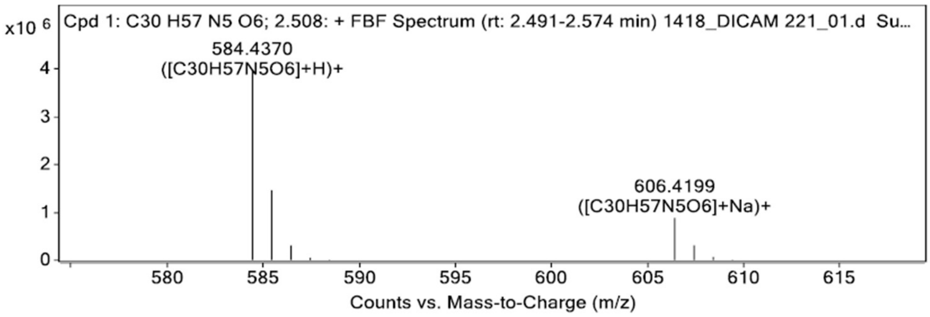

HMRS spectra of DICAM 17

Compound Table

Compound Label	RT	Mass	Abund	Formula	Tgt Mass	Diff (ppm)	Hits (DB)
Cpd 1: C28 H53 N3 O4; 0.208	0.208	495.4015	1400591	C28 H53 N3 O4	495.4036	-3.28	1

Compound Label	*m/z*	RT	Algorithm	Mass
Cpd 1: C28 H53 N3 O4; 0.208	496.4083	0.208	Find by Formula	495.4015

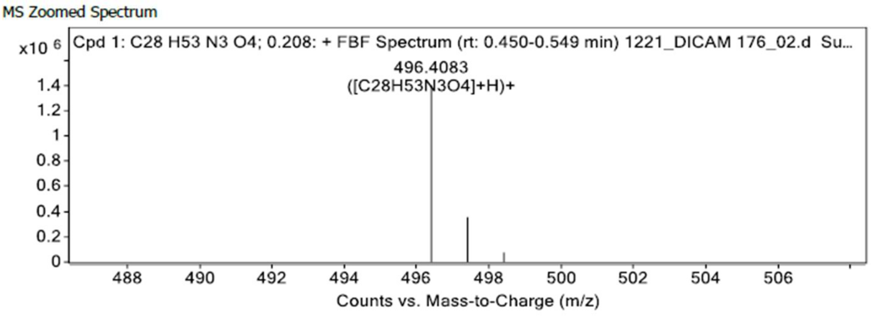

HMRS spectra of DICAM 1**8**

Compound Table

Compound Label	RT	Mass	Abund	Formula	Tgt Mass	Diff (ppm)	Hits (DB)
Cpd 1: C41 H66 N4 O9; 1.825	1.825	758.4825	283770	C41 H66 N4 O9	758.483	-0.64	1

Compound Label	*m/z*	RT	Algorithm	Mass
Cpd 1: C41 H66 N4 O9; 1.825	781.472	1.825	Find by Formula	758.4825

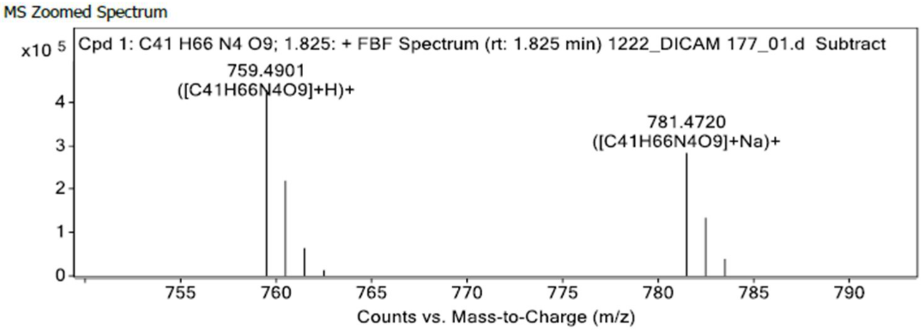

HMRS spectra of DICAM 19

Compound Table

Compound Label	RT	Mass	Abund	Formula	Tgt Mass	Diff (ppm)	Hits (DB)
Cpd 1: C33 H60 N4 O7; 2.481	2.481	624.4449	194966	C33 H60 N4 O7	624.4462	-2.09	1

Compound Label	*m/z*	RT	Algorithm	Mass
Cpd 1: C33 H60 N4 O7; 2.481	625.4522	2.481	Find by Formula	624.4449

MS Zoomed Spectrum

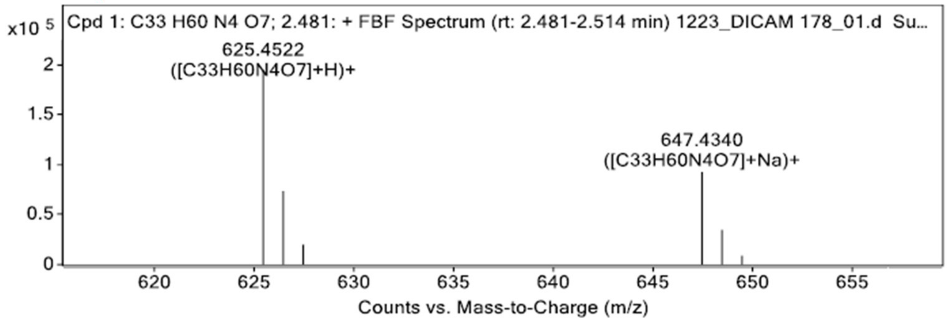

HMRS spectra of DICAM 20

Compound Table

Compound Label	RT	Mass	Abund	Formula	Tgt Mass	Diff (ppm)	Hits (DB)
Cpd 1: C41 H69 N5 O7; 1.981	1.981	743.5177	653206	C41 H69 N5 O7	743.5197	-2.68	1

Compound Label	*m/z*	RT	Algorithm	Mass
Cpd 1: C41 H69 N5 O7; 1.981	744.5256	1.981	Find by Formula	743.5177

MS Zoomed Spectrum

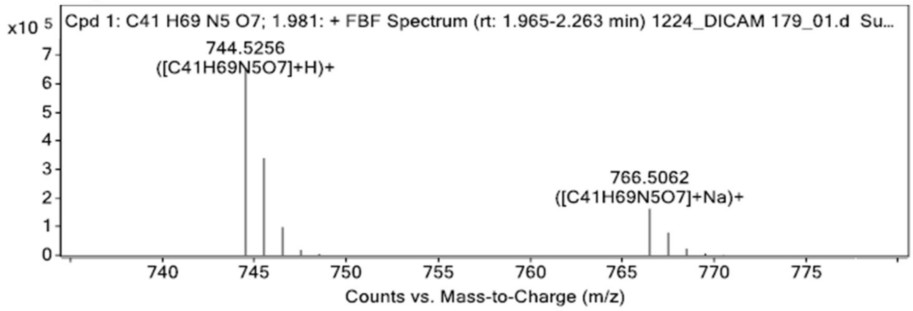

HMRS spectra of DICAM 21

Compound Table

Compound Label	RT	Mass	Abund	Formula	Tgt Mass	Diff (ppm)	Hits (DB)
Cpd 1: C33 H63 N5 O5; 1.748	1.748	609.4823	415713	C33 H63 N5 O5	609.4829	-1.09	1

Compound Label	*m/z*	RT	Algorithm	Mass
Cpd 1: C33 H63 N5 O5; 1.748	610.4898	1.748	Find by Formula	609.4823

MS Zoomed Spectrum

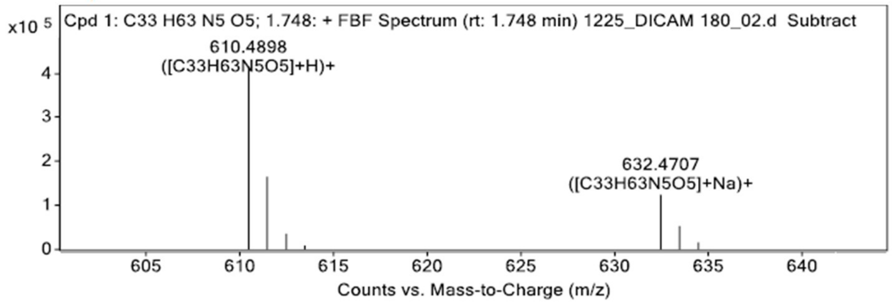

HMRS spectra of DICAM 22

Compound Table

Compound Label	RT	Mass	Abund	Formula	Tgt Mass	Diff (ppm)	Hits (DB)
Cpd 1: C33 H63 N5 O5; 0.272	0.272	609.4806	982448	C33 H63 N5 O5	609.4829	-3.84	1

Compound Label	*m/z*	RT	Algorithm	Mass
Cpd 1: C33 H63 N5 O5; 0.272	610.4889	0.272	Find by Formula	609.4806

MS Zoomed Spectrum

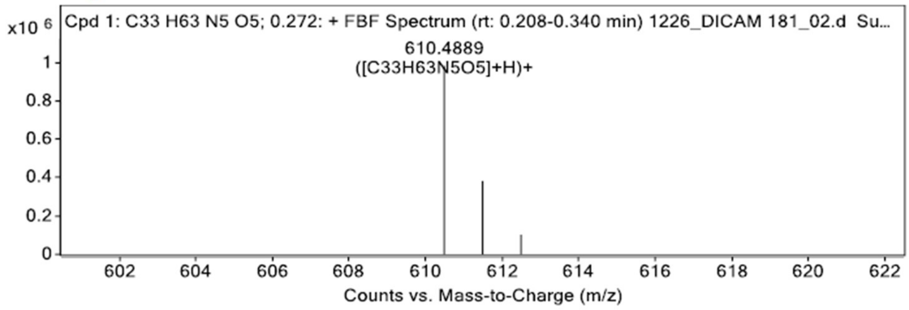

HMRS spectra of DICAM 23

Compound Table

Compound Label	RT	Mass	Abund	Formula	Tgt Mass	Diff (ppm)	Hits (DB)
Cpd 1: C33 H60 N4 O6; 0.242	0.242	608.4488	1186417	C33 H60 N4 O6	608.4513	-3.12	1

Compound Label	*m/z*	RT	Algorithm	Mass
Cpd 1: C33 H60 N4 O6; 0.242	631.4392	0.242	Find by Formula	608.4488

MS Zoomed Spectrum

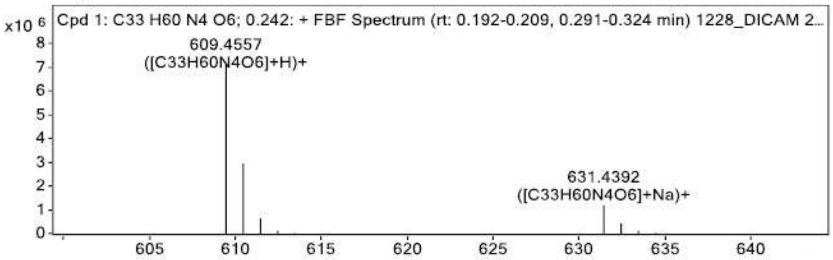

HMRS spectra of DICAM 24

S6 References

Durfee, T., Nelson, R., Baldwin, S., Plunkett III, G., Burland, V., Mau, B., et al. (2001). The complete genome sequence of *Escherichia coli* DH10B: Insights into the biology of a laboratory workhorse, J. Bacteriol. 190, 2597–2606. doi.org/10.1128/jb.01695-07.

Hoskins, J., Alborn Jr., W.E., Arnold, J., Blaszczak, L.C., Burgett, S., DeHoff, B.S., et al. (2001). Genome of the bacterium *Streptococcus pneumoniae* strain R6, J. Bacteriol. 183, 5709–5717. doi.org/10.1128/jb.183.19.5709-5717.2001.

Lanie, J.A., Fu, D.-J., Kamierczak, K.M., Andrezejewski, T.M., Davidsen, T.M., Wayne, K.J., et al. (2007). Genome sequence of Avery's virulent serotype 2 strain D39 of *Streptococcus pneumoniae* and comparison with that of unencapsulated laboratory strain R6, J. Bacteriol. 189, 38–51. doi.org/10.1128/jb.01148-06.

Moscoso, M, García, E., López, R. (2006). Biofilm formation by *Streptococcus pneumoniae*: role of choline, extracellular DNA, and capsular polysaccharide in microbial accretion, J. Bacteriol. 188, 7785–7795. doi:10.1128/JB.00673-06.

Ramos-Sevillano, E., Rodríguez-Sosa, C., Díez-Martínez, R., Olmedillas, M.-J., García, P., García, E., et al. (2012). Macrolides and β-lactam antibiotics enhance c3b deposition on the surface of multidrug-resistant *Streptococcus pneumoniae* strains by a LytA autolysin-dependent mechanism, Antimicrob. Agents Chemother. 56, 5534–5540. doi.org/10.1128/aac.01470-12.
